# Differential roles for DNAJ isoforms in HTT-polyQ and FUS aggregation modulation revealed by chaperone screens

**DOI:** 10.1038/s41467-022-27982-w

**Published:** 2022-01-26

**Authors:** Kinneret Rozales, Amal Younis, Naseeb Saida, Anatoly Meller, Hodaya Goldman, Lior Kellerman, Ronit Heinrich, Shai Berlin, Reut Shalgi

**Affiliations:** 1 Department of Biochemistry, Rappaport Faculty of Medicine, Technion–Israel Institute of Technology, Haifa, 31096 Israel; 2 Department of Neuroscience, Rappaport Faculty of Medicine, Technion–Israel Institute of Technology, Haifa, 31096 Israel

**Keywords:** Fluorescence imaging, Protein aggregation, Chaperones, Chaperones

## Abstract

Protein aggregation is a hallmark of neurodegeneration. Here, we find that Huntington’s disease-related HTT-polyQ aggregation induces a cellular proteotoxic stress response, while ALS-related mutant FUS (mutFUS) aggregation leads to deteriorated proteostasis. Further exploring chaperone function as potential modifiers of pathological aggregation in these contexts, we reveal divergent effects of naturally-occurring chaperone isoforms on different aggregate types. We identify a complex of the full-length (FL) DNAJB14 and DNAJB12, that substantially protects from mutFUS aggregation, in an HSP70-dependent manner. Their naturally-occurring short isoforms, however, do not form a complex, and lose their ability to preclude mutFUS aggregation. In contrast, DNAJB12-short alleviates, while DNAJB12-FL aggravates, HTT-polyQ aggregation. DNAJB14-FL expression increases the mobility of mutFUS aggregates, and restores the deteriorated proteostasis in mutFUS aggregate-containing cells and primary neurons. Our results highlight a maladaptive cellular response to pathological aggregation, and reveal a layer of chaperone network complexity conferred by DNAJ isoforms, in regulation of different aggregate types.

## Abstract (web summary)

Here, using quantitative screens in human cells, the authors reveal distinct effects of naturally-occurring DNAJ chaperone isoforms on pathological aggregation of the Huntington’s disease-associated HTT-polyQ and the ALS-related mutant FUS.

## Introduction

Protein aggregation is a hallmark of many neurodegenerative diseases (NDs), such as Alzheimer’s disease, Parkinson’s disease, Huntington’s disease, and amyotrophic lateral sclerosis (ALS)^1,2^. Importantly, while protein aggregation is common to many different NDs, evidence suggests different biophysical properties of aggregates formed in different diseases^3–5^. Specifically, the Huntington’s disease-related Huntingtin protein, which undergoes polyQ expansion in the disease^6^ (HTT-polyQ), aggregates through a process of oligomerization and fibril formation^7,8^, while many ALS-related proteins, including TDP-43 and FUS, form aggregates in a process of aberrant liquid-liquid phase separation^9,10^.

Molecular chaperones are the master regulators of protein folding and aggregation in the cell^11^. As such, they have been the focus of various studies attempting to understand their role as potential modifiers of ND-related protein aggregation^12^. Of particular interest is the HSP70 network, including a diverse family of HSP40 co-chaperones (also called DNAJs), and Nucleotide Exchange Factors (NEF) co-chaperones, both of which are required for HSP70 to exert its chaperoning function^13^. Interestingly, several chaperone families have undergone expansions throughout evolution, with 13 different HSP70s, and a similar number of different NEFs encoded in the human genome. The DNAJ family of co-chaperones have undergone a major expansion with over 50 different members in humans^13^. These expansions increase the combinatorial capacity of the chaperone network, with the general notion that different DNAJs confer different client specificities to HSP70s, however, for the vast majority of them, this specificity is still unknown^13,14^.

In the context of aggregation, several chaperones have been shown to confer aggregation protection, the majority of them in the context of the HTT-polyQ. Several chaperones have been found to reduce HTT-polyQ aggregation, primarily DNAJB6 and DNAJB8^15–19^, and a few others^20–23^. In ALS, however, and in particular for the ALS-related FUS, chaperone modifiers were much less explored. The yeast HSP104 chaperone was highlighted as a disaggregase of FUS and TDP-43 in yeast^24^. Using over-expression and deletion screens in yeast, significant FUS aggregation modifiers were found to include RNA-binding proteins and proteins involved in stress granules^25^. Nevertheless, WT FUS showed similar aggregation and toxicity to mutFUS in both yeast and fly models^25,26^, underscoring the importance of exploring mutant FUS (mutFUS) aggregation modulation in a mammalian system.

Here we show that cells respond differently to different aggregate types; while HTT-polyQ aggregation elicits a proteotoxic stress response, mutFUS aggregation leads to deteriorated proteostasis. These findings have led us to explore the function of different chaperones, focusing on the HSP70 network, using aggregation modulation screens in human cells, in order to unravel functional diversification in the context of the regulation of different aggregate types.

## Results

### HTT-polyQ aggregation induces a proteotoxic stress response while mutFUS aggregation leads to deteriorated proteostasis

**Fig. 1:**
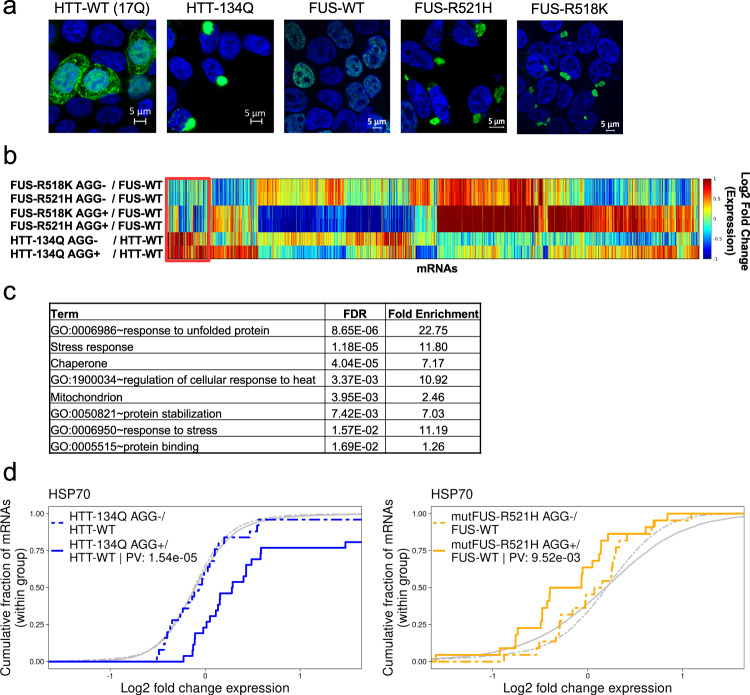
HTT-polyQ aggregation elicited a cellular proteotoxic stress response while mutFUS led to deteriorated proteostasis. **a** HEK293T cells transfected with HTT-17Q-GFP (HTT-WT), HTT-134Q-GFP, FUS-WT-YFP, FUS-R521H-YFP or FUS-R518K-YFP, were imaged 48 h following transfection using confocal microscopy. Cells were stained with DAPI to mark nuclei (blue). Shown are representative images from *n* = 5/14/1 biologically independent experiments for HTT (-WT and -134Q)/FUS (-WT and -R521H)/FUS-R518K, respectively. **b** Hierarchical clustering analysis of RNA-seq Log2 Fold Change (LFC) values of HTT-134Q and mutFUS aggregation experiments, where aggregate-containing cells (AGG+) and non-aggregated mutant protein cells (AGG−) were sorted using FACS. Each mutant was compared to its respective WT which had undergone the same sorting procedure (see “Methods”). **c** Pathway enrichment analysis for the red cluster showed enrichment of groups related to proteotoxic stress responses and chaperones. **d** Expression of genes belonging to the HSP70 family was significantly induced in cells containing HTT-134Q aggregates, and significantly reduced in mutFUS aggregate-containing cells. Plots show the cumulative distribution function (CDF, *y*-axis) of the LFC values of mRNAs belonging to the HSP70 family defined in Brehme et al.^52^ (see “Methods”), and are shifted compared to the background CDF of all expressed mRNAs (in gray) in the HTT-134Q AGG+ sample (solid blue) and FUS-R521H-YFP AGG+ sample (solid yellow), but not in the HTT-134Q AGG- sample (dashed blue) or the mutFUS AGG− samples (dashed yellow). CDF plots for FUS-R518K-YFP showed the same trend (Supplementary Fig. 2e). *P*-values for the differences of each sample compared to the background distribution were calculated using t-test, and indicated in the legend when significant.

We first sought to understand whether cells sense the presence of pathological aggregates of different types in the same way, and if these aggregates elicit similar cellular responses. To that end, we performed RNA-seq transcriptome analysis of HEK293T cells expressing two types of pathological aggregates, the Huntington’s disease associated HTT-polyQ, and the ALS-related mutant FUS. To obtain a pure population of aggregate-containing cells, we used the PulSA method^27^, originally developed for detection of HTT-polyQ aggregation using flow cytometry (FACS), to sort aggregate-containing cells (AGG+) from cells containing diffused mutant protein (AGG−), for both fluorescently tagged HTT-134Q and mutFUS (see “Methods”). FACS analysis of HEK293T cells ectopically expressing the HTT-134Q-GFP disease-related protein (specifically exon1 of the HTT protein, see Supplementary Fig. 1a) gave rise to two cell populations, corresponding to AGG+ and AGG− cells, whereas cells expressing the WT version (HTT-17Q-GFP, also denoted HTT-WT) presented a single fluorescent population, with an effective fraction of AGG+ cells of zero (Supplementary Fig. 1b). For mutFUS, we used two FUS mutations that occur in familial ALS patients, R521H, a fairly common mutation^28,29^, and R518K^30^, both located in the atypical Nuclear Localization Signal (PY-NLS) region of the protein. While FUS-WT-YFP largely localized to the nucleus, these mutants formed perinuclear aggregates (Fig. 1a). We adapted the FACS-based readout for detection of mutFUS aggregation by defining an AGG+ population in FUS-R521H-YFP expressing cells that did not appear in cells expressing FUS-WT-YFP (Supplementary Fig. 1c). HTT-17Q-GFP or FUS-WT-YFP expressing cells served as controls in the RNA-seq and underwent the same sorting procedure. Differential expression analysis revealed 1867 mRNAs that were differentially expressed between either AGG+ (aggregate-containing) or AGG- (diffused) cells and their respective WT form, with 884 and 1252 mRNAs identified for HTT-134Q and mutFUS respectively (see “Methods”, Supplementary Data 1a). Differentially expressed mRNAs were then subjected to clustering analysis (Fig. 1b). While marked changes were observed in cells expressing mutFUS aggregates of both FUS mutations, pathway analysis revealed a single cluster (Fig. 1b, red cluster) that was enriched for proteotoxic stress response pathways, including chaperones, regulation of cellular response to heat and more (Fig. 1c, Supplementary Data 1a). Surprisingly, this cluster contained mRNAs that were induced only in cells expressing HTT-134Q, but were actually repressed in cells expressing mutFUS aggregates. Closer examination of chaperones showed that their mRNAs were indeed significantly induced in cells containing HTT-134Q aggregates, but reduced in mutFUS expressing cells (Supplementary Fig. 2a, b). In fact, the HSP70 chaperone family, showed significant repression in the presence of mutFUS aggregates (Fig. 1d, Supplementary Fig. 2d, e). This indicated that while cells sensed HTT-polyQ aggregation as a proteotoxic assault, the presence of mutFUS aggregates caused the downregulation of chaperones, whose role is to cope with misfolding and aggregation. These results raised the question of whether aggregate-containing cells benefit from induction of chaperones, and furthermore, are there any specific chaperones that may act to protect cells from different aggregate types, but particularly in the case of mutFUS.

### Chaperone network screen for modulators of HTT-polyQ aggregation

**Fig. 2:**
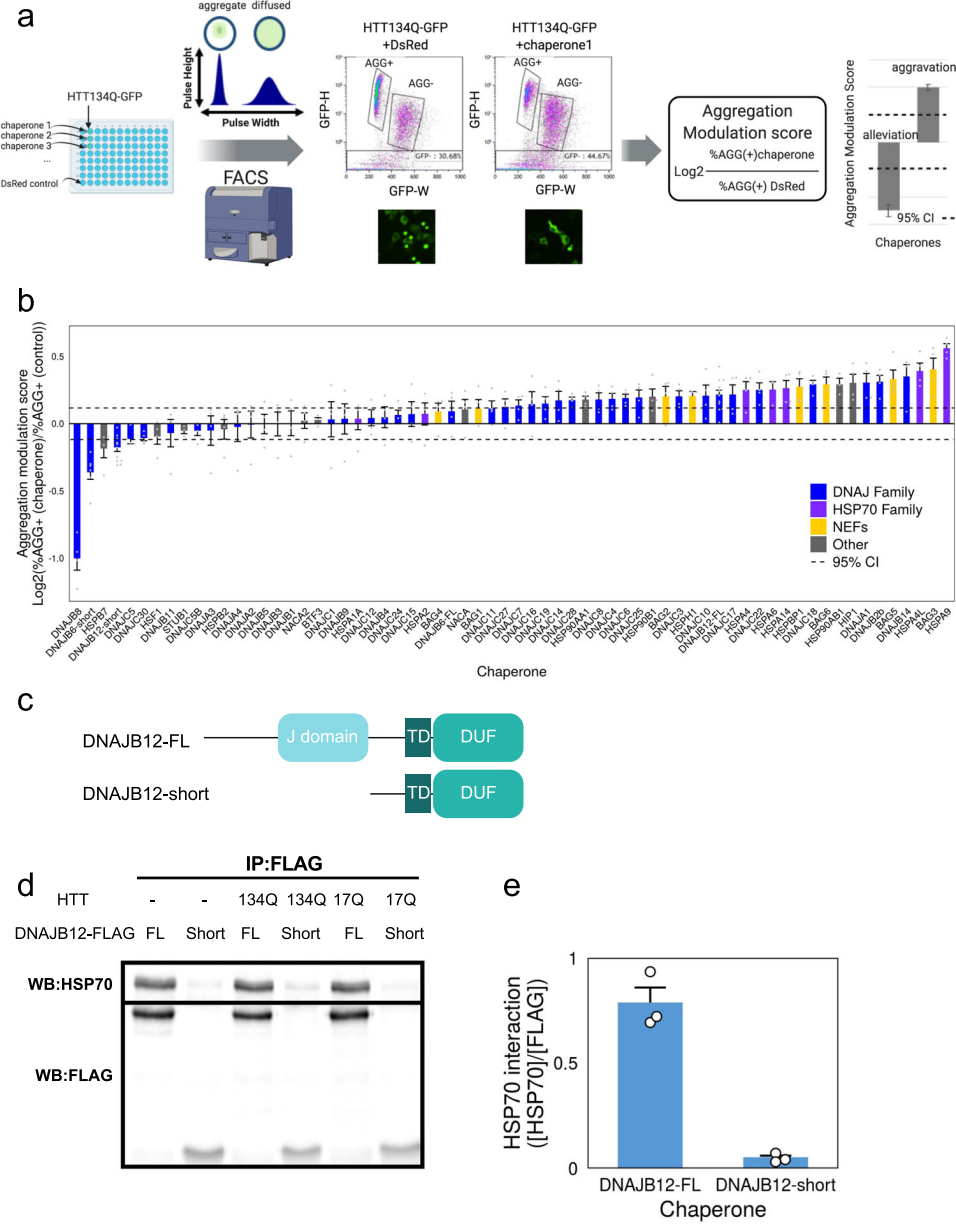
DNAJB12 isoforms with differential effects on HTT-polyQ aggregation identified in a chaperone aggregation modulation screen. **a** Schematic cartoon of the screening framework for chaperone modulators of HTT-polyQ aggregation. HTT-134Q-GFP was co-expressed in HEK293T cells together with each chaperone (Supplementary Table 1), or with DsRed as control. Then, samples were subjected to FACS analysis using the PulSA assay^27^, considering the fluorescent peak height and width, to obtain the fraction of aggregate-containing cells (AGG+ population). An aggregation modulation score was then calculated for each chaperone, and a 95% confidence interval (95% CI, dashed horizontal lines) was calculated according to the variation between all DsRed control replicates (corresponding to ±2*STD). A negative Aggregation Modulation Score denotes aggregation alleviation, while a positive score denotes aggregation aggravation. Created with BioRender.com. **b** Aggregation modulation scores calculated for 66 chaperones showed many modulators that significantly aggravated HTT-134Q-GFP aggregation, while four chaperones significantly protected from HTT-134Q-GFP aggregation. Data are presented as mean values ± SEM of *n* = 4 independent biological replicates for each chaperone, except for DNAJB12-short and DNAJB12-FL (*n* = 12), DNAJB6-short (*n* = 6), HSPA6 and HSP90AB1 (*n* = 3). Gray dots represent individual data points. 95% confidence intervals (dashed lines) were calculated based on *n* = 57 DsRed replicates (CI = ± 0.1168, corresponding to ±2*STD). Chaperone families are color coded. Source data are provided as a Source data file. **c** Schematic diagram of DNAJB12 isoforms. The J domain is absent in the DNAJB12-short. TD—Transmembrane domain, DUF—Domain of unknown function. **d**, **e** Co-immunoprecipitation (co-IP) showed the interactions of DNAJB12 isoforms with HSP70. HEK293T cells were transfected with DNAJB12-FL or -short tagged with FLAG, either alone or in the presence of HTT-134Q-GFP or HTT-17Q-GFP. Cells were subjected to co-IP 48 h later using an anti-FLAG antibody, followed by western blot with anti-HSC70/HSP70 antibody. Western with anti-FLAG antibody was performed to normalize for the amount of pulled-down chaperones. DNAJB12-FL, which contains a J-domain, interacted with HSP70 while the short isoforms lacking the J-domain showed negligible interaction. Quantification was performed using Fiji (**e**), data are presented as mean values ± SEM for *n* = 3 biologically independent samples. Source data are provided as a Source data file.

To systematically characterize the functional effects of individual chaperones on protein aggregation, we first established a functional screening system for modifiers of ND-related protein aggregation phenotype in human cells. To that end, we calibrated PulSA^27^ to be used as a readout for a chaperone screen (Fig. 2a). We then turned to identify potential HTT-134Q chaperone modulators in a co-expression screen, using a standardized co-expressed neutral protein, DsRed, as control (Supplementary Fig. 3a, b, see “Methods”). The aggregate-containing cell fraction (AGG+) resulting from the co-expression of each chaperone together with HTT-134Q-GFP was normalized to a set of DsRed co-expression controls, and the log2 fold change of the AGG+ fraction compared to the control was denoted as the “Aggregation modulation score” (Fig. 2a). Many biological replicates of the control were used to generate a 95% confidence interval (see “Methods”), allowing us to assess whether a chaperone had a significant effect on protein aggregation. Chaperones with a negative Aggregation modulation score, i.e., below the confidence interval, are those that provided significant protection from aggregation, whereas chaperones with a positive Aggregation modulation score above the confidence interval, significantly aggravated protein aggregation (Fig. 2a).

### Different DNAJB12 isoforms show differential effects on HTT-polyQ aggregation

Next, we systematically co-expressed HTT-134Q-GFP together with each of a set of 66 chaperones that were tagged with FLAG, focusing mainly on the network of HSP70 chaperones and their co-chaperones (Supplementary Table 1). Since we found that HSP70s levels were induced in response to HTT-polyQ aggregation, and repressed in the presence of mutFUS aggregates (Fig. 1d, Supplementary Fig. 2d), this network was of particular interest. While control cells yielded around 34% HTT-134Q-GFP aggregate-containing cells, co-expression of DNAJB8 reduced the fraction of HTT-134Q-GFP aggregate-containing cells by 50% on average, and as much as ~13% (Fig. 2b, Supplementary Fig. 3c). These FACS-based results were confirmed by microscopy imaging, illustrating reduced aggregate formation in DNAJB8 expressing cells (Supplementary Figs. 3d, 4a, b).

Overall, our chaperone co-expression screen revealed a continuous spectrum of Aggregation modulation scores (Fig. 2b), with ~40% of the chaperones significantly aggravating HTT-134Q-GFP aggregation phenotype, and four chaperones that provided significant protection from HTT-134Q-GFP aggregation. Notably, Aggregation modulation scores were not explained by the degree of chaperone overexpression (Supplementary Fig. 3e), and showed no correlation with endogenous chaperone levels (Supplementary Fig. 3f), or with chaperones fold changes in response to HTT-134Q-GFP aggregation (Supplementary Fig. 3g). Importantly, the top three chaperones that we found to significantly reduce HTT-134Q aggregation, DNAJB8, DNAJB6-short and HSPB7 (Fig. 2b, Supplementary Fig. 4a–c), were all previously identified using biochemical methods to provide HTT-polyQ aggregation protection in human cells^15,16,23^. Conversely, and unexpectedly, our screen identified many chaperones that increased HTT-polyQ aggregation. For example, co-expression of HSP90s aggravated HTT-134Q-GFP aggregation. Cells co-expressing HSP90AB1 showed on average 1.22 fold more aggregate-containing cells than the respective controls (Fig. 2b, Supplementary Figs. 3c, d, 4a, d). This result is in agreement with a previous report on the alleviation of HTT-polyQ aggregation with HSP90 inhibitors^31^. Additionally, DNAJB2b was previously shown by Hageman et al. to increase HTT-polyQ aggregation^16^, while DNAJA1 knockout was found to reduce HTT-polyQ aggregation^32^. DNAJB2b and DNAJA1 were both identified as aggravators of HTT-134Q-GFP aggregation in our screen, in agreement with these previous studies.

Interestingly, some chaperones had different isoforms, denoted full-length (FL) and short, with differential effects on HTT-polyQ aggregation. Our screen found one chaperone with a previously unidentified significant rescue of HTT-polyQ aggregation (Fig. 2b): DNAJB12-short, a naturally occurring short isoform of DNAJB12. Specifically, this short DNAJB12 isoform lacks the J-domain responsible for HSP70 interactions of all J-proteins (Fig. 2c). Indeed, while the full-length isoform DNAJB12-FL strongly interacted with HSP70, the short DNAJB12 isoform did not show HSP70 interactions in co-immunoprecipitation (co-IP) experiments (Fig. 2d, e). Surprisingly, DNAJB12-FL showed the opposite effect from DNAJB12-short, as it significantly elevated HTT-polyQ aggregation (Fig. 2b, Supplementary Fig. 4e). Therefore, it seems that different isoforms of DNAJB12 showed opposing functional effects on HTT-polyQ aggregation.

### Modulators of ALS-related mutant FUS aggregation are distinct from those of HTT-polyQ

**Fig. 3:**
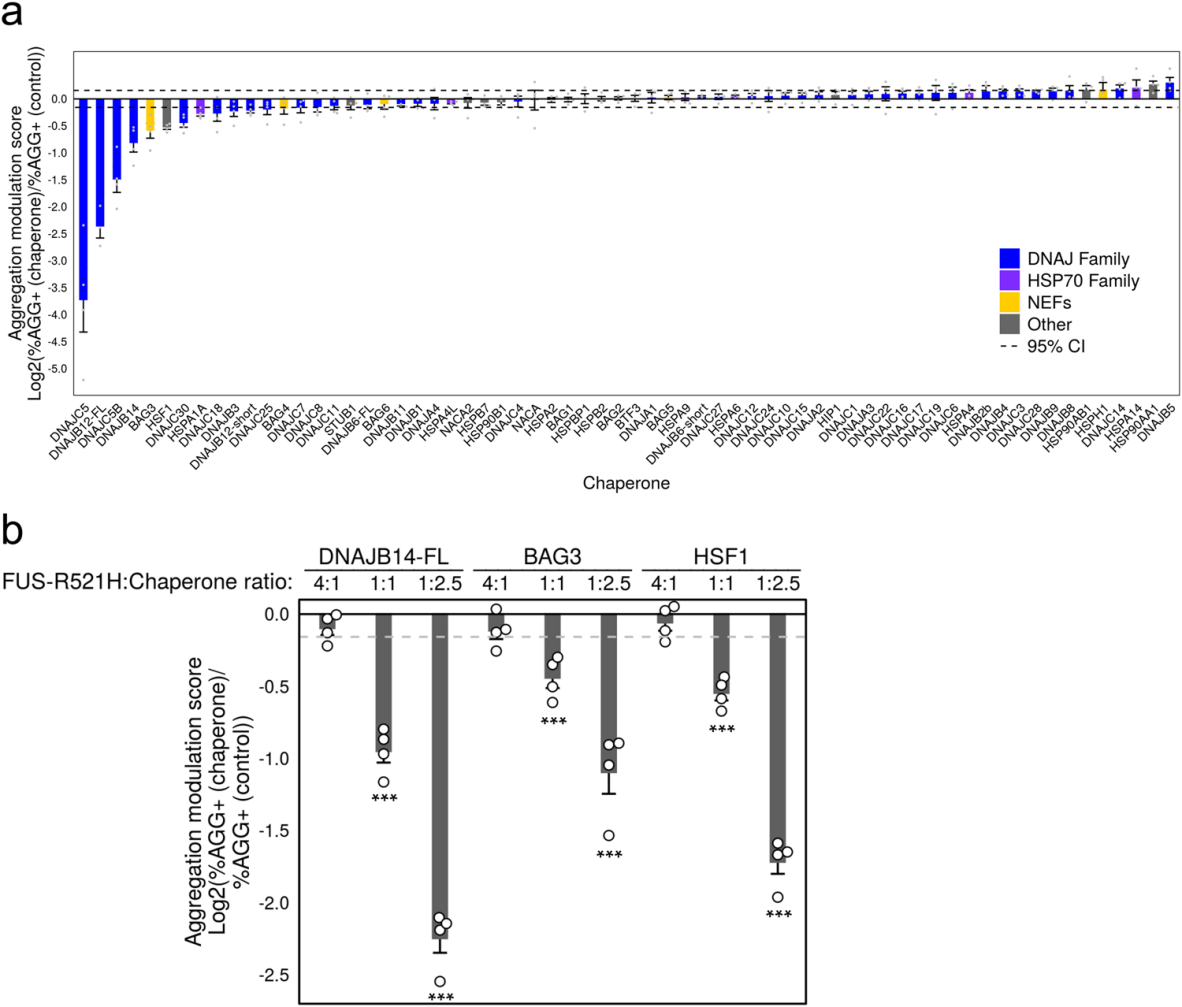
A chaperone network screen identified modulators of mutant FUS aggregation. **a** Aggregation modulation scores for 66 chaperones revealed aggregation alleviators of mutFUS aggregation (negative scores, left side). Data are presented as mean values ± SEM of *n* = 4 biologically independent experiments for all chaperones, except for HSPA6 and DNAJB6-short (*n* = 3). Gray dots represent individual data points. 95% confidence intervals marked with dashed lines (CI = ± 0.1577, representing 2*STD based on a total of *n* = 90 DsRed replicates). Chaperone families are color coded. Source data are provided as a Source data file. **b** Aggregation modulation showed a dose-dependent effect. Cells were co-transfected with different FUS-R521H-YFP:chaperone ratios, starting from high ratios of mutFUS DNA:chaperone DNA (2000 ng/500 ng, respectively), equal DNA amounts as in the screen (1250 ng/1250 ng), and a low ratio (700 ng/1800 ng, respectively). Results demonstrated a dose-dependent rescue for all chaperones shown. Data are presented as mean values ± SEM of *n* = 4 biologically independent experiments. Dashed lines represent 95% CI as in (**a**). ***p < 0.003, empirical p-value (see “Methods”). Source data are provided as a Source data file.

Next, we turned to explore chaperone functional modulation of the ALS-related protein FUS^33^, which has not been previously explored in this context. Here too, DsRed co-expression was chosen as a baseline control for the functional screen (Supplementary Fig. 5a, see “Methods”). The aggregation modulation screen was then performed for FUS-R521H-YFP using co-expression of each of the 66 chaperones in our examined HSP70 network (Fig. 3a). Interestingly, while the majority of chaperones did not significantly alter FUS-R521H-YFP aggregation phenotype, two chaperones, HSP90AA1 and DNAJB5 showed a slight but significant aggravation of FUS-R521H-YFP aggregation. Furthermore, our screen revealed eight chaperones that significantly protected from FUS-R521H-YFP aggregation, out of which seven gave a substantial rescue of more than 25% (Aggregation modulation score < −0.41, Supplementary Table 1, Fig. 3a). We note that the rescue significance was robust to the FACS parameter settings (Supplementary Fig. 5b). As for HTT-polyQ, mutFUS Aggregation modulation scores were also not explained by the degree of chaperone overexpression (Supplementary Fig. 5c), and showed no correlation with endogenous chaperone levels (Supplementary Fig. 5d) or with chaperone expression fold changes in response to mutFUS aggregation (Supplementary Fig. 5e). Interestingly, comparison between HTT-polyQ and mutFUS Aggregation modulation scores showed that FUS-R521H-YFP aggregation modulators were completely different than those of HTT-polyQ aggregation, and the correlation between HTT-polyQ and mutFUS Aggregation modulation scores was negligible (Supplementary Fig. 5f).

We next checked the extent to which top chaperones affected cell viability. Among the top mutFUS modulators, three chaperone, DNAJC5, DNAJB12-FL, and DNAJC5B, had a negative effect on cell viability of 15% or more (Supplementary Fig. 5g, h). We, therefore, further characterized a few of the other aggregation protective chaperones. Microscopy imaging of DNAJB14, HSF1, and BAG3 co-expressing cells confirmed less aggregation of FUS-R521H-YFP compared to the control (Supplementary Fig. 5i). Additionally, these three FUS-R521H-YFP aggregation protective chaperones were also able to rescue the aggregation phenotype of the FUS-R518K-YFP mutant, to a similar extent (Supplementary Fig. 5j, k).

Importantly, we found FUS-R521H-YFP aggregation protection to be dose-dependent (Fig. 3b, see “Methods”). For example, while the Aggregation modulation score obtained for DNAJB14 was originally −1, reflecting a 50% reduction in the fraction of FUS-R521H-YFP aggregate-expressing cells, higher doses of this chaperone promoted a 78% reduction in FUS-R521H-YFP aggregation phenotype (Fig. 3b).

### Differential roles for DNAJB14 isoforms in mutant FUS aggregation protection

**Fig. 4:**
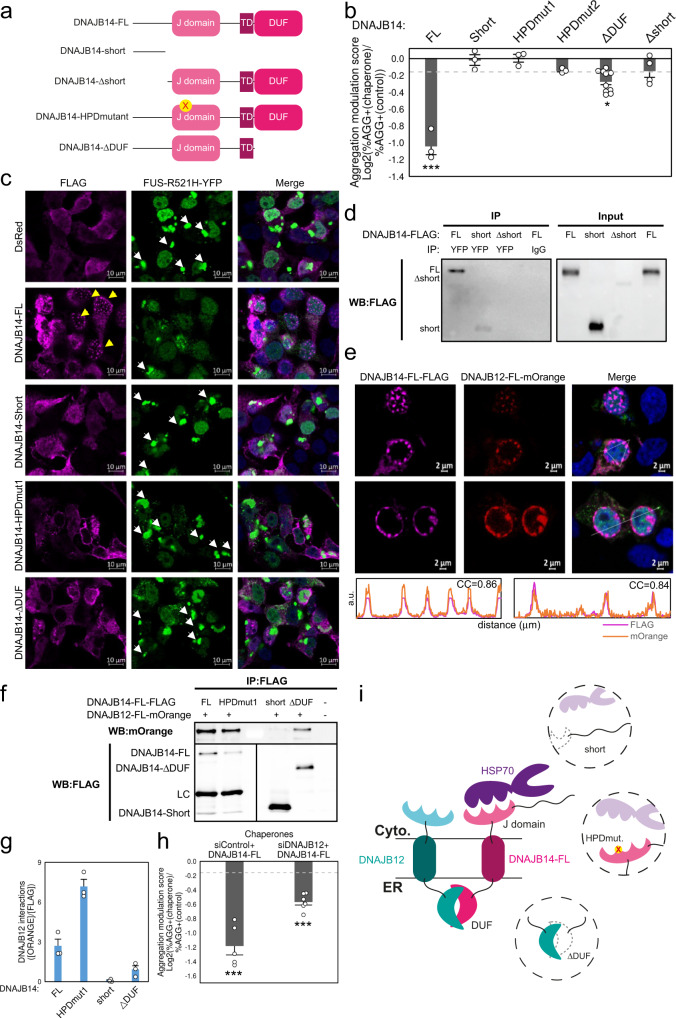
Differential roles for DNAJB14 isoforms in mutant FUS aggregation protection. **a** DNAJB14 isoforms and mutants. The J domain, TD (Transmembrane domain) and DUF (Domain of unknown function) are absent in the naturally-occurring DNAJB14-short isoform. The Δshort lacks the N-terminal part encoded by DNAJB14-short. **b** FUS-R521H-YFP Aggregation modulation scores for DNAJB14 isoforms. Data are presented as mean values ± SEM of *n* = 3/3/3/3/10/4 biologically independent experiments for DNAJB14-FL/-short/-HPDmut1/-HPDmut2/-ΔDUF/-Δshort. Dashed lines represent 95% CIs as in Fig. 3a. ***p < 0.003, *p < 0.05, empirical *p*-value (see “Methods”). Source data are provided as a Source data file. **c** FLAG-tagged DNAJB14 isoforms co-expressed with FUS-R521H-YFP (green). Immunofluorescence imaging (IF) with anti-FLAG antibody (pink), DAPI marks nuclei (blue). Yellow arrows mark DJANGO structures. White arrows mark mutFUS aggregates. Shown are representative images out of *n* = 5/5/4/4/2 biologically independent experiments, for DsRed/DNAJB14-FL/-short/-HPDmut1/-ΔDUF. FUS-WT-YFP IF images shown in Supplementary Fig. 6b, l, 7d, e. **d** Co-IP showed DNAJB14-FL interaction with mutFUS. Densitometry quantification of *n* = 4 biologically independent experiments is shown in Supplementary Fig. 6e. Source data are provided as a Source data file. **e** Immunofluorescence images of DNAJB12-FL-mOrange and DNAJB14-FL-FLAG showing co-localization within DJANGOs. Cross correlation plots shown with correlation coefficient (CC) between DNAJB14 and DNAJB12. See Supplementary Fig. 6n for additional images. *n* = 3. **f**, **g** Co-IP (**f**) between DNAJB12-FL-mOrange and DNAJB14 isoforms, showing negligible interaction of DNAJB12-FL with DNAJB14-short, and reduced interaction with DNAJB14-ΔDUF. LC— light chain. White band (lane 3)—molecular weight marker of 70KDa. **g** Quantification of interaction performed using Fiji, densitometry of mOrange bands were normalized to the corresponding FLAG bands (see “Methods”). Data are presented as mean values ± SEM of *n* = 3 biologically independent samples (additional replicates shown in Supplementary Fig. 6p). Source data are provided as a Source data file. **h** FUS-R521H-YFP Aggregation modulation scores in cells expressing DNAJB14-FL that underwent DNAJB12 knockdown (siDNAJB12 + DNAJB14-FL) vs. siControl cells (siControl + DNAJB14-FL). Data are presented as mean values ± SEM of *n* = 5/6 biologically independent samples (for siControl/siDNAJB12 respectively). Dashed lines represent 95% CIs as in Fig. 3a. ***p < 0.003, empirical *p*-value (see “Methods”). Source data are provided as a Source data file. **i** DNAJB14 isoforms and mutants and their interactions with DNAJB12-FL and HSP70.

DNAJB12-short, which we identified as a modulator of HTT-polyQ aggregation above (Fig. 2b), provided only marginal rescue of FUS-R521H-YFP aggregation (Fig. 3a). Interestingly, DNAJB14 also has a naturally-occurring short isoform, lacking the J-domain, the DUF and the transmembrane domain (TD) (Fig. 4a). Moreover, DNAJB14 has been previously shown to interact and co-localize with DNAJB12^34^. We next cloned the short isoform of DNAJB14 (Fig. 4a, see “Methods”) and tested its functional effect on FUS-R521H-YFP aggregation. In contrast to the significant protection by DNAJB14-FL, DNAJB14-short did not affect FUS-R521H-YFP aggregation (Fig. 4b). These results were independent of the FLAG tag (Supplementary Fig. 6a), and were further confirmed by image analysis of FUS-R521H-YFP aggregation in immunofluorescence images containing hundreds of cells (Fig. 4c, Supplementary Fig. 6b, c, see “Methods”). We tested the functional effects of the two DNAJB14 isoforms on additional ALS-associated FUS mutants: the common mutant R521C^35^, the R518K mutant, and two additional ALS mutations located at the C-terminal RGG domain of FUS (see “Methods”). In all cases, DNAJB14-FL conferred substantial aggregation protection, which was completely absent in the presence of DNAJB14-short (Supplementary Fig. 6d). In the case of two mutants, R495X and R521C, DNAJB14-FL reduced aggregation markedly, by 79% and 83%, respectively (Supplementary Fig. 6d). Thus, the differential effects of aggregation protection by DNAJB14 isoforms seem to represent a broader phenomenon with potential implications on many FUS mutations.

Using co-IP, we further found that DNAJB14-FL interacted with both mutFUS (Fig. 4d, Supplementary Fig. 6e) and WT-FUS (Supplementary Fig. 6f, g), while DNAJB14-short showed a much lower level of interaction (Fig. 4d, Supplementary Fig. 6e–g). An N-terminal truncation mutant lacking the segment of the short isoform, DNAJB14-Δshort (Fig. 4a), was unable to protect from mutFUS aggregation (Fig. 4b), and did not show any interaction with FUS (Fig. 4d, Supplementary Fig. 6e–g), although its low level of expression may preclude interaction detection (Fig. 4d, Supplementary Fig. 6f). Using live cell imaging of mutFUS expressing cells in the presence of DNAJB14-FL or -short (Supplementary Fig. 6h, see “Methods”), we observed that, in the presence of DNAJB14-FL, aggregates were generated in much lower rates, rather than disappear. These results support the notion that, rather than disaggregation, DNAJB14-FL acts to chaperone FUS and prevent its de-novo aggregation.

### DNAJB14 and DNAJB12 full-length isoforms form a complex

We subsequently examined the potential mutual role of DNAJB12 and DNAJB14 as a complex in the rescue of FUS-R521H-YFP aggregation. Each of these two chaperones contains a single transmembrane domain (TD, Figs. 2c, 4a), and they were reported to be ER localized^36–38^. Indeed, DNAJB12-FL, DNAJB12-short and DNAJB14-FL were co-localized with an ER marker (Supplementary Fig. 6i, see Methods), while DNAJB14-short that lacks the TD, did not (Supplementary Fig. 6i). Immunofluorescence imaging of the four isoforms showed that DNAJB14-FL and DNAJB12-FL were able to generate unique nuclear structures (Fig. 4c, e, Supplementary Fig. 6b, j–l, n), that were previously termed DJANGOs^34^. Neither DNAJB14-short nor DNAJB12-short could form the nuclear DJANGO structures (Fig. 4c and Supplementary Fig. 6b, j–l). Immunofluorescence image analysis using a classifier trained to identify DJANGO structures (see “Methods”) confirmed that while DNAJB14-FL formed DJANGOs in about 10–14% of the cells, and DNAJB12-FL in about 7.5% of the cells, no DJANGOs were formed by DNAJB14-short or DNAJB12-short (Supplementary Fig. 6m). In order to check whether DNAJB14-FL and DNAJB12-FL were co-localized in the cell, we tagged DNAJB12-FL with an mOrange fluorescent protein, and co-expressed it together with the FLAG tagged DNAJB14-FL. Immunofluorescence imaging showed that DNAJB12-FL and DNAJB14-FL co-localized to the same nuclear DJANGO structures (Fig. 4e, Supplementary Fig. 6n). Moreover, these two chaperones were co-localized also in cells in which they both showed an ER localization pattern (Supplementary Fig. 6o). Co-IP experiments confirmed that DNAJB12-FL and DNAJB14-FL physically interacted in cells, while DNAJB14-short interaction with DNAJB12-FL (Fig. 4f, g, Supplementary Fig. 6p), and vice versa (Supplementary Fig. 6q, r), were negligible.

### DNAJB14-mediated protection of mutant FUS aggregation is HSP70 dependent

The fact that the full-length isoform of DNAJB14 substantially protected from mutFUS aggregation while the short isoform did not, hinted at the potential importance of the J-domain, and therefore of HSP70 interactions, in the rescue phenotype. Indeed, while DNAJB14-FL interacted with HSP70, DNAJB14-short did not, as shown by co-IP (Supplementary Fig. 7a, b). To test the role of HSP70 interactions in the rescue phenotype, we generated a DNAJB14-FL version that was mutated in the HPD motif of the J-domain, the region where HSP70 is known to bind J-proteins^13^ (DNAJB14-HPDmut, Fig. 4a, i see “Methods”). This mutant showed negligible binding to HSP70 using co-IP (Supplementary Fig. 7a, b), and this trend was consistent in cells expressing either FUS-WT-YFP or FUS-R521H-YFP (Supplementary Fig. 7a, b). Immunofluorescence imaging of this mutant, as well as a second HPD mutant (DNAJB14-HPDmut2), demonstrated a substantially lower rate of DJANGO formation (Fig. 4c, Supplementary Fig. 7c, d). Surprisingly, co-IP experiments indicated that DNAJB14-HPDmut1 showed enhanced binding to DNAJB12-FL (Fig. 4f, g, Supplementary Fig. 6p). Finally, we tested the functional effects of the HPD mutation on FUS-R521H-YFP aggregation. Our data showed that these mutants lost their ability to protect cells from FUS-R521H-YFP aggregation (Fig. 4b).

Thus, it seems that while HSP70 binding to DNAJB14-FL was not necessary for its interaction with DNAJB12-FL (Fig. 4f), the ability of DNAJB14 to interact with HSP70 was crucial for its function in alleviating FUS-R521H-YFP aggregation.

### DNAJB14 DUF domain plays an important role in the DNAJB14–DNAJB12 complex formation and the protection from mutant FUS aggregation

We next raised the possibility that the DUF domain (Domain of Unknown Function) of DNAJB14 might mediate the interaction between DNAJB14 and DNAJB12. To test this hypothesis, we generated a truncated DNAJB14 version lacking the DUF domain (termed DNAJB14-ΔDUF, Fig. 4a, i, see “Methods”). First, we examined the interaction of the DNAJB14-ΔDUF with HSP70 using co-IP, and found that the removal of the DUF domain reduced the interaction to about 50% (Supplementary Fig. 8a, b). Furthermore, we found that, while DNAJB14-FL strongly interacted with DNAJB12-FL, DNAJB14-ΔDUF interactions with DNAJB12-FL were substantially weaker, about 3 fold lower compared to DNAJB14-FL (Fig. 4f, g, Supplementary Fig. 6p). Moreover, DNAJB14-ΔDUF was also deficient in forming DJANGO structures (Supplementary Fig. 7c). This indicated that the DUF domain is involved in mediating DNAJB14–DNAJB12 interactions. Finally, testing the functional effect of DNAJB14-ΔDUF on FUS-R521H-YFP aggregation phenotype revealed that the removal of the DUF domain severely compromised the rescue of FUS-R521H-YFP aggregation by DNAJB14 (Fig. 4b). Therefore, the DUF domain is both involved in the DNAJB14–DNAJB12 complex formation, as well as in the protection from mutFUS aggregation.

### DNAJB14–DNAJB12 complex inter-dependence in the rescue of mutant FUS aggregation

Our collective evidence thus far supported the notion that it is the complex of DNAJB14–DNAJB12 together with HSP70 that is important for the rescue of FUS-R521H-YFP aggregation. We next examined the role of the interactions between DNAJB14-FL and DNAJB12-FL in the rescue of FUS-R521H-YFP aggregation. To that end, we used siRNA to knock down the endogenous DNAJB12, to about half its original levels (Supplementary Fig. 8c). We then tested the functional effect of DNAJB14-FL ectopic expression on FUS-R521H-YFP aggregation in cells that were knocked down for DNAJB12 (see “Methods”). We found that knockdown of the endogenous DNAJB12 reduced the ability of DNAJB14-FL to rescue FUS-R521H-YFP aggregation by 42% (Fig. 4h). Moreover, a co-expression experiment of both DNAJB12-FL and DNAJB14-FL did not have an additive effect on mutFUS aggregation protection, but rather showed a similar extent of protection as that of DNAJB12-FL alone (Supplementary Fig. 8d), further demonstrating the dependence between the two chaperones in the aggregation protection function. This indicated that the rescue of FUS-R521H-YFP aggregation provided by DNAJB14-FL is dependent on DNAJB12-FL, in addition to its interaction with HSP70.

These results collectively support the notion that the DNAJB14–DNAJB12–HSP70 complex is essential for providing substantial protection from mutFUS aggregation.

### DNAJB14-FL increases the mobility of mutFUS aggregates

**Fig. 5:**
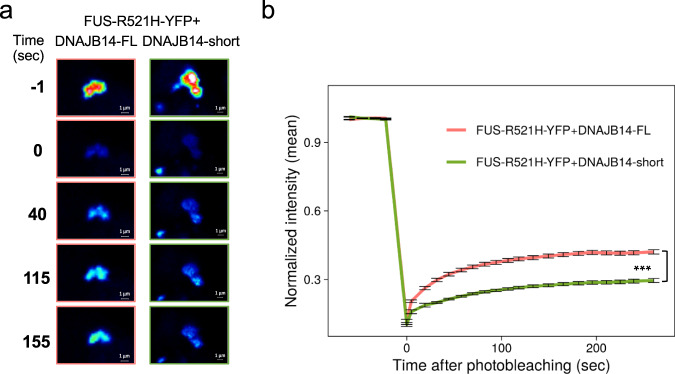
DNAJB14-FL increased the mobility of mutFUS aggregates. **a** FRAP analysis (fluorescence recovery after photobleaching) was performed on mutFUS aggregates in cells co-expressing FUS-R521H-YFP together with either DNAJB14-FL or -short. A single aggregate is shown over time, Time = 0 denotes photobleaching. **b** Normalized intensity values for each aggregate (*n* = 156 for DNAJB14-FL expression and *n* = 150 for DNAJB14-short) were binned by time (20 timepoints per bin) and a median value for each bin was calculated. The intensity values right after photobleaching were used as *t* = 0. For the plot, a median value and a standard error of each bin across all aggregates in cells co-expressing either DNAJB14-FL or DNAJB14-short were calculated. Data are presented as median values ± SEM. T-test (two-sided) was performed for each pair of bins between the DNAJB14-FL and DNAJB14-short values. *P*-values (FDR-corrected) were significant (<0.01) in all bins, accept for the *t* = 0 sec and *t* = 5 sec, ***p = 1.4e−14 at the last bin (260–280 s after photobleaching).

FUS is known to form aggregates in a liquid-liquid phase separation (LLPS) process, while ALS-related mutations have been shown to accelerate this process towards forming more solid inclusions^9^. We thus wanted to examine the functional effects of DNAJB14 isoforms on the mobility of mutFUS inclusions. To that end, we used FRAP analysis (Fluorescence Recovery After Photobleaching) of cells co-expressing FUS-R521H-YFP and either DNAJB14-FL or -short. Analyzing 156 and 150 inclusions in DNAJB14-FL or -short expressing cells (respectively), we found that, while aggregates showed some degree of recovery in both cases, inclusions in DNAJB14-FL co-expressing cells were significantly more mobile than those in DNAJB14-short co-expressing cells, showing on average 42% recovery, compared to 29.5% recovery (Fig. 5a, b, *p* = 6.4e−18). Thus, the co-expression of DNAJB14-FL increases the mobility of mutFUS aggregates.

### DNAJB14-FL restores the deteriorated proteostasis caused by mutFUS aggregation

**Fig. 6:**
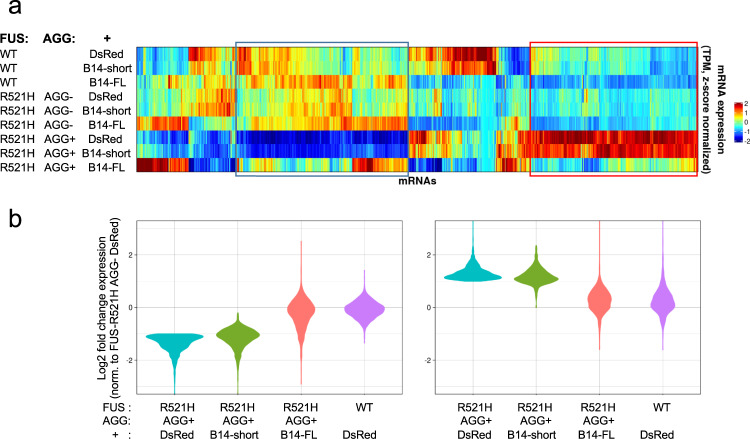
DNAJB14-FL restored deteriorated proteostasis caused by mutFUS aggregation. **a** Hierarchical clustering analysis of RNA-seq mRNA expression (mean TPM of replicates, z-score normalized) in FUS-WT and mutFUS aggregation experiments, where cells were co-transfected with either DNAJB14-FL, DNAJB14-short, or DsRed as control. Aggregate-containing cells (AGG+) and non-aggregated mutFUS cells (AGG-) were FACS-sorted. The two largest clusters in the data (blue and red rectangles) contain mRNAs either induced (red) or repressed (blue) in the presence of mutFUS aggregates in both DsRed and DNAJB14-short co-expressing cells. **b** Distribution plots (violin plots) of the Log2 fold change values of differentially expressed mRNAs (DEGs, see “Methods”) identified in mutFUS aggregate-containing cells (AGG+) co-expressing DsRed (blue). A similar distribution is shown in mutFUS AGG+ DNAJB14-short co-expressing cells (green), while co-expression of DNAJB14-FL in mutFUS AGG+ cells (pink) shows a distribution highly similar to WT-FUS cells (purple), demonstrating restoration of gene expression. Left panel—downregulated DEGs. Right panel—upregulated DEGs, both defined in FUS-R521H-YFP+DsRed, AGG+.

To better understand the underlying causes of mutFUS aggregation protection elicited by DNAJB14-FL, we further performed another RNA-seq experiment, of cells expressing WT or mutFUS in the presence of either of the two DNAJB14 isoforms, or in the presence of DsRed as control. RNA-seq was performed after sorting the cells according to mutFUS aggregation status (as above). Here too, as in Fig. 1b, we observed that mutFUS aggregation elicited marked changes in gene expression (see Supplementary Fig. 9a for comparison between the two datasets). Moreover, this dataset recapitulated our previous observation (Fig. 1d, Supplementary Fig. 2d, e) demonstrating the repression of HSP70 chaperones in aggregated mutFUS cells (Supplementary Fig. 9b). Cells co-expressing DNAJB14-short showed expression patterns which were highly similar to the DsRed co-expressing cells (Fig. 6a, Supplementary Fig. 9c). Clustering analysis of differentially expressed mRNAs (see Methods) demonstrated that the two largest clusters in the data contained mRNAs either induced or repressed in the presence of mutFUS aggregates in DsRed or DNAJB14-short co-expressing cells (Fig. 6a). The repressed group contained many chaperones, as well as several other proteostasis-related genes (Supplementary Data 1b). Strikingly, though, the co-expression of DNAJB14-FL almost completely abrogated the vast majority of these mRNA expression changes (Fig. 6a, b, Supplementary Fig. 9d). Interestingly, DNAJB14-FL also increased the levels of HSP70 chaperones (Supplementary Fig. 9e) and abolished the downregulation of several chaperone families, including HSP60s, HSP90s, and ER-related chaperones (Supplementary Fig. 9f–h). DNAJB14-FL expression has led to the induction of a handful of the classical heat shock response (HSR) chaperones, such as Hspa6, Hspa1a, and Dnajb1, however comparison to an RNA-seq data of cells subjected to heat shock showed little similarity beyond these handful of genes (Supplementary Fig. 9i). To further understand if the induction of these HSR chaperones was responsible for the aggregation protection effects of DNAJB14-FL, we performed a reporter assay using an HSR-inducible promoter (similar to Santagata et al.^39^), to assess the degree of HSR induction elicited by the ectopic expression of each chaperone in our network (see “Methods”). This analysis showed that triggering the HSR did not correlate with mutFUS aggregation protection (Supplementary Fig. 9j), and many other chaperones that induced the reporter to a similar or greater extent than DNAJB14-FL did not show mutFUS aggregation protection (Supplementary Fig. 9j). Additional qPCR experiments further supported this notion (Supplementary Fig. 9l). Moreover, treating mutFUS expressing cells with several regimens of heat shock did not show any aggregation protection (Supplementary Fig. 9m, see “Methods”). Therefore, our data shows that mutFUS aggregation has led to a deterioration in proteostasis, and that co-expression of DNAJB14-FL was able to restore proteostasis, alongside buffering of widespread gene expression changes that occurred in response mutFUS aggregation.

### DNAJB14-FL protects from mutFUS aggregation and restores deteriorated proteostasis in mutFUS-expressing primary neurons

**Fig. 7:**
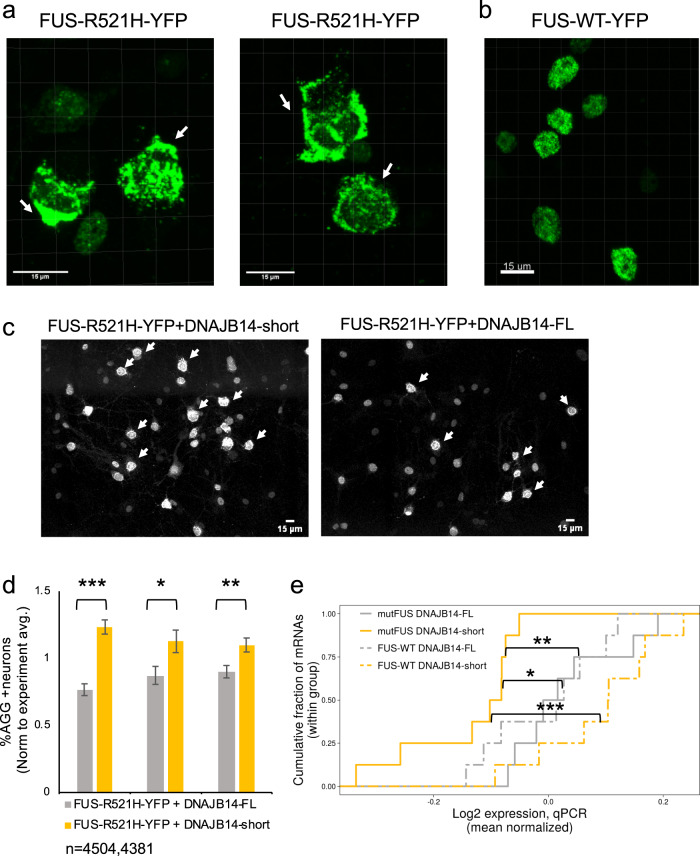
DNAJB14 isoforms showed differential modulation of mutant FUS aggregation in primary neurons, with DNAJB14-FL restoring mutFUS-mediated deteriorated proteostasis. **a**, **b** Confocal microscopy images of live neurons showing a complex FUS-R521H-YFP aggregation pattern (**a**) and live neurons expressing FUS-WT-YFP (**b**). Visualized using Imaris. White arrows mark neurons with FUS-R521H-YFP aggregates. The left panel contains additional cells with a non-aggregated FUS-R521H-YFP expression pattern. Shown are images of representative cells out of *n* = 5/3 biologically independent samples for mutFUS/FUS-WT respectively. **c** Larger fields of neuronal cultures co-infected with FUS-R521H-YFP and either DNAJB14-short (left panel) or DNAJB14-FL (right panel). Shown are maximum-projection of confocal fluorescent images (using Fiji). Additional fields are shown in Supplementary Fig. 10e. White arrows mark neurons with mutFUS aggregates. Shown are representative fields out of *n* = 5 biologically independent samples. **d** Image analysis of thousands of live neurons showed significantly lower FUS-R521H-YFP aggregation in DNAJB14-FL expressing neurons compared to DNAJB14-short. Neuronal cultures co-infected with FUS-R521H-YFP and DNAJB14-FL/DNAJB14-short viruses (equal titers) at day 5 of culture, imaged six days later. Data are presented as mean values ± SEM of the aggregation fraction, normalized to the average aggregation fraction measured overall in each experiment, in *n* = 3 biologically independent experiments. Experiment mean aggregation fraction was calculated including all fields of both DNAJB14-FL and DNAJB14-short samples. Experiments included 12, 24, and 37 images from each of DNAJB14-FL or DNAJB14-short samples, containing a total of *n* = 884 and 1081, 1339 and 1069, or 2281 and 2231 neurons for DNAJB14-FL or -short respectively. All experiments showed significant differences (differences were 37%, 22%, and 18%, *p* = 7.9e−7, 0.023 and 5.7e−3 for the three replicates, calculated using student *t*-test, two-sided, non-adjusted, see “Methods”). Non normalized data presented in Supplementary Fig. 10f. Source data are provided as a Source data file. **e** qPCR for 8 proteostasis-related genes in neurons co-infected with FUS-R521H-YFP or FUS-WT-YFP together with DNAJB14-short or DNAJB14-FL showed that, as a group, their expression level is downregulated in mutFUS expressing neurons in the presence of DNAJB14-short compared to the WT (*p* = 0.01 and *p* = 5.6e−4), while DNAJB14-FL co-expression restored their expression to be similar to that of the FUS-WT-YFP expressing neurons (*p* = 0.0038 for mutFUS DNAJB14-FL vs. DNAJB14-short, and non-significant for mutFUS DNAJB14-FL vs. both WT-FUS). *P*-values were calculated using two-sided *t*-test, non-adjusted.

We next sought to examine the functional effects of the two naturally occurring DNAJB14 isoforms in primary neurons. To this end, we infected rat neuronal cultures with an AAV2 viral construct expressing either FUS-WT-YFP or FUS-R521H-YFP. Six days after infection, we observed a substantial amount of FUS-R521H-YFP aggregation within live neurons (Fig. 7a, Supplementary Fig. 10a). The aggregation phenotype was different than that observed in HEK293T cells, namely, rather than a single large aggregate, neurons mostly exhibited several large cytoplasmic FUS-R521H-YFP aggregates along with multiple smaller FUS-R521H-YFP foci in the cytoplasm (Fig. 7a, Supplementary Movies 1, 2). The latter could also be seen, though less frequently, in neuronal projections (Supplementary Fig. 10b). Nevertheless, in contrast to FUS-WT-YFP expressing neurons (Fig. 7b, Supplementary Movie 3), neurons that showed FUS-R521H-YFP cytoplasmic aggregates were largely devoid of fluorescent signal in the area of the nucleus (Fig. 7a). We next tested the functional effects of co-expression of either DNAJB14-FL or DNAJB14-short on aggregate formation. Due to the complex phenotype of FUS-R521H-YFP aggregates, we subjected confocal microscopy images of live neurons to image analysis. We used a classifier trained to distinguish between nuclear fluorescent pattern (as in FUS-WT-YFP expressing neurons, Fig. 7b) and cells displaying the complex FUS-R521H-YFP aggregate phenotype (see “Methods”). The classifier had a low background error rate, as shown by its application to FUS-WT-YFP infected neurons images (Supplementary Fig. 10c, d, see “Methods”). We then quantified the percentage of aggregate-containing neurons in FUS-R521H-YFP neuronal cultures co-infected with either DNAJB14-FL or DNAJB14-short, six days after viral infection. In total, we analyzed 4504 and 4381 FUS-R521H-YFP expressing neurons co-infected with DNAJB14-FL or DNAJB14-short, respectively. Strikingly, we observed that DNAJB14-FL expressing neurons showed significantly less FUS-R521H-YFP aggregation compared to DNAJB14-short expressing neurons (Fig. 7c, d, Supplementary Fig. 10e, f). FUS-R521H-YFP neuronal cultures showed on average 26% less aggregate-containing cells when expressing DNAJB14-FL compared to DNAJB14-short (*p* = 7.9e−7, 0.023, 5.7e−3, *N* = 3, Fig. 7d, Supplementary Fig. 10f, DNAJB14-FL and DNAJB14-short were titer-matched, see “Methods”).

Finally, we asked if mutFUS-expressing neurons showed impaired proteostasis, and whether DNAJB14-FL expression could restore proteostasis in primary neurons. We used qPCR to examine the expression of 8 proteostasis-related mRNAs, which showed deteriorated expression upon mutFUS aggregation and restoration by DNAJB14-FL in HEK293T cells. As only a small proportion of the population is infected, the effects are diluted and are therefore expected to be much smaller compared to the sorted HEK293T cells. Our results showed that, as a group, these proteostasis mRNAs indeed demonstrated reduced expression in mutFUS neurons co-infected with DNAJB14-short compared to WT-FUS expressing neurons, while the co-infection of DNAJB14-FL restored their expression to be similar to that of WT-FUS expressing neurons (Fig. 7e).

Therefore, our data demonstrated that the screen for mutFUS aggregation modulators performed in HEK293T human cells was able to identify chaperone modifiers that exhibited the same capability in modulating mutFUS aggregation, and proteostasis restoration, in cultured primary neurons.

## Discussion

Here we identified a complex of DNAJB14-FL and DNAJB12-FL, which interact with HSP70 to significantly protect cells from mutFUS aggregation (Fig. 4i). Importantly, naturally occurring short isoforms of both DNAJB12 and DNAJB14, which could not form a complex or interact with HSP70, were unable to significantly rescue mutFUS aggregation. Conversely, DNAJB12-short showed HTT-polyQ aggregation protection, while DNAJB12-FL aggravated HTT-polyQ aggregation. Thus, our work revealed that naturally-occurring DNAJ isoforms show different functional diversification towards different types of aggregates.

Although WT FUS is predominantly nuclear, it also exhibits nucleocytoplasmic shuttling^40^. A class of ALS mutations in FUS, including the R521H examined here, leads to FUS aggregation outside of the nucleus (Figs. 1a, 7a). Notably, FUS-R521H showed diffused nuclear localization in 26–40% of the cells (based on image analysis, Supplementary Fig. 6b, see “Methods”), but aggregates were rarely nuclear (1.5–2.6% of the cells, based on image analysis, see “Methods”). DNAJB12-FL and DNAJB14-FL are both ER localized (Supplementary Fig. 6i). They were previously shown to have roles in ERAD on the one hand^36–38^, and form the DJANGO structures^34^, whose function is currently unknown, on the other hand. DNAJB12-short was ER localized (Supplementary Fig. 6i), and, therefore, its mere localization cannot explain the differential protection from HTT-polyQ aggregation observed compared to DNAJB12-FL. It is possible, as HTT-polyQ aggregation was linked to impaired ER homeostasis^41,42^, that the effect of DNAJB12-short is indirect, e.g., via improving ER homeostasis, an effect which remains to be examined. Conversely, DNAJB14-short was cytoplasmic, but although it showed a low degree of binding to FUS (Fig. 4d), it could not protect from mutFUS aggregation. As this isoform lacks all other domains, it is probable that it is deficient in its chaperoning capacity. Moreover, while DNAJB14-FL formed DJANGOs in about 10–14% of the cells (Supplementary Fig. 6m), the ability to form DJANGOs was correlated with the ability to rescue mutFUS aggregation for the naturally-occurring isoforms, as well as for all other artificial isoforms we generated. The nuclear import factor Kap-beta2 was shown to inhibit FUS-R521H aggregation in cells^43^, supporting the notion that keeping mutFUS nuclear prevents its aggregation. An intriguing hypothesis is that the DJANGO structures may participate in regulating the shuttling of mutFUS, thereby aiding its solubility in the nucleus, however, the potential involvement of DJANGOs in mutFUS aggregation modulation remains for future exploration.

We found that while the DNAJB14-FL-mediated protection from mutFUS aggregation was HSP70 dependent, rescue of HTT-polyQ by DNAJB12-short was not only HSP70 independent, but the DNAJB12-FL isoform that interacts with HSP70 aggravated HTT-polyQ aggregation (Fig. 2b). Interestingly, in the case of the previously identified HTT-polyQ aggregation modulators DNAJB6 and DNAJB8^15^, aggregation protection was shown to be, at least in part, HSP70 independent^15^. It therefore seems that HTT-polyQ aggregation protection can be performed by several DNAJ proteins in an HSP70-independant manner. In contrast, as we found here, mutFUS aggregation suppression required HSP70 in the context of the DNAJB14–DNAJB12 complex. These findings highlight different chaperoning requirements from these different types of pathological aggregates. Indeed, the process of aggregation is different for these two aggregate types; while HTT-polyQ generates amyloid fibrils through a more classical process of oligomerization^7,8^, mutFUS aggregation involves the process of aberrant LLPS^9^, and our data indicate that DNAJB14-FL increased the mobility of mutFUS aggregates (Fig. 5), thereby affecting the properties of mutFUS LLPS.

Our RNA-seq experiments found that different Hsp70s were induced in response to HTT-polyQ aggregation (Fig. 1d, Supplementary Fig. 2d), including marked induction in Hspa6 levels, a chaperone which aggravated the HTT-polyQ aggregation phenotype (Fig. 2b). In contrast, Hsp70s levels were reduced in response to mutFUS aggregation (Fig. 1d, Supplementary Fig. 2d, e), whereas their association with DNAJB14-FL was critical for aggregation protection (Fig. 4b). Interestingly, in addition to directly binding FUS (Fig. 4d) and increasing mutFUS aggregate mobility (Fig. 5), DNAJB14-FL expression had a profound effect on gene expression in mutFUS aggregate-containing cells, and on proteostasis gene expression in particular (Fig. 6, Supplementary Fig. 9). DNAJB14-FL expression restored deteriorated proteostasis, and reverted the cellular gene expression state of mutFUS aggregate-containing cells to a near WT state, an effect that was also recapitulated in primary neurons expressing mutFUS (Fig. 7e). This represented a fine-tuned, apparently well-suited response to address the challenges of mutFUS aggregated cells. These evidence further highlight the dissonance between the chaperoning requirements of the two different aggregate types, and the maladaptive cellular response elicited in response to the presence of each of them.

**Fig. 8:**
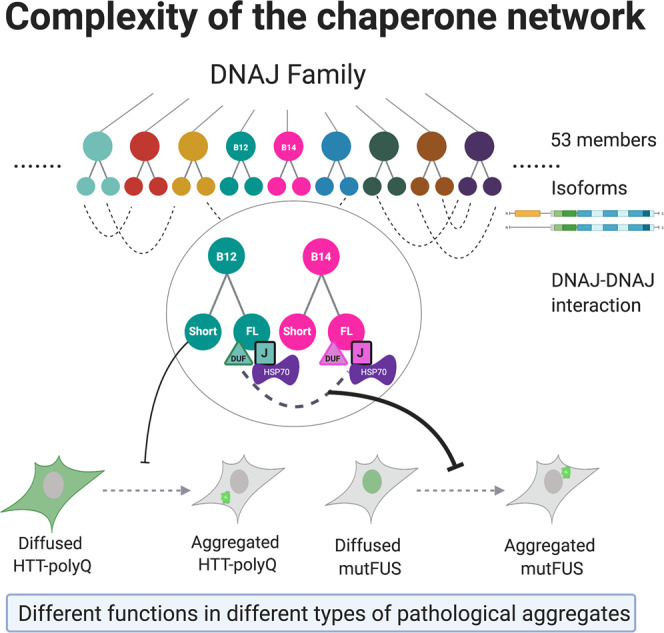
Chaperone network complexity conferred by DNAJ isoforms and their differential functions. Naturally-occurring DNAJ isoforms and their interactions show differential functions with respect to different aggregate types, revealing inter-family functional diversification, thereby increasing the complexity of the chaperone network. Created with BioRender.com.

The chaperone network in general, and the HSP40 (DNAJ) family in particular, has undergone a major expansion in evolution, and the human genome contains 53 different DNAJ proteins^13^. Our work reveals that the interplay between naturally-occurring isoforms of DNAJs further increases the complexity of the chaperone network, by generating functional diversification towards different types of pathological aggregates (Fig. 8). While in adult human tissues, DNAJB14-short and DNAJB12-short are basally lowly expressed (Supplementary Fig. 11), their expression in response to environmental or physiological perturbations remains to be explored. Modulation of protein homeostasis in general, and of protein aggregation in particular, can potentially be performed by isoform switching of DNAJs, in yet to be identified conditions. This further increases the complexity of the chaperone network as we view it today (Fig. 8), and in addition to overexpression of specific chaperone isoforms as shown here, has the potential to further serve as a point for therapeutic intervention.

## Methods

### Cloning and plasmid preparation

HTT-134Q-GFP and HTT-17Q-GFP containing plasmid were a gift from Prof. Noam Ziv, and were cloned into the pTREX backbone. FUS-WT-YFP, FUS-R521H-YFP, and FUS-R518K-YFP constructs^25^ were purchased from Addgene, and then cloned into the pTREX backbone using PCR and gateway cloning. Subsequently, the Quikchange 2 Site-Directed Mutagenesis Kit (Agilent Technologies 210518) was used to introduce the additional mutations: p.R521C, p.R495X, p.R495EfsX527 into the pTREX-FUS-WT-YFP backbone. For p.R495EfsX527, a frameshift of one base was generated in the linker between FUS and YFP to keep the C terminal YFP protein in-frame. For p.R495X mutation, nucleotides from position 1483 in FUS and up to the linker were deleted to keep the C terminal marker expressed.

C-terminal FLAG-tagged library of 66 chaperones was prepared from the ORFeome library (purchased from GE Healthcare), and transferred from pDONR223 into pcDNA3.1-ccdb-FLAG-V5 using gateway cloning. Plasmids expressing mOrange-tagged proteins were prepared using gateway cloning into a pcDNA3.1-ccdb-mOrange backbone, which was generates in house. In addition, four isoforms of DNAJB14 were generated (all primers are listed in Supplementary Table 2): DNJB14-short lacking the J-domain, TM and DUF domain (Fig. 4a) was generate from the full-length isoform using PCR and gateway cloning; two mutations in the HPD domain Histidine (H) to Glutamine (Q) in position 136 of the protein, introduced using the Quikchange 2 Site-Directed Mutagenesis Kit; DNAJB14-ΔDUF and DNAJB14-Δshort truncations were obtained using PCR from the full-length isoform and gateway cloning (see Fig. 4a). The pAAV-FUS-R521H-YFP, pAAV-FUS-WT-YFP, pAAV-DNAJB14-FL and pAAV-DNAJB14-short plasmids were constructed using Gateway cloning into pAAV-CAG-dest. pAAV-CAG-dest was a gift from Prof. Yitzhak Kehat. An ER marker plasmid, mCherry-ER-3, containing the CALR signal peptide region fused to mCherry, was a gift from Michael Davidson (Addgene plasmid #55041). DNAJB14-FL and DNAJB14-short were also generated in pcDNA3.1 using gateway cloning (for Supplementary Fig. 6a). DNAJB14 and DNAJB12 isoforms accession numbers: DNAJB14-FL—ENST00000442697 (NM_001031723/uc003hvl), DNAJB14 short—uc003hvm.4 (similar to ENST00000469942.1), DNAJB12-FL—ENST00000394903 (NM_017626), DNAJB12-short—ENST00000461919 (KJ902687).

### Cell culture and transfection

HEK293T cells were grown in standard DMEM supplemented with 10% FBS, 1% Penicillin-Streptomycin in 37 °C, 5% CO_2_ incubator. For the FUS screen transfections, HEK293T cells (3e5 cells per well) were seeded on 6well plates and transfected using PEI transfection reagent (Thermo Fisher Scientific) one day after seeding, according to the manufacturer instruction, with 1250 ngr of FUS DNA and 1250 ngr of chaperone DNA. For HTT screen transfections, cells were seeded at 25,000 cells per well on 96 well plates one day before transfection, and transfected using PEI with 200 ngr of HTT DNA and 200 ngr of chaperone DNA. Cells were assayed 48 h following the transfection.

### PulSA method

At 48 h after transfection, cells were trypsinized, resuspended in media with serum, placed on ice, and DAPI regent was added (5 µM) in order to assay cell viability. Cells were then taken to Flow cytometry in order to differentiate between subpopulations with diffused cellular fluorescence and those with protein aggregates, as in Ramdzan et al.^27^, and as illustrated in Fig. 2a. To obtain Aggregation modulation scores, cells of appropriate size were considered during the FACS analysis, and non-viable cells (positive for DAPI) were excluded. We then quantified the percentage of cells that showed a fluorescent aggregate pattern (AGG+), and compared these percentages to cells co-transfected with DsRed as a control. Aggregation modulation scores for each chaperone were calculated as Log2(%AGG+ (chaperone)/%AGG+ (DsRed)). Each experiment was performed in 4 independent biological replicates for 66 chaperones. Confidence intervals (95%, appear as dashed lines in Figs. 2b, 3a, etc.) were calculated according to the variations between different DsRed co-transfected cells replicates, such that they represent twice the standard deviation of Aggregation modulation scores calculated between different replicates of DsRed performed on the same day. Chaperones or samples were called as significant modulators if their average Aggregation modulation scores + (or −) SEM was below (or above) the 95% CI. Specifically, and empirical *p*-value was indicated such that *p* < 0.05 if these values were below the 95% CI, and *p* < 0.003 if these values were below the 99.7% CI (i.e., 3 times STD). In order to find an appropriate baseline control for the screen, we tested aggregation rates in cells co-transfected with each of two unrelated proteins: Renilla luciferase, or DsRed, while also comparing to cells transfected with the aggregated protein alone. As co-expression of DsRed gave lower levels of basal aggregation rates (Supplementary Figs. 3a, b, 5a), we chose the more restrictive baseline and used DsRed as a control.

### Chaperone overexpression levels

The degree of chaperone overexpression was measured using a sandwich ELISA assay. FLAG-tagged chaperones were co-transfected into HEK293T cells together with either HTT-134Q-GFP or FUS-R521H-YFP, and 48 h later cells were lysed (Lysis and wash buffer containing 50 mM HEPES pH 7.9, 150 mM NaCl, 2 mM EDTA, 0.5% Triton x-100, 5% Glycerol, with addition of protease inhibitor cocktail) and lysates were transferred into 384well plates pre-coated with anti-FLAG antibody (Sigma, F1804), for FLAG pulldown for 3 h at 4° with slow tilting. Then, plates were washed 7 times with lysis and wash buffer and FLAG ELISA was performed with a different anti-FLAG antibody (abcam ab1238), using a Tecan M200Pro plate reader.

### RNA-seq and data analyses

For the RNA-seq presented in Fig. 1, cells were transfected with either HTT-134Q-GFP, HTT-17Q-GFP, FUS-R521H-YFP, FUS-R518K-YFP or FUS-WT-YFP in 10 cm plates. At 48 h after transfection, cells were trypsinized from plates into ice-cold standard DMEM supplemented with 10% FBS, with 100 μg/ml CHX. FACS and sorting were performed on a BD FACSAria III sorter. Sorting cells into aggregate (AGG+) and diffused (AGG−) expressing populations by the PulsA method was performed as described above. Sorting time was limited to 12 min to minimize toxic effects of exposure to cycloheximide. Tubes containing sorted cells were centrifuged for 7 min at 4 °C at 300 RCF, and the cell pellets were flash-frozen in liquid nitrogen. For the RNA-seq experiment in Fig. 6, cells were transfected with either FUS-R521H-YFP or FUS-WT-YFP, together with either DNAJB14-FL, DNAJB14-short, or DsRed in 6 well plates. At 48 h after transfection, cells were lifted from plates into standard DMEM supplemented with 10% FBS. FACS and sorting were performed on a BD FACSAria III sorter. Sorting cells into AGG+ and AGG- populations was performed as above. RNA was further extracted from cells using RNeasy Mini kit (Qiagen, #74104).

Transcriptome library preparation was performed as in Sabath et al.^44^. For RNA-seq analysis, mapping of raw reads to whole length transcriptome sequences was done using the STAR^45^ aligner with the following parameters:--outFilterMatchNminOverLread 0.6.

Transcriptome sequences were taken from RefSeq genome annotation of hg19 human genome. UCSC RefSeq hg19 table was downloaded from the UCSC genome browser website, using UCSC Table Browser data retrieval tool^46^. After alignment, quantification was done using the RSEM^47^ software package.

Expressed genes were defined as genes that had a TPM value larger than or equal to the cutoff value (6 for FUS and 10 for HTT in the RNA-seq from Figs. 1 and 2 for the RNA-seq from Fig. 6) in at least one sample in the datasets. Differential expression analysis of transcriptome data was performed on raw read counts data acquired by RSEM^47^, using DESeq2 R package^48^, and a set of significantly differential genes was defined as all expressed genes that were significantly changed in at least one comparison, with a False Discovery Rate (FDR) value smaller than 0.05. For FUS and FUS co-transfection experiments, an additional LFC cutoff of 1 was added. For Fig. 1, hierarchical clustering was performed using LFC calculated by running DESeq2 on both mutants combined, in order to give equal weight in clustering for FUS and HTT data, with correlation used as a distance metric. These LFC values were used in all cumulative distribution function (CDF) plots. Clustering results were visualized in a heatmap using LFC values which were calculated separately for the FUS-R518H and FUS-R521K samples (Fig. 1).

Gene Ontology Term enrichment analysis of clusters was performed using DAVID 6.8 online service^49^, through the RDAVIDWebService^50^ R package. All genes defined as expressed were used as the background group. To calculate the statistical significance of change in gene expression of specific gene groups of interest compared to the background, Student’s *t*-test was performed on the set of LFCs of the gene group, compared to the LFCs of the background. Group of Chaperones was defined as in Sabath et al.^44^. “Response to unfolded protein” and “Protein refolding” gene ontology groups were downloaded from Gene Ontology Resource^51^. HSP70, HSP60, HSP90 and ER chaperone family groups were taken from Brehme et al.^52^. “HSP70 only” group was defined as the HSP70 family group from Brehme et al., excluding nucleotide exchange factors (NEFs).

### Dose-dependent aggregation modulation scores

To check the effect of different doses of chaperones on aggregation modulation, cells were transfected one day after seeding (3e5 cells per well in a 6well plate) in three doses: 2000 ngr of FUS DNA plus 500 ngr of chaperone DNA, 1250 ngr of FUS DNA plus 1250 ngr of chaperone DNA, and 700 ngr of FUS DNA plus 1800 ngr of chaperone DNA (Fig. 3b). Cells were assayed 48 h following transfection by PulSA as above. Each combination was compared to a respective combination of FUS DNA plus DsRed DNA to obtain the Aggregation modulation score.

### Immunofluorescence staining

Cells were cultured on cover-slips and transfected as above. Two days post transfection, cells were fixed in 4% paraformaldehyde in PBS, permeabilized with 0.5% Triton-x100 in IF buffer (5% FCS, 2% BSA in PBS), then immunostained by primary anti-FLAG antibody (Sigma-Aldrich, F1804) followed by secondary AlexaFluor 647 Donkey Anti-Mouse (Jackson) antibody to image FLAG-tagged chaperones. The far-red secondary antibody was used to eliminate fluorescence overlap between the YFP/GFP-tagged aggregates and the chaperones. The cells were additionally stained with DAPI to mark nuclei.

### Image analysis

A Fiji script was automatically run on all images identifying cells containing both nuclear marker (DAPI) and FLAG tagged chaperone staining (as above). This generated a binary image that contained cells above a fixed threshold for each channel. After size filtering and signal thresholding cells were counted.

DJANGO structure identification was done using the Trainable Weka Segmentation plugin, followed by summation of the signal and thresholding.

Aggregates were detected using the Trainable Weka Segmentation plugin, and so were cells with diffused FUS-R521H-YFP fluorescence. Aggregate localization was determined using summation of the identified aggregate signals either inside the nucleus (which were modeled by the DAPI channel) or outside (using a larger definition of a fixed area around the nucleus, and subtraction of any aggregates signal that was identified within this area from the total aggregate area calculated per cell).

### Live cell imaging

Cells were cultured as mentioned above and co-transfected with FUS-R521H-YFP and DNAJB14-FL or DNAJB14-short, respectively. At 24 h post transfection, plates were inserted to temperature and CO2 controlled (37 °C, 5% CO_2_) environment in a Zeiss Cell Discoverer 7 automated boxed microscope. Live imaging was performed with a ×20 0.95 NA dry plan apochromat objective for a total time of 54 h or more, each condition included 3 fields of view and each view was imaged as a 86.45 mm × 48.07 mm 2 × 2 tile image.

### Co-immunoprecipitation

For co-immunoprecipitation (co-IP) of FLAG-tagged chaperones, lysis buffer was prepared as follows: 50 mM HEPES pH 7.9, 0.5% Triton X-100, 5% glycerol, 150 mM NaCl and 2 mM EDTA and Protease inhibitors (Sigma-Aldrich P5726). Wash buffer was prepared as follows: 50 mM HEPES pH 7.9, 1% Triton X-100, 5% glycerol, 150 mM NaCl and 2 mM EDTA. Cells were transfected as above. At 48 h post transfection, cells were placed on ice, washed with ice-cold PBS, lysis buffer was added and cells were collected by scraping. Lysates were centrifuged (8200 g for 10 min at 4 °C), and supernatant was transferred to new tubes. Samples were IP-ed using 10 µl of EZview Red anti-FLAG M2 Affinity Gel beads (Sigma-Aldrich, F2426), which were added to a sample volume containing equal number of 260 nm OD units. Additional co-IP buffer was added to reach a 600 µl volume for each sample and samples with beads were slowly rotated 3 h at 4 °C. Beads were then washed 7 times with 1 ml of wash buffer. Following the last wash, the beads were resuspended in equal volumes of wash buffer, protein sample buffer was added, and the samples were boiled at 100 °C for 5 min. Equal volumes were then run on an 8% or a 12.5% gel, and immunoblotted using anti-FLAG (Sigma-Aldrich, F1804), anti-HSC70/HSP70 (Enzo Life Sciences, N27F34), or anti-RFP (abcam, ab34771) antibody, which was used to detect mOrange tagged proteins. For Fig. 4f, Supplementary Figs. 6p, 8a, anti-FLAG: the membrane containing DNAJB14-FL– used with secondary antibody LC fragment specific and membrane containing DNAJB14-short and ΔDUF—used with secondary antibody FC fragment specific. Immunoblots densitometry was quantified using Fiji, where interactor levels (HSP70 or proteins tagged with mOrange) were normalized to the pulled-down protein levels (FLAG).

For co-immunoprecipitation (co-IP) of FUS, lysis was performed using RIPA buffer (50 mM TRIS 0.5% NaDOC, 1% NP40, 150 mM NaCl and 0.5 mM DTT and Protease inhibitors). Cells transfected with FUS-R521H-YFP or FUS-WT-YFP, together with either DNAJB14-FL, DNAJB14-short or DNAJB14-Δshort. At 48 h post transfection, cells were placed on ice, washed with ice-cold PBS, lysis buffer was added and cells were collected by scraping. Lysates were centrifuged (8200 × *g* for 10 min at 4 °C), and supernatant was transferred to new tubes. Samples were IP-ed using 25 µl of Dynabeads Protein A (Thermo Fisher, 10001D), which were crosslinked to an anti-GFP (MBL, #598) antibody using conjugation buffer and BS3 according to manufacturer’s instructions. Samples with beads were slowly rotated overnight at 4 °C. Beads were then washed 4 times with 600 µl of RIPA buffer. Following the last wash, proteins were eluted from the beads using glycine 50 mM, ph = 2.5, and then protein sample buffer with DTT was added and the beads were boiled at 100 °C for 5 min. Equal volumes were then run on a 15% gel, and immunoblotted using anti-FLAG antibody. Immunoblots densitometry was quantified using Fiji. Molecular size markers and membrane full scans are provided either in the figures or in the Source data file.

### Heat shock-responsive inducible promoter assay

The human HSPA7 promoter was cloned from HEK293T genomic DNA using PCR (see primers in Supplementary Table 2) into a pcDNA3.1-GFP plasmid, replacing the CMV promoter using Gibson cloning (Gibson Assembly Master Mix, NEB #E2611) according to the manufacturer’s instructions. HEK293T cells (25,000) were seeded in 96 well plate. At 24 h after seeding, cells were co-transfected with the HSPA7 promoter-GFP plasmid together with each of the chaperones, or with an empty pcDNA3.1 plasmid, using 200 ng DNA each. At 24 h after transfection, 0.7 mΜ MG132 was added as a positive control to wells transfected with the HSPA7 promoter-GFP+ pcDNA3.1 plasmids. After 4 h, the plate was placed in an M200 Tecan plate reader for 24 h, with fluorescence reading every 2 h. To analyze the degree of HSR induction for each chaperone, first baseline fluorescence (as measured in the wells co-expressing an empty pcDNA3.1 plasmid) was subtracted from all measurements in the experiments in each timepoint. Then, the fluorescence levels in each experiment were normalized as percentage from the wells co-expressing an empty pcDNA3.1 plasmid treated with MG132 in each timepoint. Finally, a mean across all replicate experiments (*N* = 3) was calculated along the 12h-24h timepoints. These scores showed no correlation with Aggregation modulation scores for neither mutFUS (Supplementary Fig. 9j) nor HTT-polyQ (Supplementary Fig. 9k).

### qPCR in HEK293T cells

Cell were seeded in 6 well plates at a density of 400 k/well. One day after seeding, cells were transfected with different chaperones: DNAJB14-FL, HSPA9, DNAJB8, DNAJC11, DNAJB2, DNAJC5B, DNAJC7, NACA2. At 48 h after transfection RNA was extracted using the quick RNA miniprep kit (Zymo, R1504) and cDNA was generated using MMLV reverse transcriptase (Promega M170A). qPCR was then performed for the Hspa1a and normalized to HPRT) (see primers in Supplementary Table 2).

### FRAP analysis

To study the mobility of FUS-R521H-YFP aggregates, we have transfected HEK293T cells with FUS-R521H-YFP together with either DNAJB14-FL-mOrange or DNAJB14-short-mOrange as above. Two days later, we monitored YFP fluorescence by irradiation with 488 nm excitation at low intensity (0.2% laser power) with a ×20 water immersion objective (LSM-900, Zeiss, Germany). We imaged 50 mm thick optical slices (pinhole: 2.67 AU) of standard image size (512 × 512 pixel) at 2.7 digital zoom with at the rate of 930 ms/frame (1.5 ms/pixel). We defined regions within individual cells for subsequent photoablation (i.e., photobleaching) by 100% 488 nm irradiation. We initially monitored aggregates mobility (prior to photobleaching) for a duration of about 20-30 images, followed by rapid photoablation (100% 488 nm laser, at 350 iterations/region, lasting <0.5 s, five times; depending on size of ROI within each cell), then continued imaging for another 280 s or more. We ensured a high percentage of bleaching (<90%), and calibrates the bleaching conditions such that neighboring cells were unaffected.

For each cell, *t* = 0 was assigned to be the time of photobleaching. We normalized the raw data relative to baseline fluorescence before bleaching for each cell: I_t,norm_ = (I_t,raw_)/(I_−1to-27_ (median of 27 images)). This normalization removes variations in expression levels but also of the laser intensity used for bleaching. We repeated the analysis over three independent biological replicate transfections for a total of 156 and 150 cells for DNAJB14-FL and DNAJB14-short co-expressing cells respectively. Data are presented as mean ± SEM.

### Small interfering RNA

Cells were seeded at 150,000 cells per 6well plate one day before transfection. siRNA transfections were performed using RNAiMAX lipofactamine reagent (Thermo Fisher Scientific), according to the manufacturer’s protocol, with siRNA concentration of 15 nM. One day after transfection of the siDNAJB12 or siControl (Dharmacon Smartpool, 001810-10-05 or 020585-01-0005), cells were co-transfected with 1250ngr of FUS-R521H and 1250ngr of DNAJB14-FL using PEI. Cells were analyzed 48 h later.

### Virus production

All viruses were packaged with the AAV-PHP.B capsid using an optimized protocol by Challis et al.^53^ for producing AAV viruses from HEK293T cells grown on six 10 cm plates. All viruses were then tittered using qPCR in the same assay and dosage consistency was kept between pAAV-FUS-R521H-YFP and pAAV-FUS-WT-YFP (infections made at the same titer), and between pAAV-DNAJB14-FL, and pAAV-DNAJB14-short (infections made at the same titer).

### Neuronal cultures and infections

Isolation and culturing of rat primary hippocampal neurons were done as previously described^54^, and received an ethics approval from the Technion, Israel (Ethics certificate No. IL-130-09-17). Briefly, P0 neonates, from timed-pregnant white coat female Sprague Dawley female rats (Charles River; SAS SD, strain code 400), were euthanized and their hippocampi dissected and dissociated. Then, 150 K cells were plated in 24-well flat bottom plates on coverslips (12 mm, thickness ~0.1 mm) coated with Poly-D-Lysine (Sigma-Aldrich P7886-50MGP) in Minimum essential medium (MEM), glutamine-free based Neuronal growth medium (5% fetal bovine serum, 2% B27, 1% Glutamax (100×, Invitrogen), 2% 1 mol/L d-glucose, 0.1% μL serum extender (BD Biosciences, Cat. No. 355006)). Cultures were incubated at 37 °C and 5% CO_2_. At 5 days after plating, media was changed and supplemented with cytosine arabinosine (Ara-C, Sigma, C6645) at a final concentration of 4 μM, to reduce the growth of glia in the cultures. Cultures were infected the same day with pAAV-FUS-R521H-YFP or pAAV-FUS-WT-YFP (at the same titer), together with either pAAV-DNAJB14-FL or pAAV-DNAJB14-short (at the same titer). After that media was changed every 2 days, and cells were imaged 6 days after infection.

### Neurons image analysis

For microscopy-analysis imaging, co-infection of pAAV-FUS-R521H-YFP or pAAV-FUS-WT-YFP with equal dosages of either pAAV-DNAJB14-FL or pAAV-DNAJB14-short virions occurred at the 5th day post primary cell culture isolation, imaging took place at the 6th post infection.

Images were acquired with a Zeiss LSM 880 confocal microscope using water dipping Plan-Apochromat ×20 objective, (working distance, 1.8 mm). The acquired images were processed and analyzed using a Fiji script. In short, out of each confocal microscopy image, 15 slices were successively chosen along the z-axis and stacked to form one image. An equal number of regions of interest (651 × 651 μm in size) were randomly chosen as input for the script. Then, total cell numbers per processed image were identified based on intensity threshold (>240), and images were matched between DNAJB14-FL and DNAJB14-short sets to have approximately the same number of cells. Subsequently, separation of cells was performed based on watershed processing, cell filtering was based on size (>50 μm2), and cellular aggregate detection was performed using Trainable Weka Segmentation plugin, summing aggregate area per cell, where a total area >5 μm were considered aggregates. Finally, a Python script was used to extract output excel files.

Each biological replicate, which included either pAAV-FUS-R521H-YFP or pAAV-FUS-WT-YFP (at equal titers), each co-infected together with pAAV-DNAJB14-FL or pAAV-DNAJB14-short (at equal titers) was imaged to obtain thousands of neurons. For pAAV-FUS-R521H-YFP we obtained 12, 24 and 37 images from each of DNAJB14-FL or DNAJB14-short samples (containing a total of 884 and 1081, 1339 and 1069, 2281 and 2231 neurons for DNAJB14-FL and -short respectively). For pAAV-FUS-WT-YFP we obtained 16 images for each of DNAJB14-FL and DNAJB14-short samples (containing 741 and 883, 659 and 689, 1131 and 1208 neurons for DNAJB14-FL and -short respectively). These images were subjected to image analysis as described above. The number of aggregate-containing cells was divided by the total number of fluorescent cells detected by the classifier to obtain a fraction of aggregate-containing neuron for each image. Then, average and std error for the fraction of aggregate-containing neurons was calculated for DNAJB14-FL and -short in each experiment, and a two-tailed t-test p-value was calculated for the difference between the fraction of aggregate-containing neurons calculated for DNAJB14-FL and -short in each experiment. The image analysis classifier had a low background error rate, demonstrated by its application to images taken from FUS-WT-YFP expressing neurons, and no consistent difference was observed between DNAJB14-FL and DNAJB14-short FUS-WT-YFP co-expressing neurons. Overall 1.3% and 2.3% aggregation rate was identified for DNAJB14-FL and DNAJB14-short respectively in FUS-WT-YFP expressing neurons images (Supplementary Fig. 10c).

### RNA extraction and qPCR in primary neurons

Hippocampal neurons were infected as above. RNA was extracted at day 5 post infection using a total RNA purification Micro kit (NORGEN 35300). qPCR was performed for 8 proteostasis-related mRNAs: DNAJC2, DNAJC3, HSP90AB1, HSPA9, HSPA5, BAG4, DNAJB11 and PSMD1, and results were normalized to HPRT (see primers in Supplementary Table 2). This was performed in a total of *N* = 5–7 replicates. Subsequently, the average of each mRNA expression levels across all replicates was further mean-normalized across all samples for each gene separately, and presented in a CDF plot.

### Reporting summary

Further information on research design is available in the Nature Research Reporting Summary linked to this article.

## Supplementary information

Supplementary Information

Description of Additional Supplementary Files

Supplementary Data 1

Supplementary Movie 1

Supplementary Movie 2

Supplementary Movie 3

Reporting Summary

## Data Availability

The datasets generated during the current study are included in this published article and its Supplementary information files. The raw FACS data files used in this study to generate Aggregation Modulation Scores are available from the corresponding author upon request, and numeric data are available in the Source data file. The RNA-seq data generated in this study have been deposited in the GEO database under accession number GSE165317. Source data are provided with this paper.

## Source data

Source data
