# Supplementary material for: Differential roles for DNAJ isoforms in HTT-polyQ and FUS aggregation modulation revealed by chaperone screens
Source: Nat Commun. 2022 Jan 26;13:516. doi: 10.1038/s41467-022-27982-w (PMC8792056; doi:10.1038/s41467-022-27982-w)
Supplement: Supplementary file 1 — Supplementary Information [file 41467_2022_27982_MOESM1_ESM.pdf]

Supplementary Information

Differential roles for DNAJ isoforms in HTT-polyQ and FUS aggregation modulation revealed by chaperone screens

Kinneret Rozales[#,1], Amal Younis[#,1], Naseeb Saida[1], Anatoly Meller[1], Hodaya Goldman[1], Lior Kellerman[1], Ronit Heinrich[2], Shai Berlin[2], and Reut Shalgi[*,1]

[1]Department of Biochemistry, Rappaport Faculty of Medicine, Technion–Israel Institute of Technology, Haifa 31096, Israel

[2]Department of Neuroscience, Rappaport Faculty of Medicine, Technion–Israel Institute of Technology, Haifa 31096, Israel

[#]These authors equally contributed to the work

* Corresponding author: reutshalgi@technion.ac.il

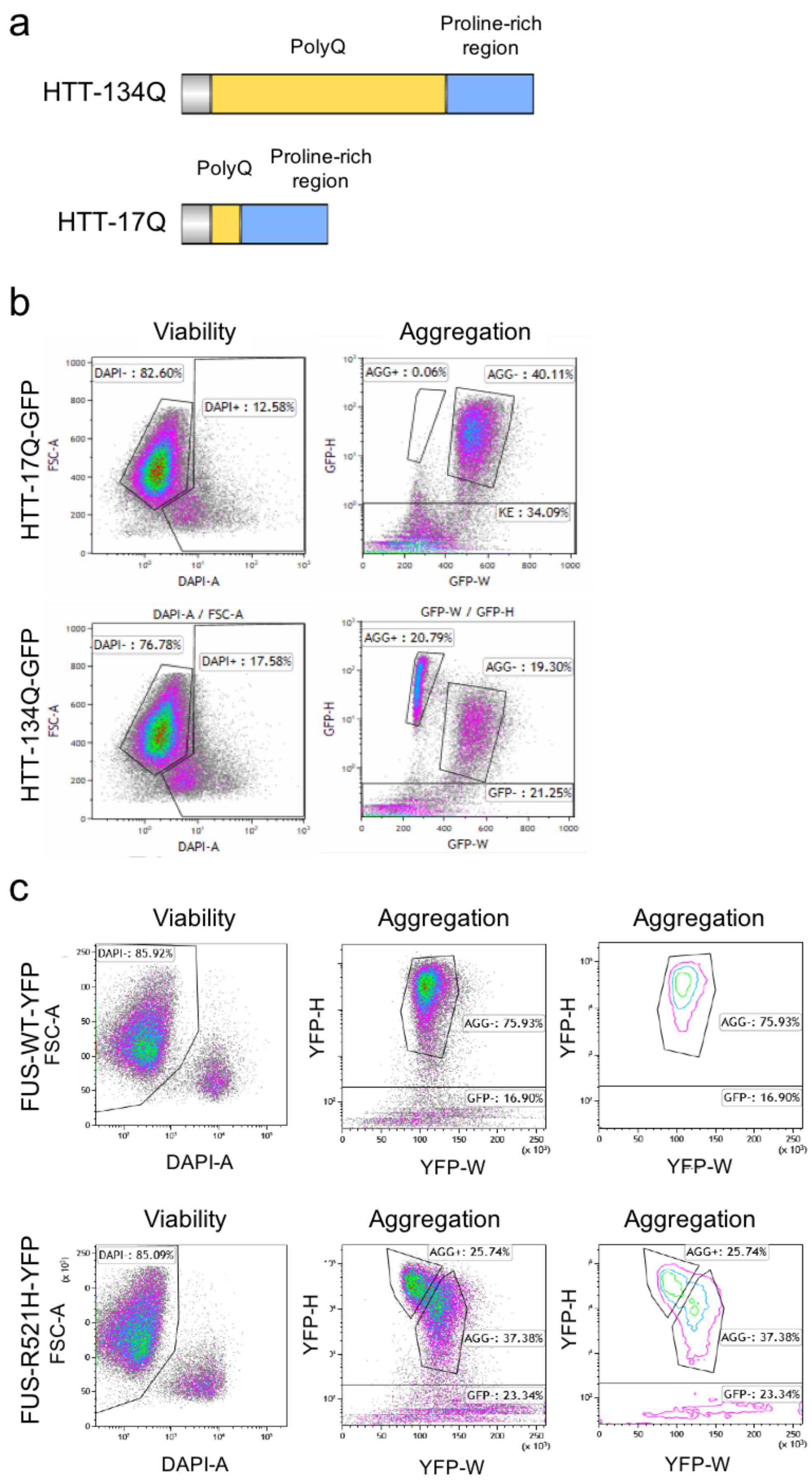

a
HTT-134Q
PolyQ
Proline-rich region
HTT-17Q
PolyQ
Proline-rich region
b
Viability
Aggregation
HTT-17Q-GFP
DAPI- : 82.60%
DAPI+ : 12.58%
FSC-A
DAPI-A
AGG+ : 0.06%
AGG- : 40.11%
KE : 34.09%
GFP-H
GFP-W
HTT-134Q-GFP
DAPI-A / FSC-A
DAPI- : 76.78%
DAPI+ : 17.58%
GFP-W / GFP-H
AGG+ : 20.79%
AGG- : 19.30%
GFP- : 21.25%
c
Viability
Aggregation
Aggregation
FUS-WT-YFP
FSC-A
DAPI-: 85.92%
DAPI-A
YFP-H
AGG-: 75.93%
GFP-: 16.90%
YFP-W
FUS-R521H-YFP
DAPI-: 85.09%
AGG+: 25.74%
AGG-: 37.38%
GFP-: 23.34%

Supplementary Figure 1: HTT-134Q and mutFUS aggregation analyzed by PuLSA flow cytometry.

(a) Schematic illustration of the CAG triplet repeat expansion, encoding polyglutamine (polyQ, yellow), within exon 1 of the HTT gene. Proline-rich region is also present in the plasmid (blue). (b) Cells were transfected with HTTQ17-GFP or HTT134Q-GFP and analyzed by PuLSA[1] 48 hours later. DAPI exclusion (left) was used to quantify and filter only viable cells. Diffused expression of HTT17Q-GFP protein (top right) shows a single fluorescent cell population, while expression of aggregating HTT134Q-GFP (bottom right) gives rise to a new distinct subpopulation, with a smaller fluorescence peak width and overall higher intensity (gate AGG+). (c) Cells were transfected with FUS-R521H-YFP or FUS-WT-YFP and analyzed by FACS 48 hours later. DAPI exclusion was used to exclude and filter for viable cells only. Diffused expression of FUS-WT-YFP protein shows a single population, while expression of aggregating FUS-R521H-YFP gives rise to a new subpopulation, with a smaller fluorescence peak width and overall higher intensity. Contour plots (on the right) demonstrate the two subpopulations in FUS-R521H-YFP expressing cells (bottom) vs. a single population with FUS-WT-YFP expressing cells.

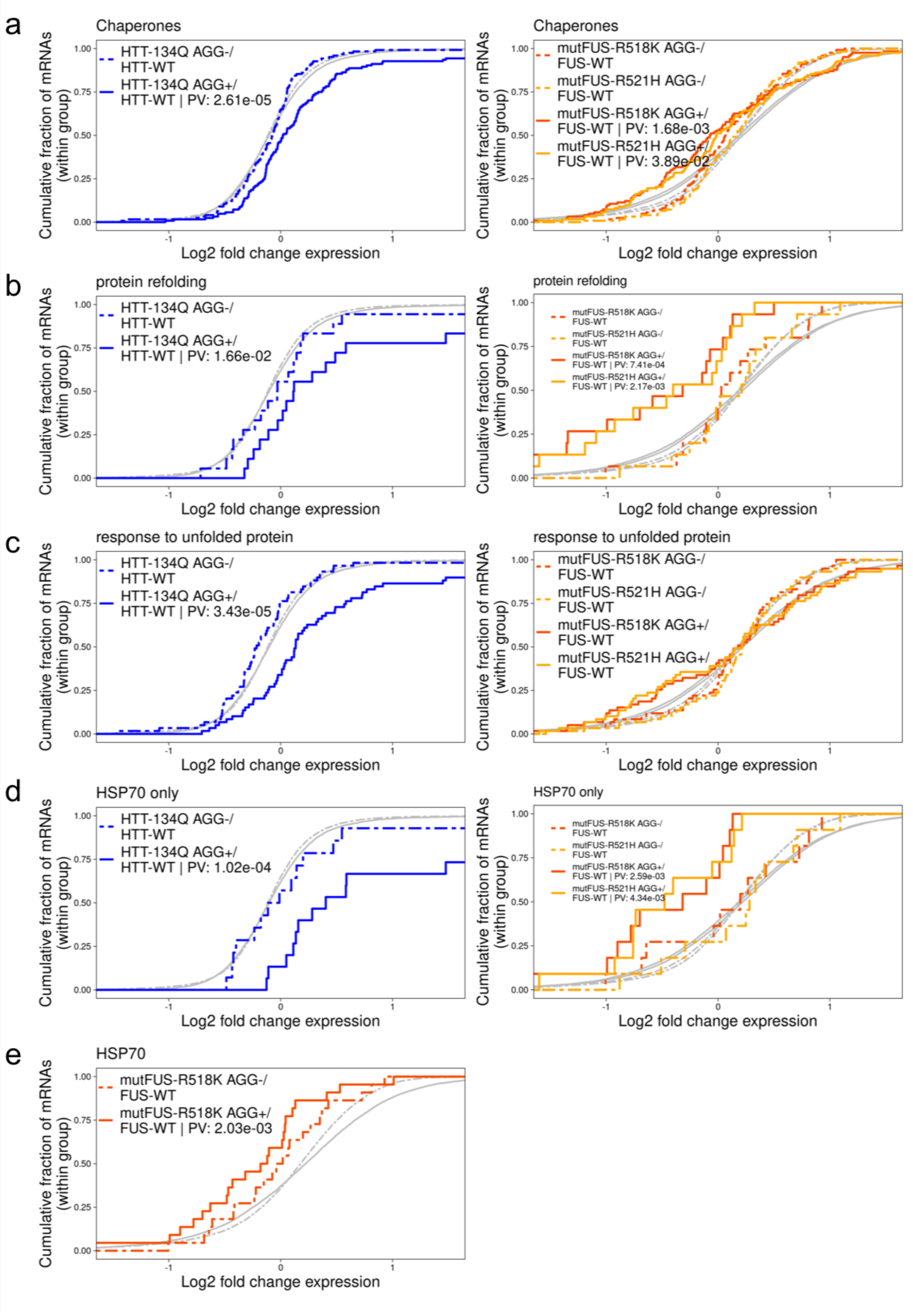

Supplementary Figure 2: Stress response and chaperones are induced in response to HTT-polyQ aggregation, and repressed in mutFUS aggregate-containing cells.

Cumulative distribution function (CDF, y-axis) of Log2 Fold Change (LFC) values, as in Fig. 1d, for defined groups of mRNAs. Expression was significantly induced in cells containing HTT-134Q aggregates (blue), but not in mutFUS expressing cells (yellow – FUR-R521H-YFP and orange – FUR-R518K-YFP). (a) Chaperones, as defined in Sabath et al.[2]. The Gene Ontology categories "protein refolding" (b) and

“response to unfolded proteins” (c), HSP70 family chaperones (excluding NEFs), as defined in Brehme et al.[3] (d) and HSP70 for FUS-R518K-YFP as in Fig. 1d (e). In all cases, groups were induced in response to HTT-polyQ aggregation, but not in response to aggregation of mutFUS. Background (grey) – defined as all expressed genes. t-test p-values are shown in the legend, for the difference between the LFC distribution of the group of interest compared to the respective LFC distribution of the background set, only when significant.

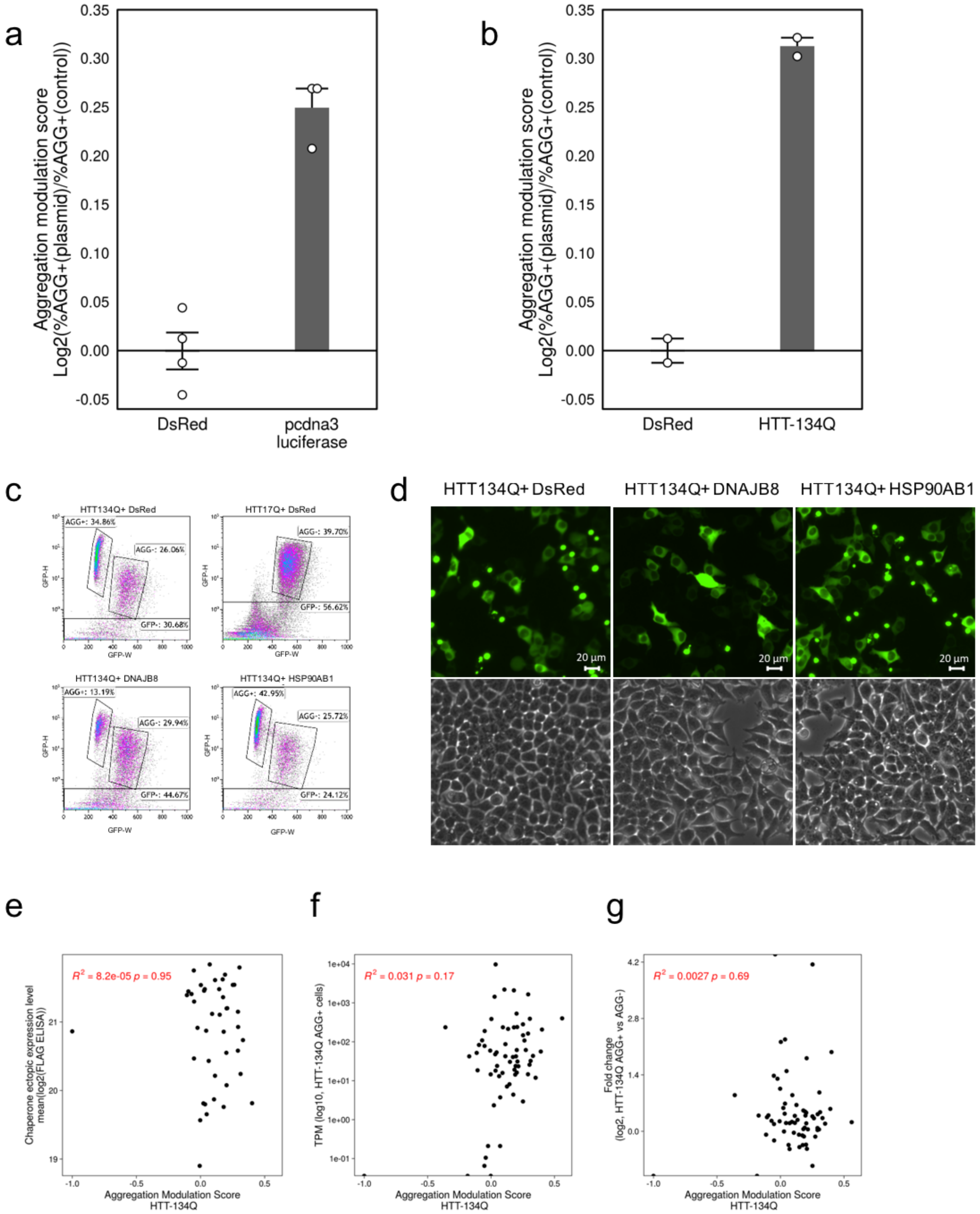

Supplementary Figure 3: HTT-polyQ aggregation modulation screen for chaperone modifiers.

(a-b) DsRed co-expressing cells show lower HTT-134Q-GFP aggregation baseline compared to the co-expression of luciferase (a) or HTT-134Q alone (b), and were thus chosen as a baseline control for the co-expression screen. Data are presented as mean values +/- SEM for n=4/3 for DsRed/luciferase respectively. (a) and n=2 (b) biologically independent samples. Source data are provided as a Source Data file. (c) PulSA analysis of HEK293T cells, 48h after transfection with HTT-17Q-GFP or HTT-134Q-GFP. A distinctive group of cells containing HTT-134Q-GFP aggregated (AGG+) is shown on the left, and is missing in cells expressing the wild-type protein (HTT-17Q-GFP). AGG+ cell population was around 34% in control

DsRed co-expressing cells and reduced to about 13% when DNAJB8 was co-expressed. In contrast, co-expression of HSP90AB1 increased AGG+ fraction to about 43%. (d) Fluorescence microscopy images of HEK293T cells 48h after co-expression of HTT-134Q-GFP with either DsRed control, DNAJB8 or HSP90AB1. DNAJB8 aggregation alleviation is qualitatively observed compared to DsRed control or HSP90AB1, which aggravated the aggregation phenotype. Shown are representative fields from n=3 biologically independent experiments. (e) HTT-134Q Aggregation modulation scores (x-axis) are not explained by the degree of chaperone overexpression (y-axis), as measured using sandwich ELISA assay (log2), in HEK293T cells transfected with HTT-134Q-GFP for 48h. Correlation p-value is indicated. (f) HTT-134Q Aggregation modulation scores (x-axis) do not correlate with basal chaperone expression levels (y-axis), as measured by RNA-seq from HEK293T cells expressing HTT-134Q-GFP for 48h, which have undergone sorting for aggregate containing cells (AGG+). Expression levels represented by log10 TPM values. Correlation p-value is indicated. (g) HTT-134Q Aggregation modulation scores (x-axis) do not correlate with fold changes of chaperones following HTT-134Q aggregation. Fold changes (presented as log2, y-axis) were calculated based on RNA-seq data TPM values as in (f), which were divided by the RNA-seq TPM values of the chaperones in the HTT-134Q-GFP non-aggregating cells (denoted AGG-). Correlation p-value is indicated.

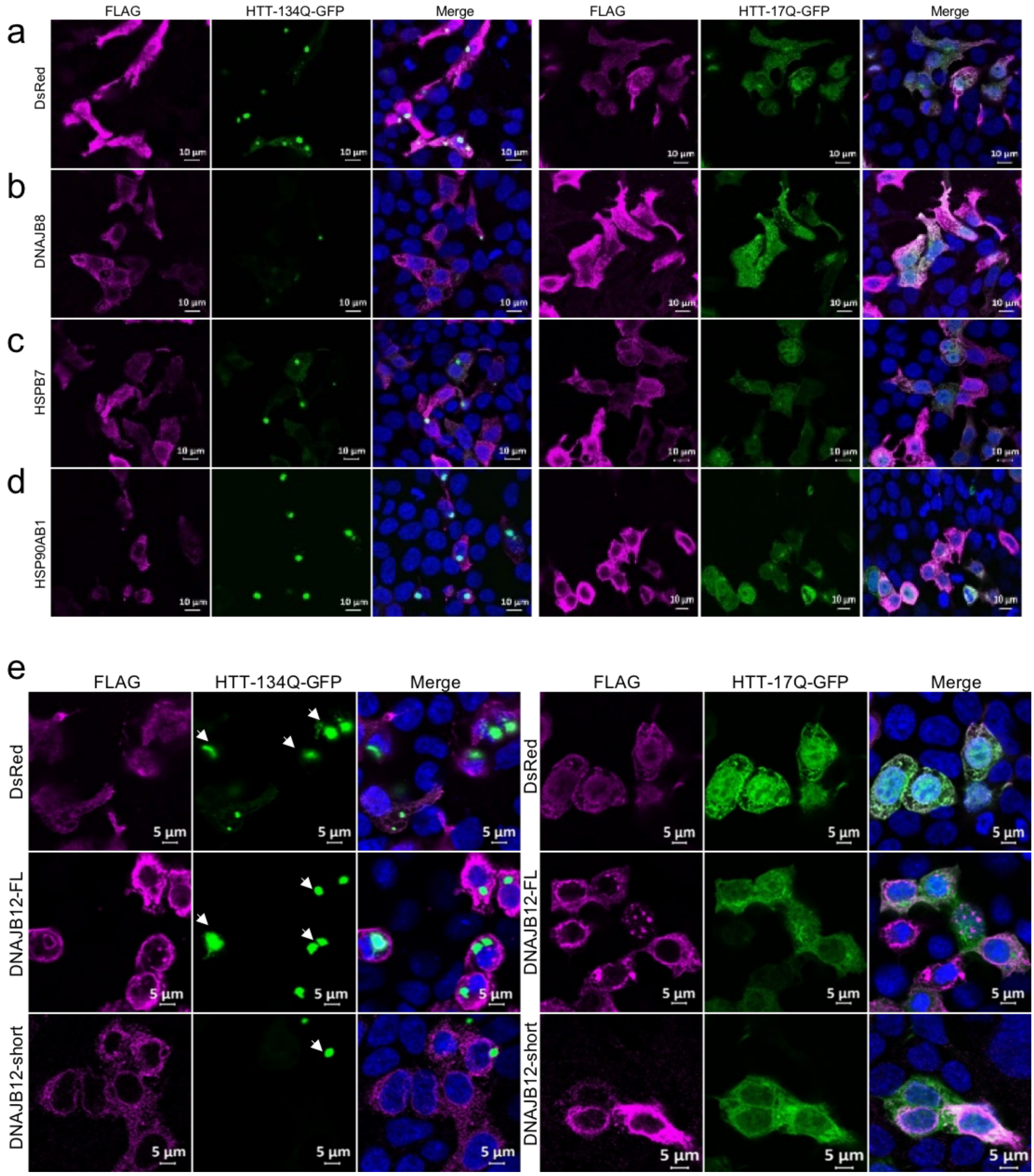

Supplementary Figure 4: HTT-134Q aggregation is modulated by different chaperones

(a-d) Confocal microscopy images of HEK293T cells following 48h transfection with HTT-134Q-GFP or HTT-17Q-GFP (green) co-expressed with FLAG tagged constructs (FLAG in pink), of (a) DsRed control (b) DNAJB8 (c) HSPB7 (d) HSP90AB1. DAPI (blue) marks nuclei. Shown are representative fields from n=2 biologically independent experiments. (e) Immunofluorescence imaging of HTT-134Q-GFP or HTT-17Q-GFP (green) together with FLAG-tagged DsRed, DNAJB12-FL or DNAJB12-short (FLAG in pink). Aggregates are marked with white arrows. Shown are representative fields from n=2 biologically independent experiments.

a

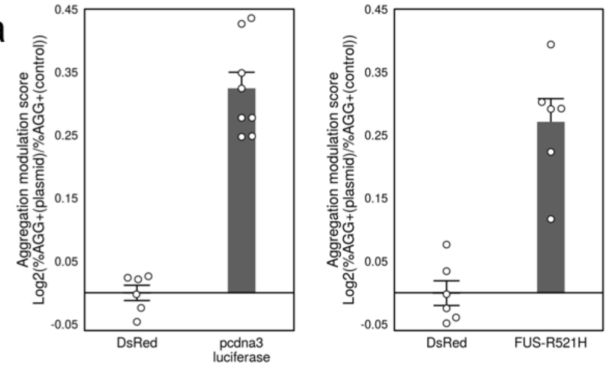

b

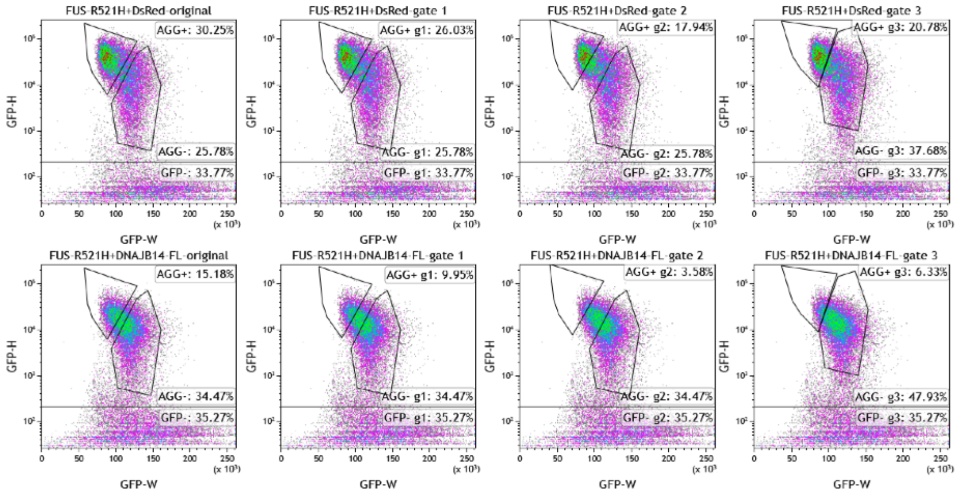

Aggregation modulation score (mean)

	Original gate	Gate 1	Gate 2	Gate 3
**DNAJB14-FL**	-0.819	-1.132	-1.986	-1.675
**BAG3**	-0.668	-0.964	-2.120	-1.513
**HSF1**	-0.530	-0.706	-1.143	-0.968
**DNAJC30**	-0.459	-0.548	-0.761	-0.645

c

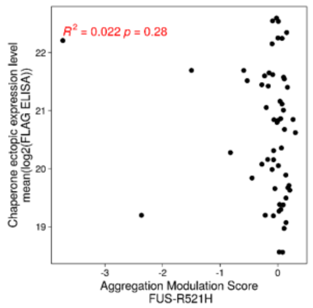

d

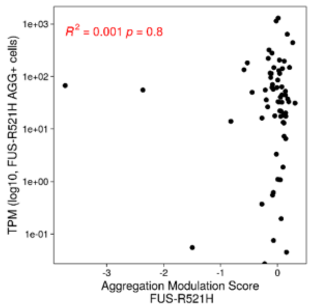

e

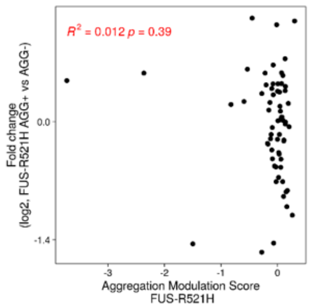

f

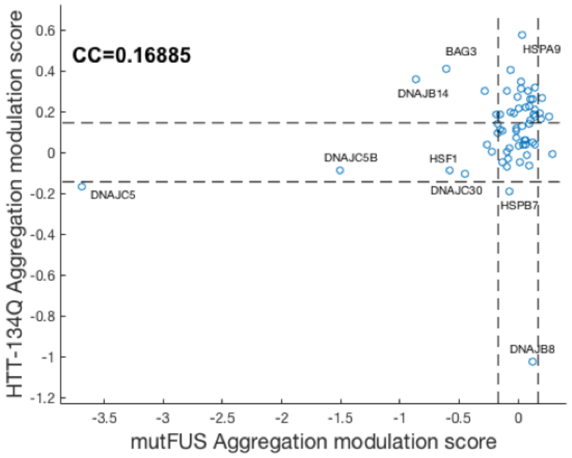
CC=0.16885
BAG3
HSPA9
DNAJB14
DNAJC5B
HSF1
DNAJC30
HSPB7
DNAJC5
DNAJB8
HTT-134Q Aggregation modulation score
mutFUS Aggregation modulation score

g

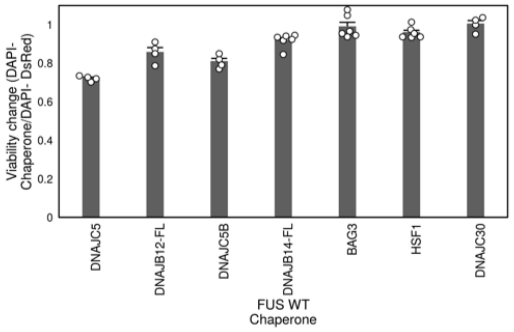
Viability change (DAPI- Chaperone/DAPI- DsRed)
DNAJC5
DNAJB12-FL
DNAJC5B
DNAJB14-FL
BAG3
HSF1
DNAJC30
FUS WT
Chaperone

h

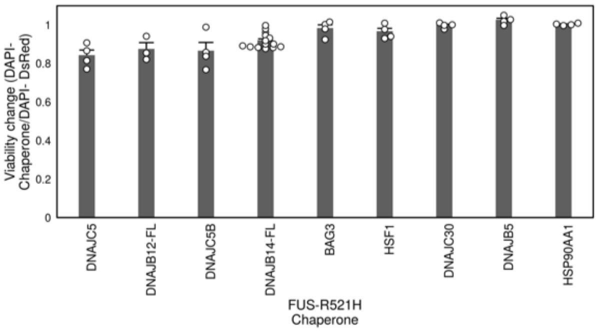
Viability change (DAPI- Chaperone/DAPI- DsRed)
DNAJC5
DNAJB12-FL
DNAJC5B
DNAJB14-FL
BAG3
HSF1
DNAJC30
DNAJB5
HSP90AA1
FUS-R521H
Chaperone

i

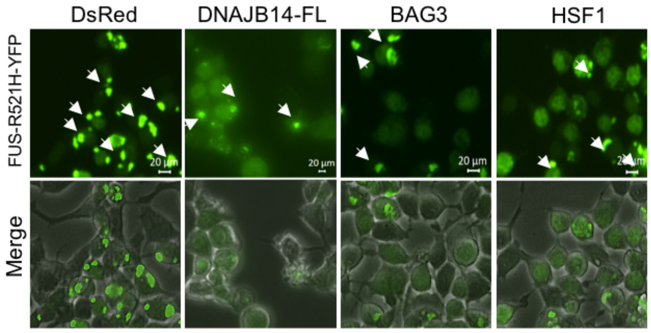
DsRed
DNAJB14-FL
BAG3
HSF1
FUS-R521H-YFP
Merge
20 µm

j

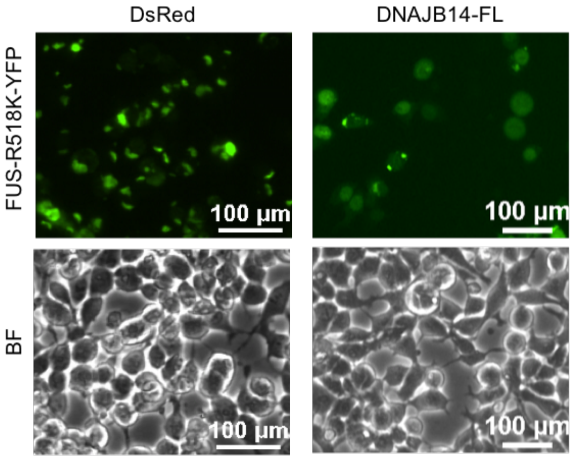
DsRed
DNAJB14-FL
FUS-R518K-YFP
BF
100 µm

k

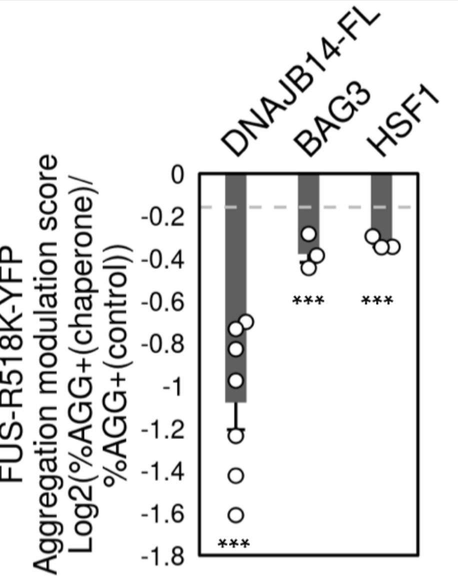
DNAJB14-FL
BAG3
HSF1
FUS-R518K-YFP
Aggregation modulation score
Log2(%AGG+(chaperone)/
%AGG+(control))
***

Supplementary Figure 5: mutFUS aggregation modulation screen reveals chaperone modifiers

(a) DsRed plasmid co-expression together with FUS-R521H-YFP showed lower aggregation baseline compared to luciferase (left) or FUS-R521H alone (right) and was therefore chosen as a baseline control for the co-expression screen. Data are presented as mean values +/- SEM for n=6/8 (DsRed/luciferase, left panel) and n=6 (DsRed/FUS-R521H alone, right panel) biologically independent samples. Source data are provided as a Source Data file. (b) Rescue of FUS-R521H-YFP aggregation by different chaperones is robust to choice of FACS parameter settings. We applied different gates for identification of aggregate positive cell populations – three additional gates are illustrated for DsRed (top row) and DNAJB14-FL expressing cells (middle). We changed the gate settings for all chaperones listed in the table below, which significantly rescued aggregation, as well as for the DsRed controls, and re-calculated the Aggregation modulation score, which is presented in the table (bottom), average of N=4 replicates. All scores remain significant. (c) FUS-R521H-YFP Aggregation modulation scores (x-axis) are not explained by the degree of chaperone overexpression (y-axis, log2), as measured using sandwich ELISA assays, in HEK293T cells transfected with FUS-R521H-YFP for 48h. Correlation p-value is indicated. (d) FUS-R521H-YFP Aggregation modulation scores (x-axis) do not correlate with basal chaperone expression levels (y-axis), as measured by RNA-seq from HEK293T cells expressing FUS-R521H-YFP for 48h, which have undergone sorting for aggregate containing cells (AGG+). Expression levels represented as log10 TPM values. Correlation p-value is indicated. (e) FUS-R521H-YFP Aggregation modulation scores (x-axis) do not correlate with fold changes of chaperones following FUS-R521H-YFP aggregation. Fold changes (presented as log2, y-axis) were calculated based on RNA-seq data TPM values as in (d), which were divided by the RNA-seq TPM values of the chaperones in the FUS-R521H-YFP non-aggregating cells (denoted AGG-). Correlation p-value is indicated. (f) Aggregation modulation score for HTT-134Q plotted against those of FUS-R521H, dashed lines represent the 95% confidence intervals corresponding to each of the aggregating proteins. Correlation between the scores is minimal and rescue chaperones do not overlap. (g-h) Cell viability percentages of top significant chaperones, assayed by DAPI exclusion using FACS. Data are presented as mean values +/- SEM of n=4 (for DNAJC5, DNAJB12-FL DNAJC5B, DNAJC30) and n=6 (for DNAJB14-FL, BAG3, HSF1) biologically independent samples in FUS-WT (g) and n=4 (for DNAJC5, DNAJC5B, BAG3, HSF1, DNAJC30,DNAJB5,HSP90AA1) n=3 (for DNAJB12-FL) and n=14 (DNAJB14-FL) biologically independent samples in FUS-R521H (h) expressing cells. Source data are provided as a Source Data file. (i) Fluorescence microscopy images of HEK293T cells 48h after co-expression of FUS-R521H-YFP with either DsRed, DNAJB14-FL, BAG3 or HSF1. Shown are representative fields from n=3 biologically independent experiments. White arrows mark mutFUS aggregates. (j) Microscopy images of HEK293T cells expressing FUS-R518K-YFP co-transfected with DsRed or DNAJB14-FL respectively. Shown are representative fields from n=2 biologically independent experiments. (k) Aggregation modulation scores for FUS-R518K-YFP co-transfected with DNAJB14-FL, BAG3 or HSF1. Data are presented as mean values +/- SEM n=7/3/3 biologically independent experiments for DNAJB14-FL/BAG3/HSF1 respectively. Dashed lines represent 95% CI as in Fig. 3a. *** - $p<0.003$, empirical p-value (see Methods). Source data are provided as a Source Data file.

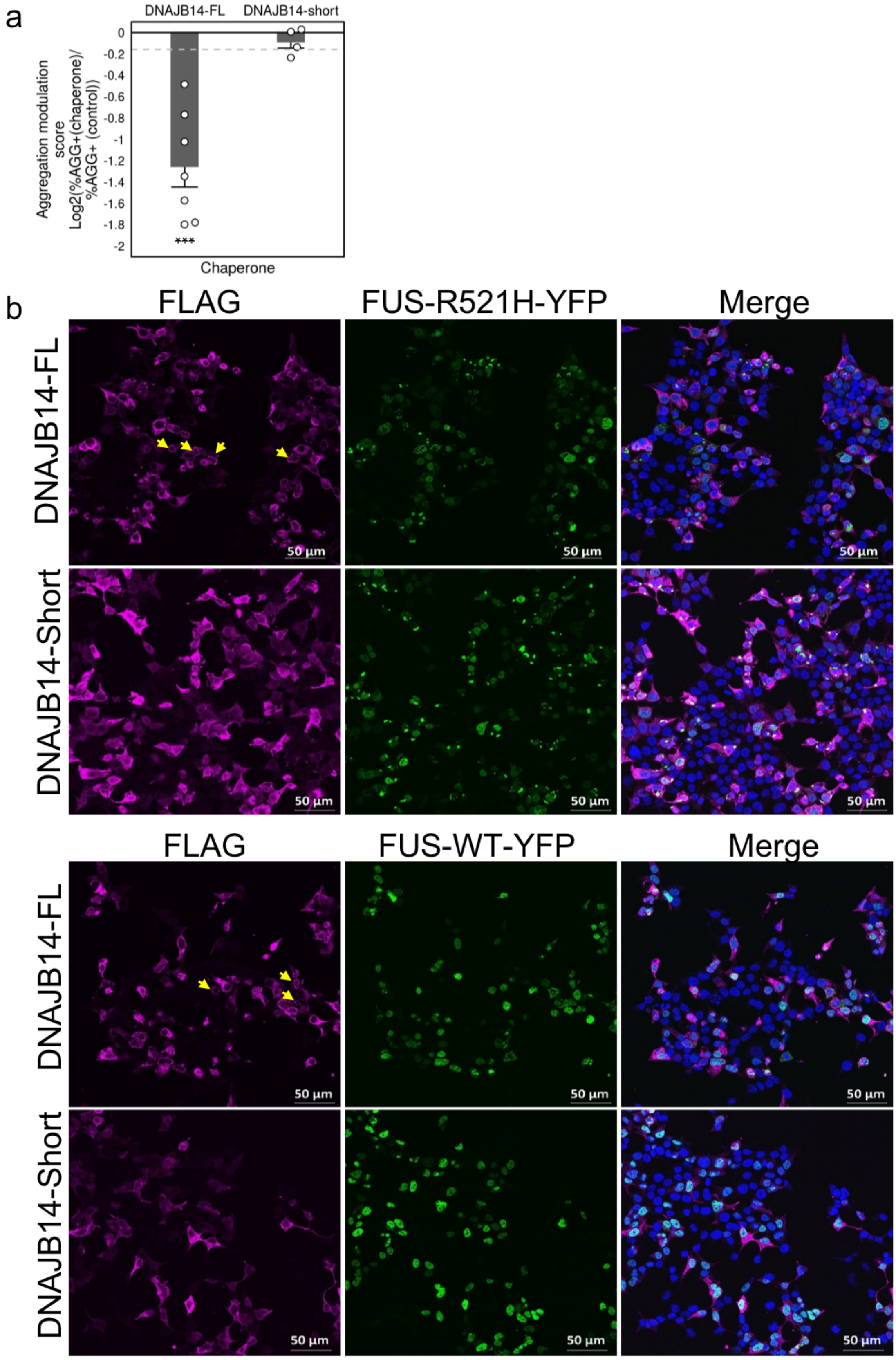
a
DNAJB14-FL
DNAJB14-short
Aggregation modulation score
Log2(%AGG+(chaperone)/%AGG+ (control))
0
-0.2
-0.4
-0.6
-0.8
-1
-1.2
-1.4
-1.6
-1.8
-2
***
Chaperone
b
FLAG
FUS-R521H-YFP
Merge
DNAJB14-FL
DNAJB14-Short
50 µm
FLAG
FUS-WT-YFP
Merge
DNAJB14-FL
DNAJB14-Short
50 µm

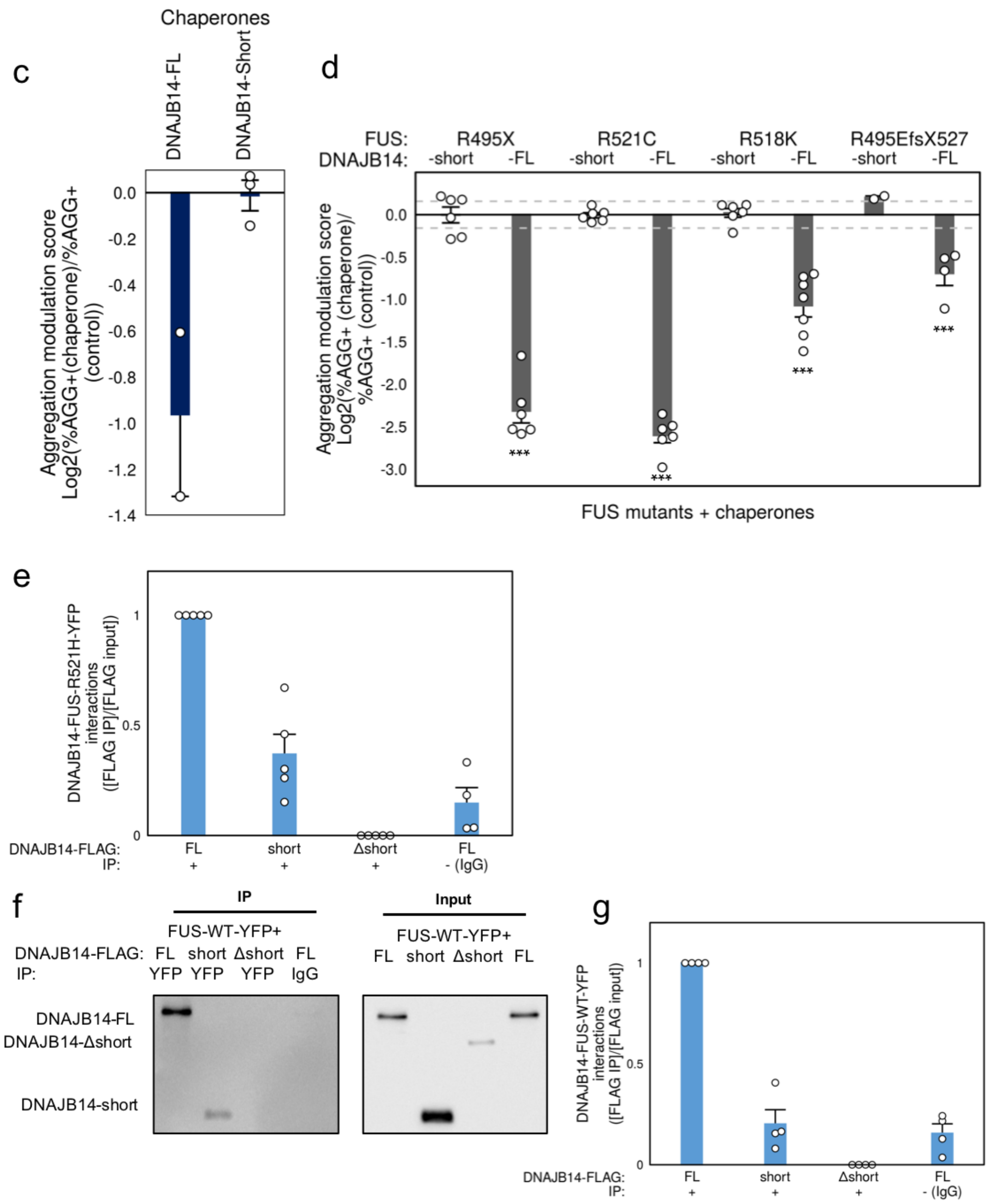
c
Chaperones
DNAJB14-FL
DNAJB14-Short
Aggregation modulation score
Log2(%AGG+(chaperone)/%AGG+ (control))
0.0
-0.2
-0.4
-0.6
-0.8
-1.0
-1.2
-1.4
d
FUS: R495X R521C R518K R495EfsX527
DNAJB14: -short -FL -short -FL -short -FL -short -FL
Aggregation modulation score
Log2(%AGG+ (chaperone)/ %AGG+ (control))
0.0
-0.5
-1.0
-1.5
-2.0
-2.5
-3.0
***
FUS mutants + chaperones
e
DNAJB14-FUS-R521H-YFP interactions ([FLAG IP]/[FLAG input])
1
0.5
0
DNAJB14-FLAG: FL short Δshort FL
IP: + + + - (IgG)
f
IP
Input
FUS-WT-YFP+
DNAJB14-FLAG: FL short Δshort FL
IP: YFP YFP YFP IgG
FUS-WT-YFP+
FL short Δshort FL
DNAJB14-FL
DNAJB14-Δshort
DNAJB14-short
g
DNAJB14-FUS-WT-YFP interactions ([FLAG IP]/[FLAG input])
1
0.5
0
DNAJB14-FLAG: FL short Δshort FL
IP: + + + - (IgG)

h

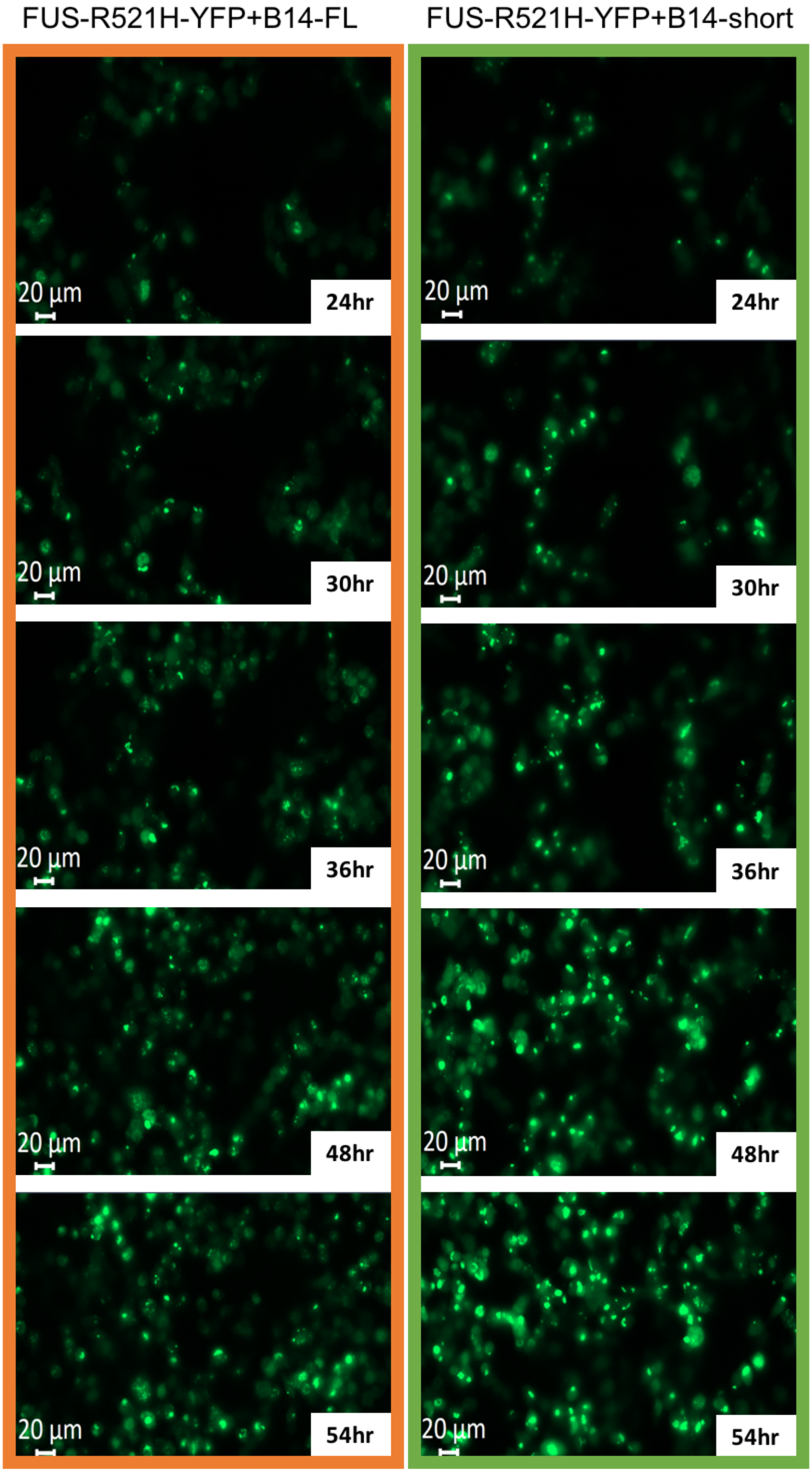

FUS-R521H-YFP+B14-FL
FUS-R521H-YFP+B14-short
20 µm
24hr
30hr
36hr
48hr
54hr

i

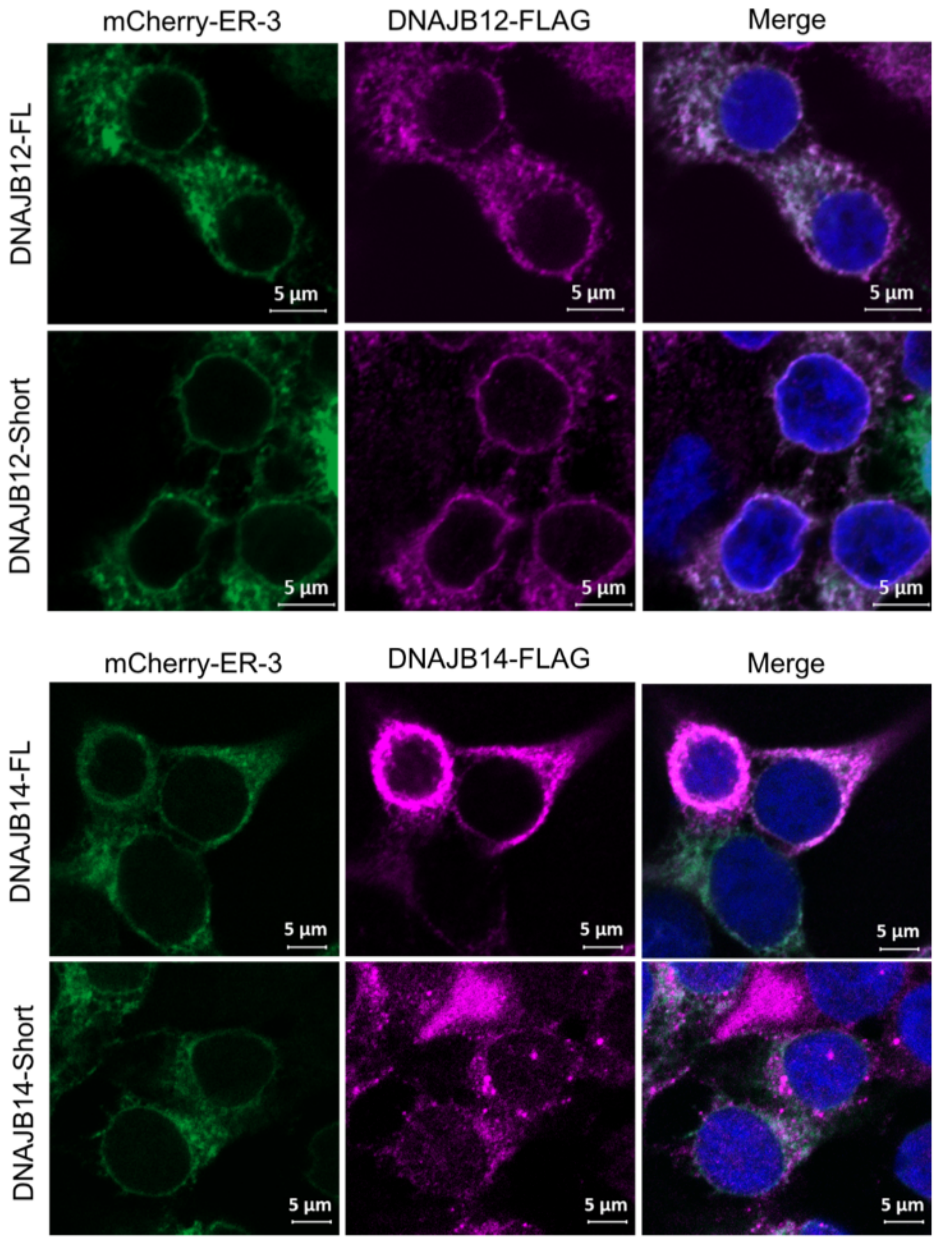
mCherry-ER-3
DNAJB12-FLAG
Merge
DNAJB12-FL
DNAJB12-Short
5 µm
mCherry-ER-3
DNAJB14-FLAG
Merge
DNAJB14-FL
DNAJB14-Short
5 µm

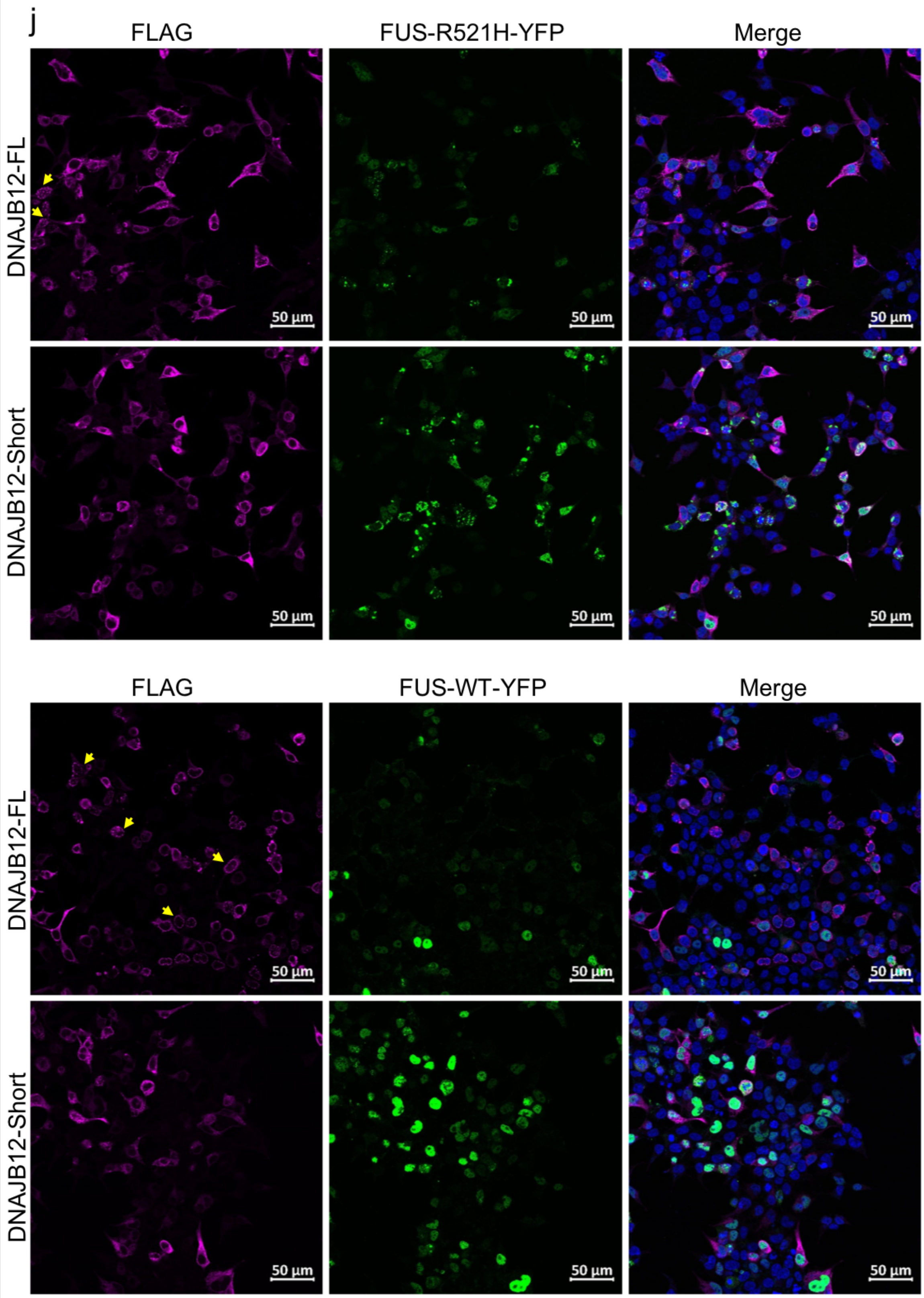
j
FLAG
FUS-R521H-YFP
Merge
DNAJB12-FL
DNAJB12-Short
50 µm
FLAG
FUS-WT-YFP
Merge
DNAJB12-FL
DNAJB12-Short
50 µm

k

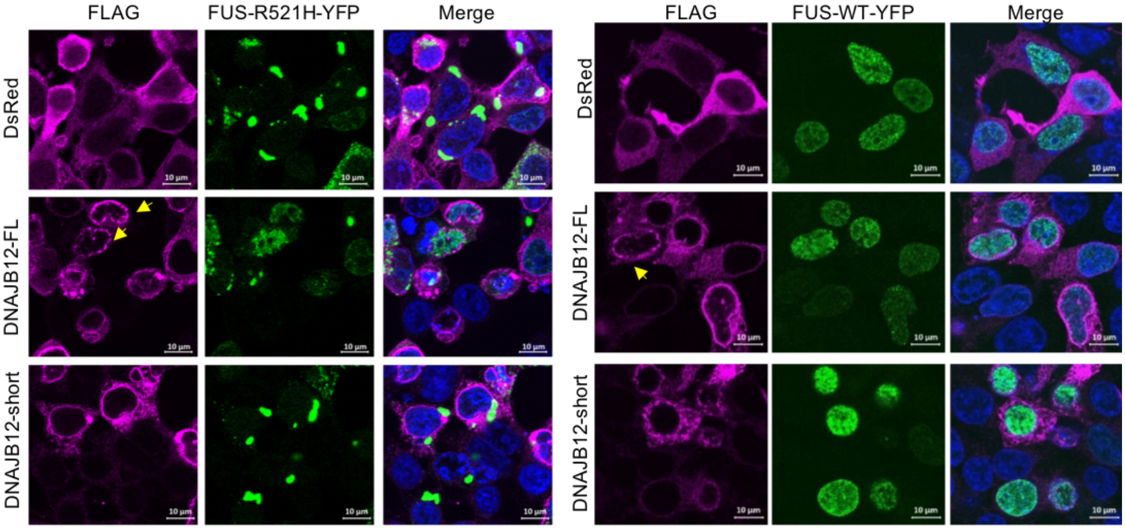
FLAG
FUS-R521H-YFP
Merge
FLAG
FUS-WT-YFP
Merge
DsRed
DNAJB12-FL
DNAJB12-short
DsRed
DNAJB12-FL
DNAJB12-short
10 μm

l

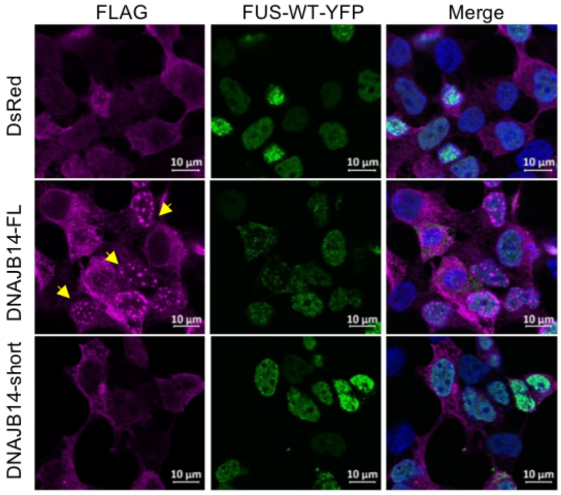
FLAG
FUS-WT-YFP
Merge
DsRed
DNAJB14-FL
DNAJB14-short
10 μm

m

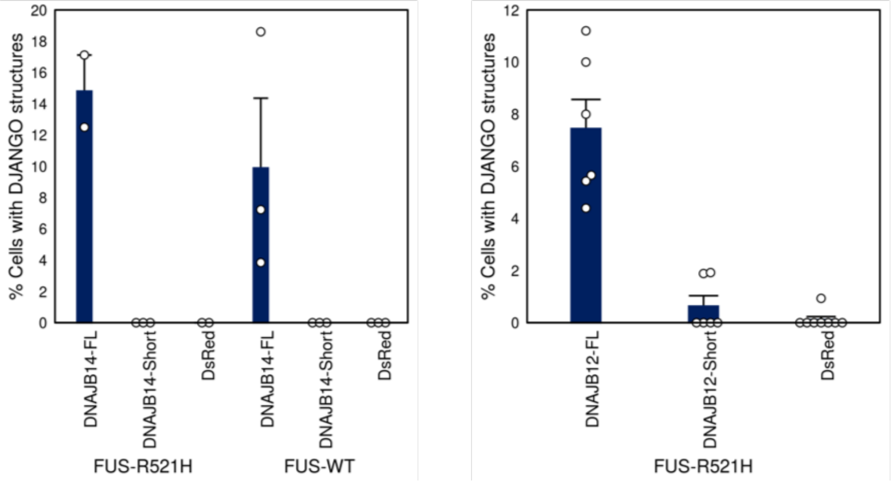
% Cells with DJANGO structures
20
18
16
14
12
10
8
6
4
2
0
DNAJB14-FL
DNAJB14-Short
DsRed
DNAJB14-FL
DNAJB14-Short
DsRed
FUS-R521H
FUS-WT
% Cells with DJANGO structures
12
10
8
6
4
2
0
DNAJB12-FL
DNAJB12-Short
DsRed
FUS-R521H

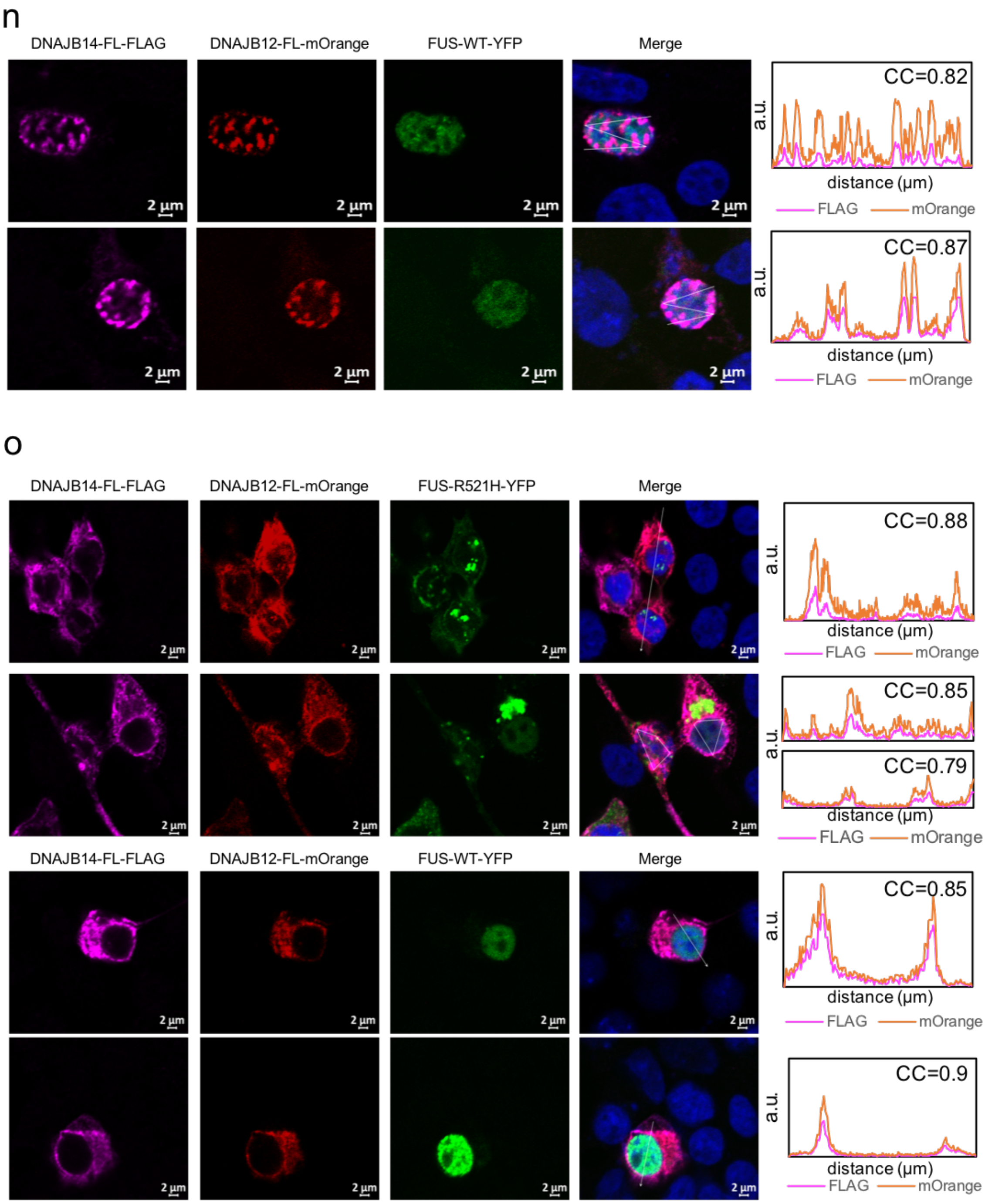
n
DNAJB14-FL-FLAG
DNAJB12-FL-mOrange
FUS-WT-YFP
Merge
2 µm
CC=0.82
CC=0.87
a.u.
distance (µm)
FLAG
mOrange
o
DNAJB14-FL-FLAG
DNAJB12-FL-mOrange
FUS-R521H-YFP
Merge
CC=0.88
CC=0.85
CC=0.79
DNAJB14-FL-FLAG
DNAJB12-FL-mOrange
FUS-WT-YFP
Merge
CC=0.85
CC=0.9

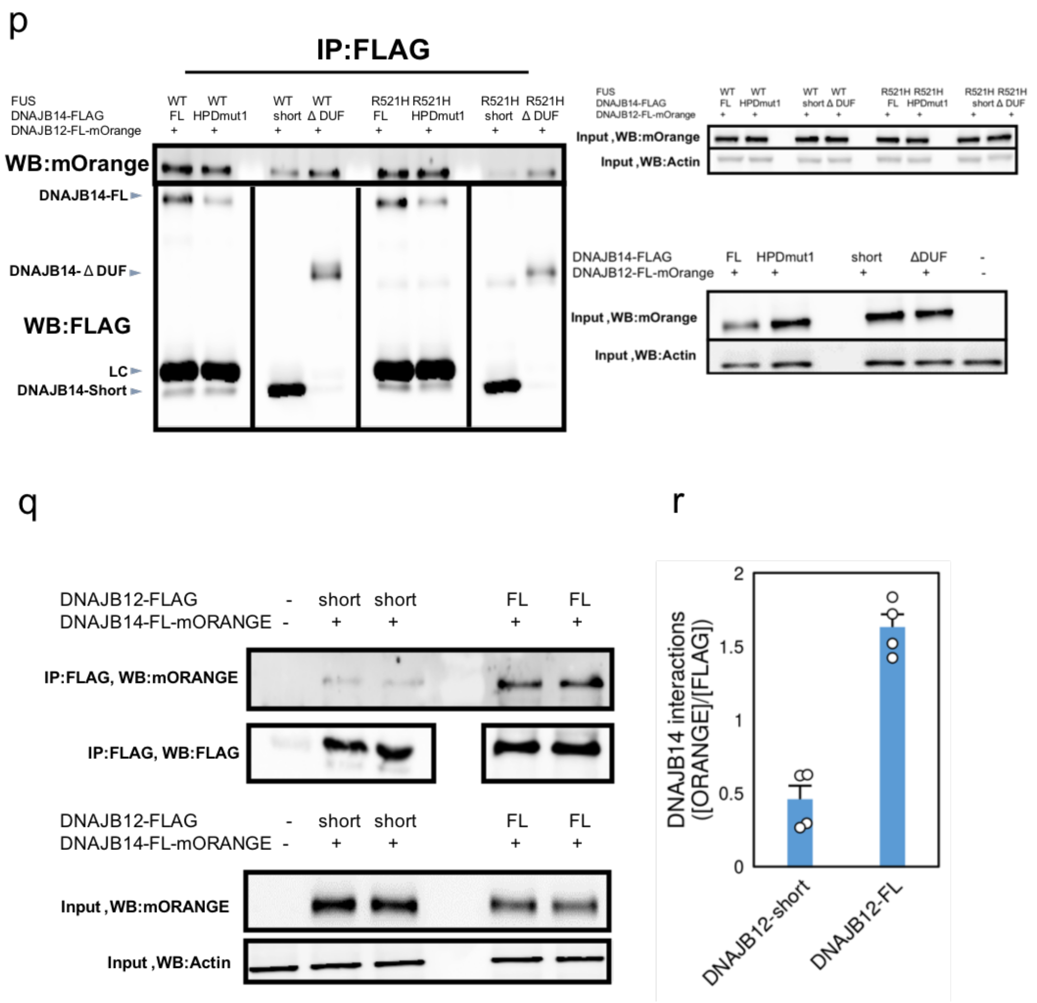

Supplementary Figure 6: DNAJB14 and DNAJB12 full length isoforms interactions and localization

(a) Aggregation modulation scores for FUS-R521H-YFP cells co-transfected with non-tagged DNAJB14-FL or DNAJB14-short. Data are presented as mean values +/- SEM of n=4 and n=7 biologically independent samples (for DNAJB14-short and -FL respectively). Dashed lines represent 95% CI. *** - p<0.003, empirical p-value (see Methods). Source data are provided as a Source Data file. (b) Confocal microscopy of HEK293T cells co-transfected with DNAJB14-FL or DNAJB14-short (pink) together with FUS-R521H or FUS-WT (green). Some DJANGO structures are indicated by yellow arrows. No DJANGOs are observed in the short isoform. Shown are representative fields from n=4 biologically independent experiments. (c) Image analysis quantification recapitulated the aggregation modulation scores of DNAJB14-FL vs. DNAJB14-short (see Methods). Images were taken using confocal microscopy. Data are presented as mean values +/- SEM of n=2 biologically independent samples of DNAJB14-FL (with 72 and 75 cells), n=3 biologically independent samples of DNAJB14-short (with 128,133 and 38 cells), normalized to n=2 biologically independent samples of DsRed controls (with 90 and 126 cells). Source data are provided as a Source Data file. (d) Aggregation modulation scores for the different FUS mutants (as shown) co-transfected with either DNAJB14-FL or DNAJB14-Short. Data are presented as mean values +/- SEM of n=2 (for R495EfsX527+DNAJB14-short), n=4 (for R495EfsX527+DNAJB14-FL), and n=7 (for R518K+DNAJB14-FL) and n=6 (for all the rest) biologically independent samples for . Dashed lines represent 95% CI. *** - p<0.003, empirical p-value (see Methods). Source data are provided as a Source Data file. (e) Signal intensity quantification of DNAJB14 isoforms binding to FUS-R521H-YFP, each normalized to the input FLAG signal. Data are presented as mean values +/- SEM of n=5 experiments for FL and short, n=4 for Δshort and IgG. Densitometry was performed using Fiji. Source data are provided as a Source Data file. (f-g) Co-IP (f) showed that DNAJB14-FL interacted with FUS-WT-YFP while DNAJB14-short showed a minimal level of interaction. (g) As in (e), for FUS-WT-YFP, data are presented

as mean values +/- SEM of n=5 biologically independent experiments. Source data are provided as a Source Data file. (h) Live cell fluorescence microscopy imaging using automated boxed microscope (see Methods). Cells were co-transfected with FUS-R521H-YFP and DNAJB14-FL or DNAJB14-short respectively, then were imaged 24h post transfection until 54hrs post transfection. Times are indicated. In the presence of DNAJB14-FL, aggregates are generated in lower rates, and rarely disappear. Shown are representative fields from n=2 biologically independent movies. (i) ER localization of DNAJB14 (bottom) and DNAJB12 (top), stained with anti-FLAG (pink) – shown via colocalization with the ER marker mCherry-ER-3 (green), containing the signal peptide of CALR (see Methods) in immunofluorescence imaging. Shown are representative cells from n=3 biologically independent samples. (j-k) Same as in (b) for DNAJB12-FL and DNAJB12-short. Shown are representative fields from n=2 biologically independent experiments. (k) IF images as in (j) with larger magnification. (l) IF for DNAJB14-FL and -short together with WT FUS (as in Fig. 4c). (m) Image analysis (see Methods) showed that DNAJB14-short does not form any DJANGO structures. Data are presented as mean values +/- SEM for n=2/2/3 (FUS-R521H+DNAJB14-FL/DsRed/DNAJB14-short respectively), n=3 (for FUS-WT+DNAJB14-FL/DsRed/DNAJB14-short) and n=6/6/8 (FUS-R521H+DNAJB12-FL/DNAJB12-short/DsRed respectively) biologically independent samples. A similar analysis as also performed for DNAJB12-FL and -short (right panel). Source data are provided as a Source Data file. (n) DNAJB12-FL tagged with mOrange (red) and DNAJB14-FL tagged with FLAG (pink) showing DJANGO structures in cells transfected with the WT FUS. Cross correlation plots are shown together with the correlation coefficient between DNAJB14 and DNAJB12. (o) The FL isoforms of both DNAJB12 and DNAJB14 are shown to also co-localize in cells where they demonstrate an ER localization pattern, both in the presence of FUS-R521H (green, top) and FUS-WT (green, bottom) cells. Cross correlation plots are shown together with the correlation coefficient (CC) between DNAJB14 and DNAJB12. (p) As in Fig. 4f, in the presence of WT and mutFUS. This represents 2 biologically independent replicates to Fig. 4f. Also shown are input WBs for Fig. 4f and this panel. White band (lane 3,6,9) shows a molecular weight marker of 70KDa. Source data are provided as a Source Data file. (q-r) Co-IP (q) showed that DNAJB12-FL interacts with DNAJB14-FL (tagged with mOrange), while DNAJB12-short had negligible interaction with DNAJB14-FL. We note that the sizes of the two isoforms are very different, as seen in Fig. 2d, and S6p. White band (lane 4) shows a molecular weight marker of 70KDa. Representative WB is shown out of n=3 biologically independent experiments. Source data are provided as a Source Data file. (r) Quantification of interaction was performed using Fiji, where densitometry of mOrange band was normalized to that of the FLAG band (see Methods). Data are presented as mean values +/- SEM of n=4 biologically independent samples. Source data are provided as a Source Data file.

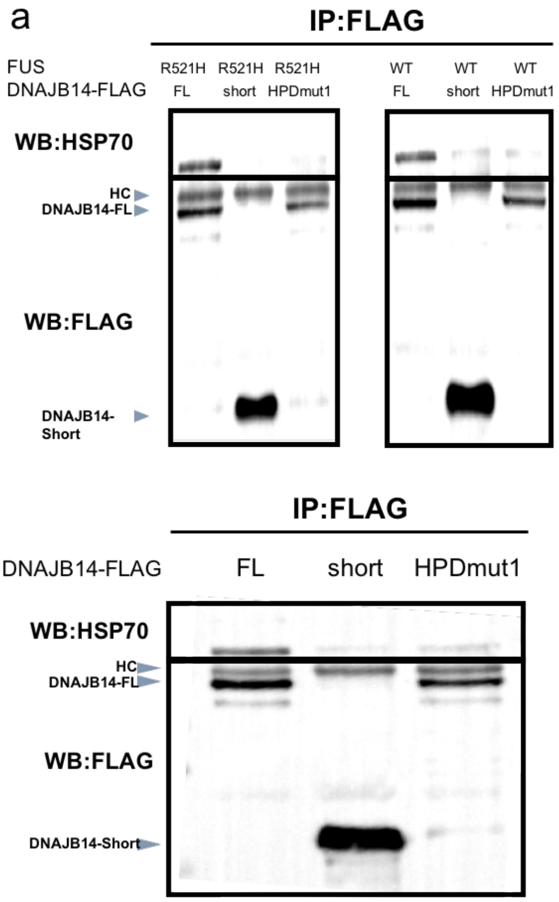
a
IP:FLAG
FUS
DNAJB14-FLAG
R521H FL
R521H short
R521H HPDmut1
WT FL
WT short
WT HPDmut1
WB:HSP70
HC
DNAJB14-FL
WB:FLAG
DNAJB14-Short
IP:FLAG
DNAJB14-FLAG
FL
short
HPDmut1
WB:HSP70
HC
DNAJB14-FL
WB:FLAG
DNAJB14-Short

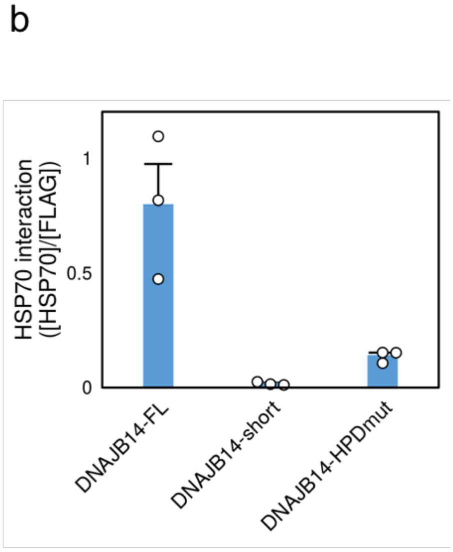
b
HSP70 interaction ([HSP70]/[FLAG])
1
0.5
0
DNAJB14-FL
DNAJB14-short
DNAJB14-HPDmut

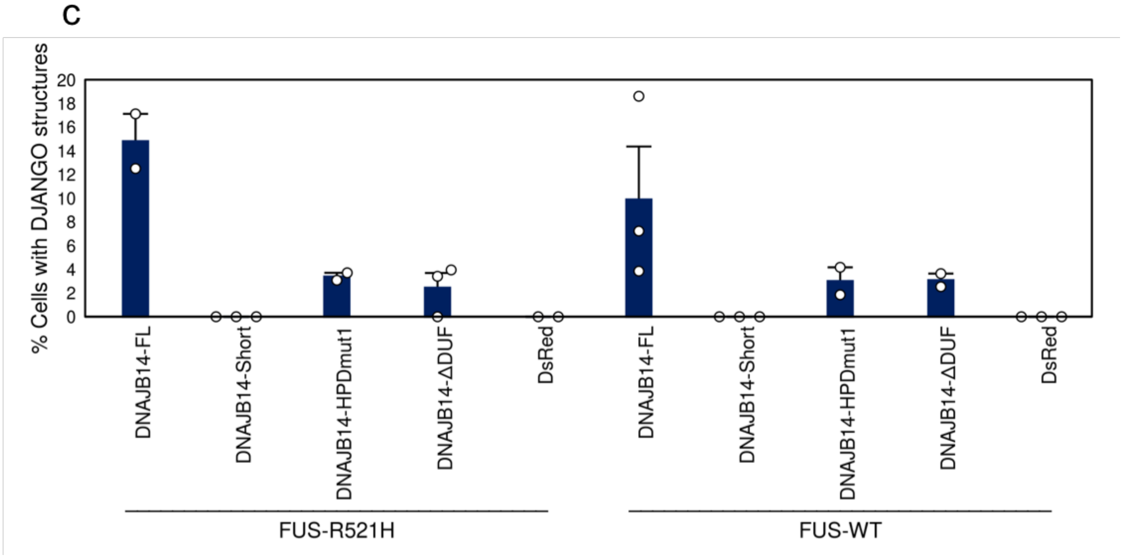
c
% Cells with DJANGO structures
20
18
16
14
12
10
8
6
4
2
0
DNAJB14-FL
DNAJB14-Short
DNAJB14-HPDmut1
DNAJB14-ΔDUF
DsRed
DNAJB14-FL
DNAJB14-Short
DNAJB14-HPDmut1
DNAJB14-ΔDUF
DsRed
FUS-R521H
FUS-WT

d

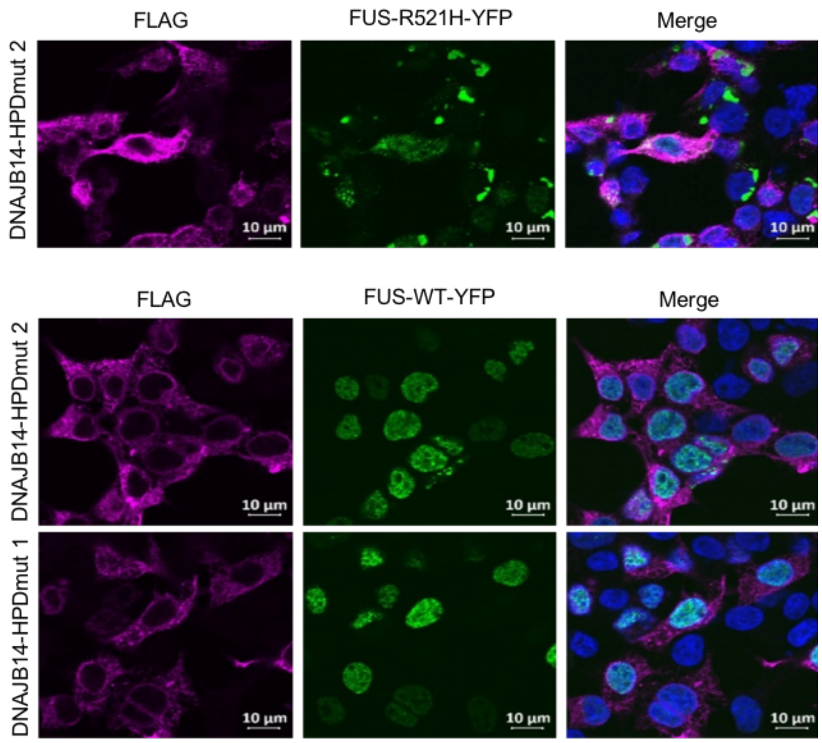

e

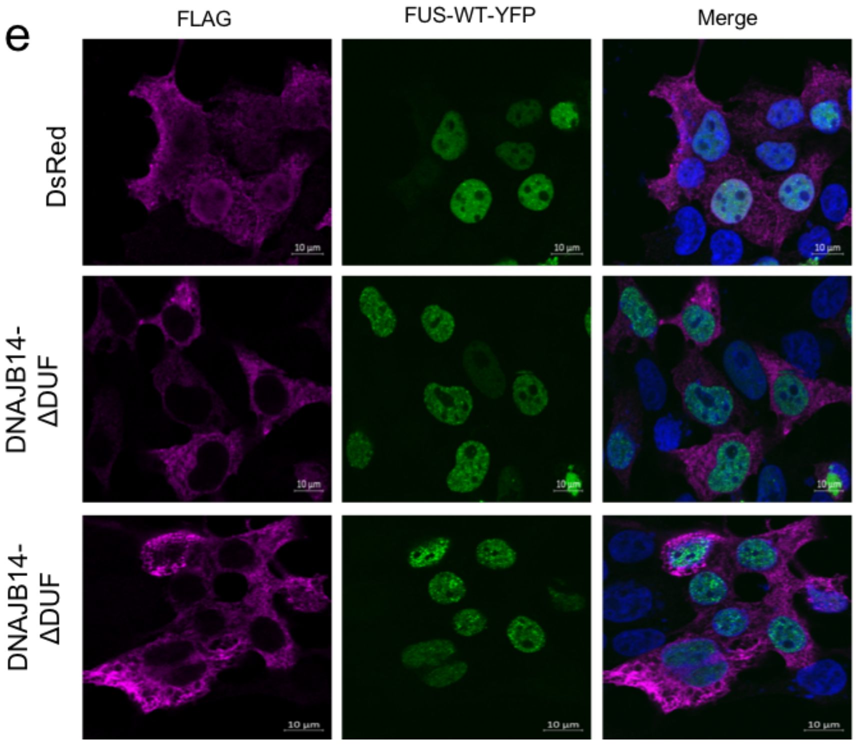

Supplementary Figure 7: DNAJB14 isoforms interact differently with the endogenous HSP70.

(a) Co-IP of cells co-transfected with either WT or mutant FUS with three different isoforms of FLAG tagged DNAJB14 (FL, short, HPDmut1). Interaction between exogenous DNAJB14 and the endogenous

HSP70 is shown in the upper panel. DNAJB14 signal was assayed using anti-FLAG antibody WB in the lower panel. HC-Heavy chain. Source data are provided as a Source Data file. (b) Signal intensity quantification of HSP70 bound to the DNAJB14 different isoforms, all normalized to FLAG signal. Data are presented as mean values +/- SEM of n=3 biologically independent samples shown in panel a. Densitometry was performed using Fiji. Source data are provided as a Source Data file. (c) Percentage of DJANGOs containing cells was calculated using image analysis of IF images, as in Fig. S6m, showing the same data but with additional mutants. Data are presented as mean values +/- SEM for n=2/3/2/3/2 (FUS-R521H+DNAJB14-FL/-short/-HPDmut1/-ΔDUF/DsRed respectively), n=3/3/2/2/3 (FUS-WT+DNAJB14-FL/-short/-HPDmut1/-ΔDUF/DsRed respectively) biologically independent samples. Source data are provided as a Source Data file. (d-e) As in Fig. 4c, for DNAJB14-HPDmut2 with FUS-R521H-YFP, and both HPD mutants together with WT-FUS (d), as well as DNAJB14 ΔDUF with WT-FUS(e). Shown are representative fields from n=4/2 biologically independent experiments (for d and e respectively).

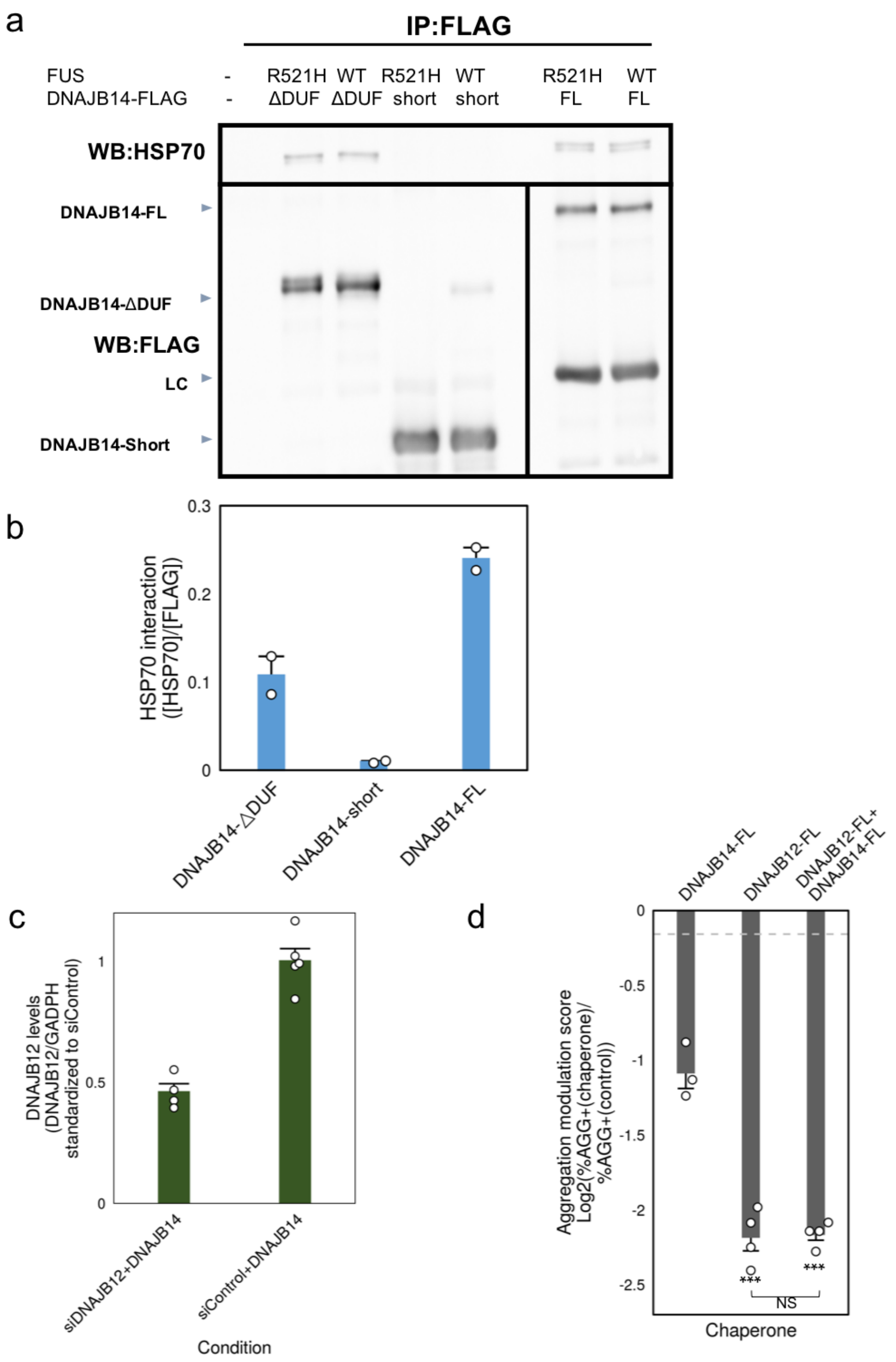

Supplementary Figure 8: DNAJB14 - DNAJB12 complex inter-dependence in protection from mutant FUS aggregation

(a) Co-IP of cells co-transfected with either FUS-WT or FUS-R521H together with FLAG tagged DNAJB14 isoforms (FL, short, or ΔDUF). DNAJB14 isoforms were pulled down using anti-FLAG antibody. Interaction between HSP70 and DNAJB14 isoforms was assayed using WB for endogenous HSP70 (upper panel). DNAJB14 signal was assayed by anti-FLAG antibody WB for the different isoforms (lower panel, LC- Light chain) see arrows: Membrane containing DNAJB14-FL– used with secondary antibody LC fragment specific. Membrane containing DNAJB14-short and ΔDUF – used with secondary

antibody FC fragment specific. Source data are provided as a Source Data file. (b) Quantification HSP70 interactions, HSP70 bands divided by FLAG band intensities, was performed using Fiji. Data are presented as mean values +/- SEM of n=2 biologically independent samples from panel a. Source data are provided as a Source Data file. (c) Fold changes in mRNA levels of the DNAJB12-FL gene normalized to the GAPDH were assayed using qPCR for DNAJB12 knockdown samples (siDNAJB12) and control samples (siControl). Data are presented as mean values +/- SEM of n=3 biologically independent experiments, each standardized to the siControl sample. Source data are provided as a Source Data file. (d) Aggregation modulation scores for HEK293T cells transfected with FUS-R521H-YFP and DNAJB12-FL, DNAJB14-FL or both. The mutual overexpression of both DNAJB12-FL and DNAJB14-FL shows a similar extent of aggregation protection as that of DNAJB12-FL alone. Data represent mean +/- SEM for n=4/4/3 (for DNAJB12+DNAJB14/DNAJB12/DNAJB14 respectively) biologically independent samples. Dashed lines represent 95% CIs as in Fig. 3a. *** - p<0.003, empirical p-value (see Methods).

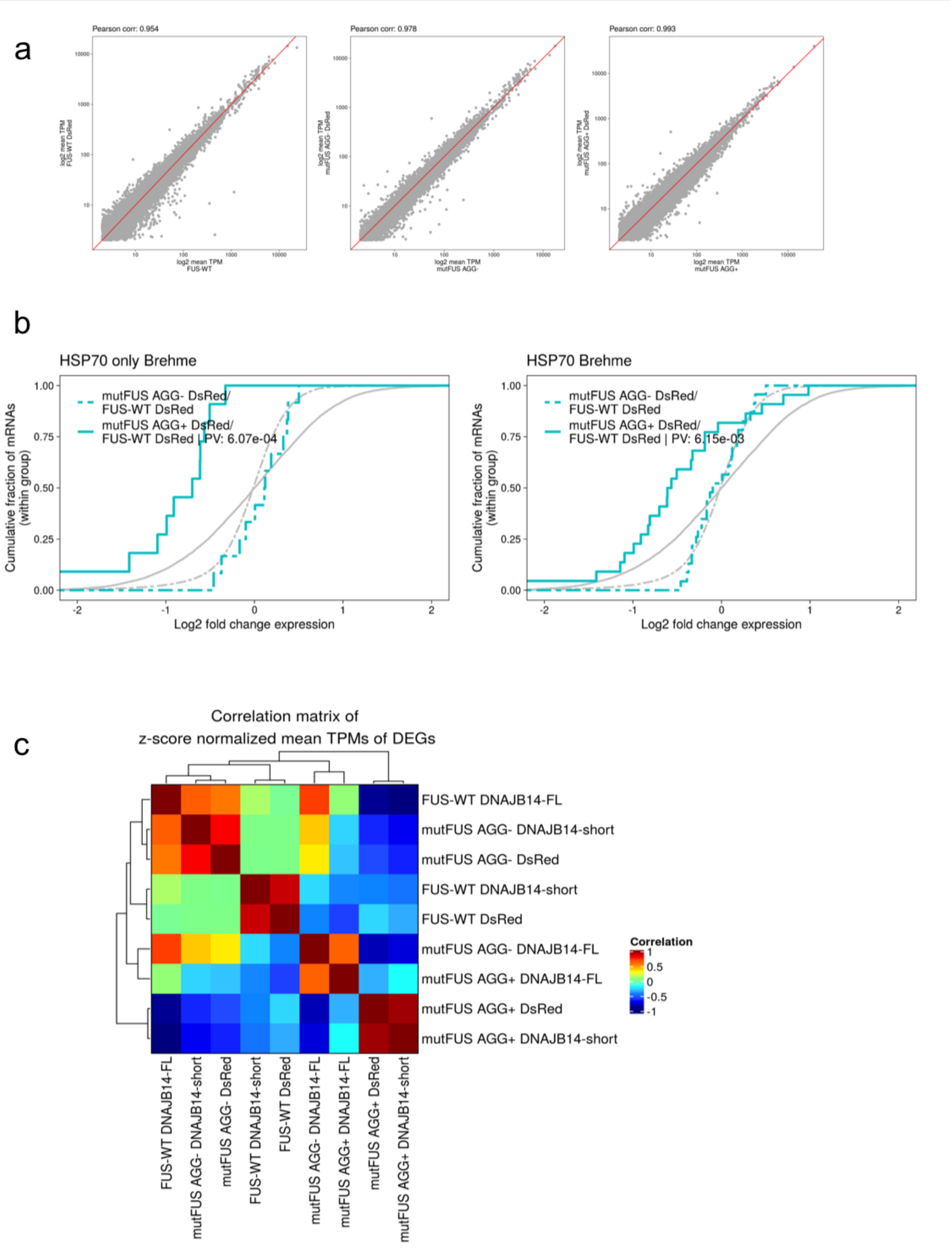
a
Pearson corr: 0.954
log2 mean TPM FUS-WT DsRed
log2 mean TPM FUS-WT
Pearson corr: 0.978
log2 mean TPM mutFUS AGG- DsRed
log2 mean TPM mutFUS AGG-
Pearson corr: 0.993
log2 mean TPM mutFUS AGG+ DsRed
log2 mean TPM mutFUS AGG+
b
HSP70 only Brehme
mutFUS AGG- DsRed/ FUS-WT DsRed
mutFUS AGG+ DsRed/ FUS-WT DsRed | PV: 6.07e-04
Cumulative fraction of mRNAs (within group)
Log2 fold change expression
HSP70 Brehme
mutFUS AGG- DsRed/ FUS-WT DsRed
mutFUS AGG+ DsRed/ FUS-WT DsRed | PV: 6.15e-03
Cumulative fraction of mRNAs (within group)
Log2 fold change expression
c
Correlation matrix of
z-score normalized mean TPMs of DEGs
FUS-WT DNAJB14-FL
mutFUS AGG- DNAJB14-short
mutFUS AGG- DsRed
FUS-WT DNAJB14-short
FUS-WT DsRed
mutFUS AGG- DNAJB14-FL
mutFUS AGG+ DNAJB14-FL
mutFUS AGG+ DsRed
mutFUS AGG+ DNAJB14-short
Correlation
1
0.5
0
-0.5
-1

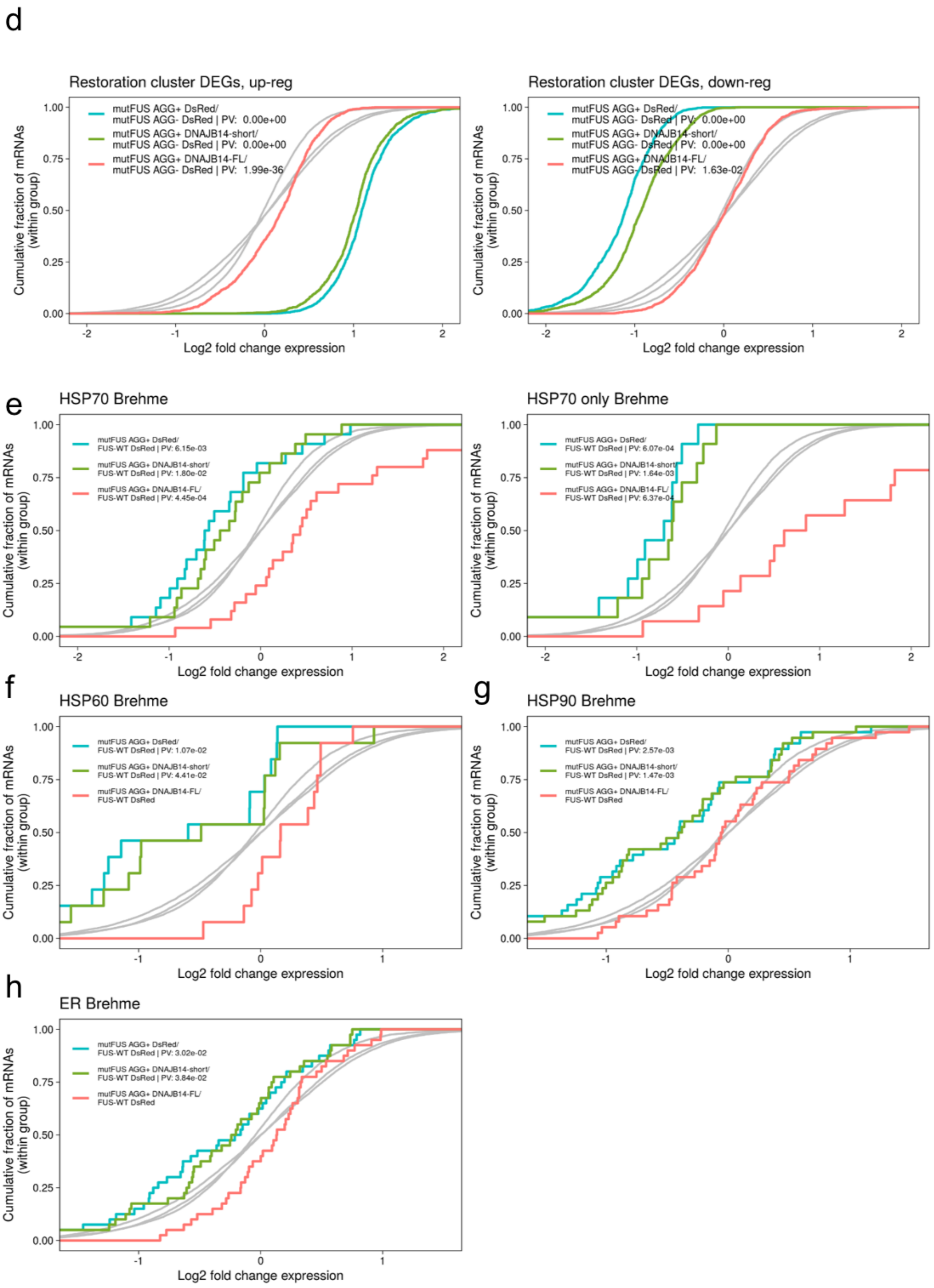
d
Restoration cluster DEGs, up-reg
mutFUS AGG+ DsRed/ mutFUS AGG- DsRed | PV: 0.00e+00
mutFUS AGG+ DNAJB14-short/ mutFUS AGG- DsRed | PV: 0.00e+00
mutFUS AGG+ DNAJB14-FL/ mutFUS AGG- DsRed | PV: 1.99e-36
Cumulative fraction of mRNAs (within group)
Log2 fold change expression
Restoration cluster DEGs, down-reg
mutFUS AGG+ DsRed/ mutFUS AGG- DsRed | PV: 0.00e+00
mutFUS AGG+ DNAJB14-short/ mutFUS AGG- DsRed | PV: 0.00e+00
mutFUS AGG+ DNAJB14-FL/ mutFUS AGG- DsRed | PV: 1.63e-02
Cumulative fraction of mRNAs (within group)
Log2 fold change expression
e
HSP70 Brehme
mutFUS AGG+ DsRed/ FUS-WT DsRed | PV: 6.15e-03
mutFUS AGG+ DNAJB14-short/ FUS-WT DsRed | PV: 1.80e-02
mutFUS AGG+ DNAJB14-FL/ FUS-WT DsRed | PV: 4.45e-04
Cumulative fraction of mRNAs (within group)
Log2 fold change expression
HSP70 only Brehme
mutFUS AGG+ DsRed/ FUS-WT DsRed | PV: 6.07e-04
mutFUS AGG+ DNAJB14-short/ FUS-WT DsRed | PV: 1.64e-03
mutFUS AGG+ DNAJB14-FL/ FUS-WT DsRed | PV: 6.37e-04
Cumulative fraction of mRNAs (within group)
Log2 fold change expression
f
HSP60 Brehme
mutFUS AGG+ DsRed/ FUS-WT DsRed | PV: 1.07e-02
mutFUS AGG+ DNAJB14-short/ FUS-WT DsRed | PV: 4.41e-02
mutFUS AGG+ DNAJB14-FL/ FUS-WT DsRed
Cumulative fraction of mRNAs (within group)
Log2 fold change expression
g
HSP90 Brehme
mutFUS AGG+ DsRed/ FUS-WT DsRed | PV: 2.57e-03
mutFUS AGG+ DNAJB14-short/ FUS-WT DsRed | PV: 1.47e-03
mutFUS AGG+ DNAJB14-FL/ FUS-WT DsRed
Cumulative fraction of mRNAs (within group)
Log2 fold change expression
h
ER Brehme
mutFUS AGG+ DsRed/ FUS-WT DsRed | PV: 3.02e-02
mutFUS AGG+ DNAJB14-short/ FUS-WT DsRed | PV: 3.84e-02
mutFUS AGG+ DNAJB14-FL/ FUS-WT DsRed
Cumulative fraction of mRNAs (within group)
Log2 fold change expression

i

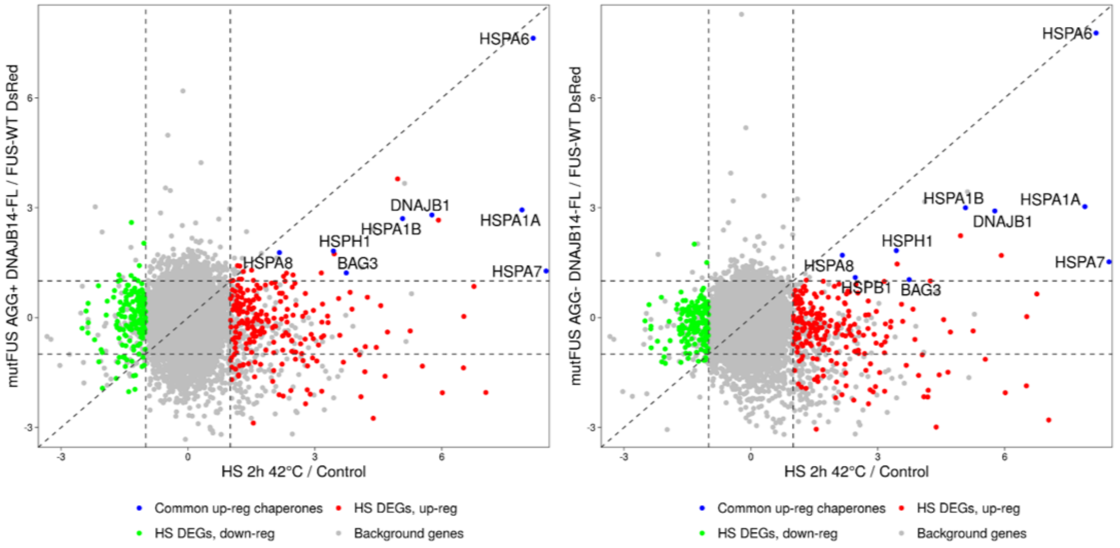
mutFUS AGG+ DNAJB14-FL / FUS-WT DsRed
HS 2h 42°C / Control
HSPA6
DNAJB1
HSPA1A
HSPA1B
HSPH1
HSPA8
BAG3
HSPA7
mutFUS AGG- DNAJB14-FL / FUS-WT DsRed
HSPB1
Common up-reg chaperones
HS DEGs, up-reg
HS DEGs, down-reg
Background genes

j

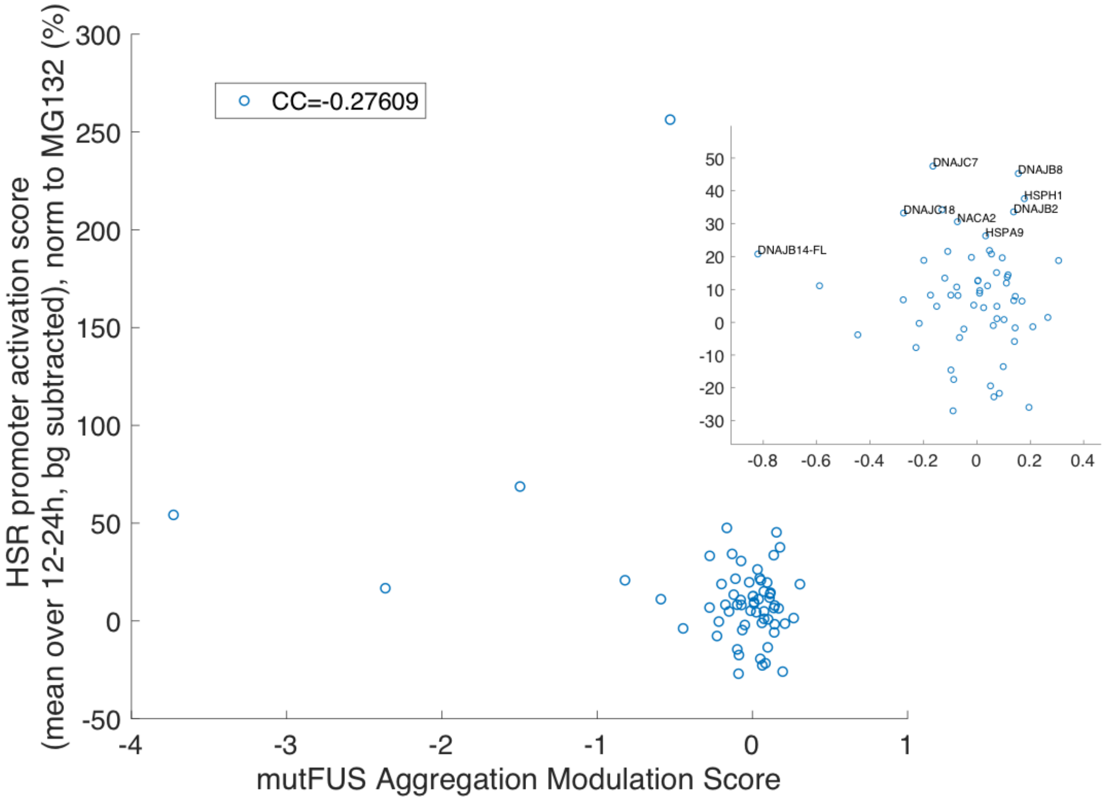
HSR promoter activation score
(mean over 12-24h, bg subtracted), norm to MG132 (%)
mutFUS Aggregation Modulation Score
CC=-0.27609
DNAJC7
DNAJB8
HSPH1
DNAJC18
DNAJB2
NACA2
HSPA9
DNAJB14-FL

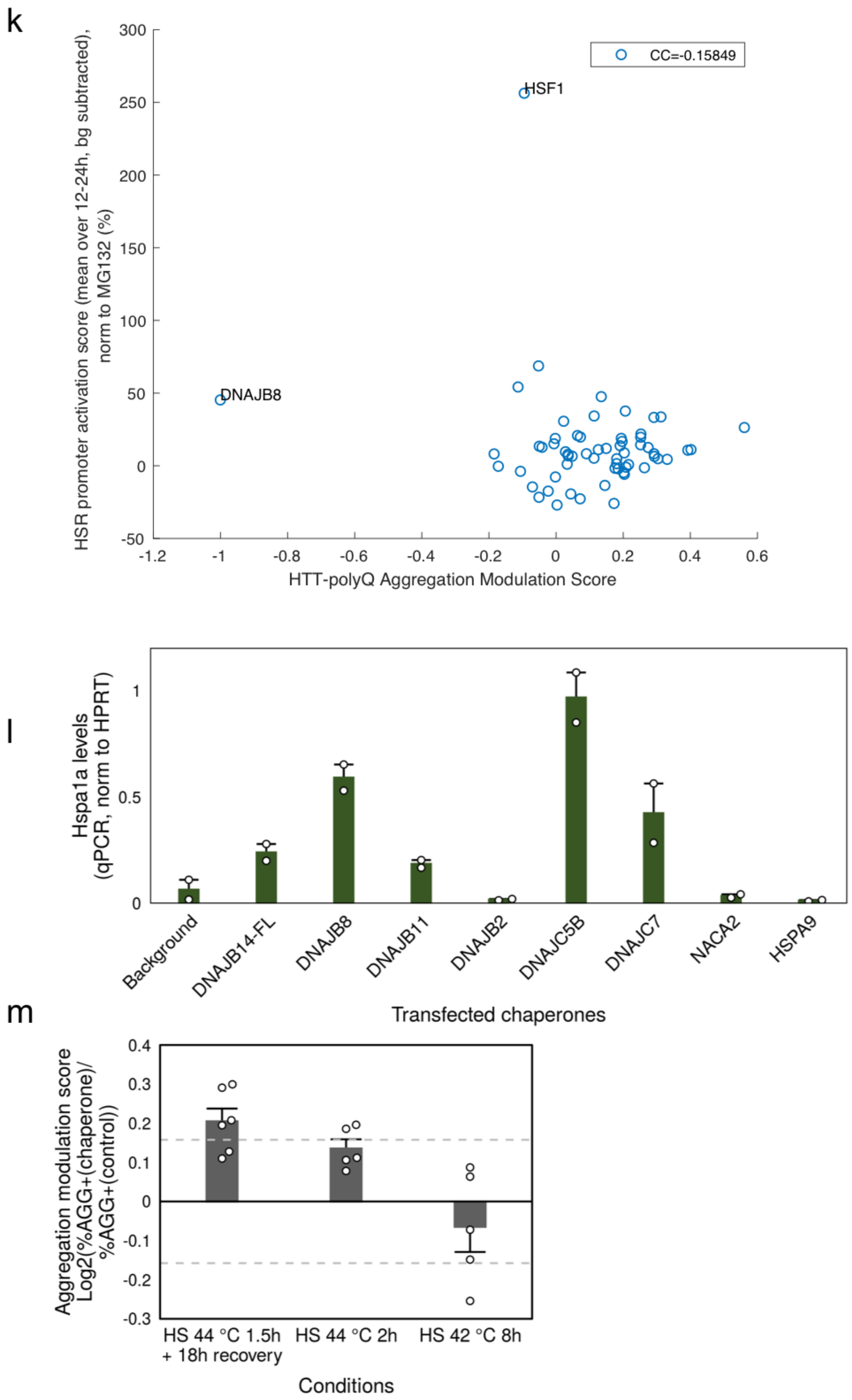
k
HSR promoter activation score (mean over 12-24h, bg subtracted), norm to MG132 (%)
CC=-0.15849
HSF1
DNAJB8
300
250
200
150
100
50
0
-50
-1.2
-1
-0.8
-0.6
-0.4
-0.2
0
0.2
0.4
0.6
HTT-polyQ Aggregation Modulation Score
l
Hspa1a levels (qPCR, norm to HPRT)
1
0.5
0
Background
DNAJB14-FL
DNAJB8
DNAJB11
DNAJB2
DNAJC5B
DNAJC7
NACA2
HSPA9
Transfected chaperones
m
Aggregation modulation score Log2(%AGG+(chaperone)/ %AGG+(control))
0.4
0.3
0.2
0.1
0
-0.1
-0.2
-0.3
HS 44 °C 1.5h + 18h recovery
HS 44 °C 2h
HS 42 °C 8h
Conditions

Supplementary Figure 9: DNAJB14-FL restores deteriorated proteostasis caused by mutFUS aggregation

(a) Scatter plots show high correlation between the two RNA-seq experiments, of mRNA expression in FUS-WT or mutFUS expressing cells (either aggregate-containing, AGG+, or diffused, AGG-) either expressed alone (as in Fig. 1), or with DsRed co-expression (Fig. 6). (b) Expression of genes belonging to the HSP70 family of chaperones[3] was significantly repressed in cells containing mutFUS aggregates co-expressing DsRed (AGG+, solid blue line), while no such repression was seen in cells with diffused expression of mutFUS (AGG-, dashed blue line). Plots show the cumulative distribution function (CDF, y-axis) of LFC values belonging to the HSP70 chaperone family (using two definitions, see Methods), compared to the background CDF of all expressed mRNAs (background, in grey lines). P-value for the differences of each sample compared to the background distribution was calculated using t-test (two-sided), and is indicated in the legend when significant. (c) Clustergram of the correlation matrix of z-score normalized mean TPM values of all differentially expressed genes (DEGs) in the experiment, shows very high correlation between cells co-expressing DNAJB14-short and cells co-expressing DsRed. (d) CDF plots (CDF, y-axis) of the Log2 Fold Change (LFC) values of different samples divided by mutFUS AGG- DsRed, show that co-expression of DNAJB14-FL in mutFUS AGG+ cells (red) restores the distribution of mRNA expression changes that occurred in the presence of mutFUS AGG+ cells in DsRed (blue) or DNAJB14-short (green) co-expressing cells, almost to the background (grey) distribution. Left panel – red cluster from Fig. 6a, right panel – blue cluster from Fig. 6a. p-values were calculated as in (b). (e-h) CDF plots (CDF, y-axis) as in (d), but for different chaperone families (HSP70, HSP60s, HSP90s and ER-related chaperones, as defined by Brehme et al.[3]) show that co-expression of DNAJB14-FL in mutFUS AGG+ cells restored the down-regulation in the expression of these chaperones in mutFUS AGG+ cells co-expressing DsRed or DNAJB14-short. (i) Scatter plots show no correlation in mRNA expression changes between cell subjected to heat stress (X-axis) and mutFUS AGG+ (left panel) or AGG- (right panel) cells co-expressing DNAJB14-FL. (j-k) HSR promoter activation score (Y-axis, see Methods) vs. mutFUS aggregation modulation scores (j) or HTT-polyQ Aggregation modulation scores (k) (X-axis), show no correlation. Pearson correlation coefficient (CC) is presented. Inset – zoom in on the cloud around the zero point, indicating some specific chaperones. (l) qPCR of HEK293T cells transfected with each one of the different chaperones showing Hspa1a expression (normalized to HPRT). Data are presented as mean values +/- SEM for n=2 biologically independent experiments. Source data are provided as a Source Data file. (m) HEK293T cells co-transfected with FUS-R521H-YFP and DsRed, and then treated with different heat stress regimens; Heat shock of 44C for 1.5h with additional 18h recovery at 37C, 44C for 2h, or 42C for 8h, followed by PulSA analysis at 48h post transfection and right after the treatment. All three regimens showed no aggregation amelioration. Data are presented as mean values +/- SEM for n=6 biologically independent samples for the recovery experiment, and n=5 for 2h at 44C and 8h at 42C. Source data are provided as a Source Data file.

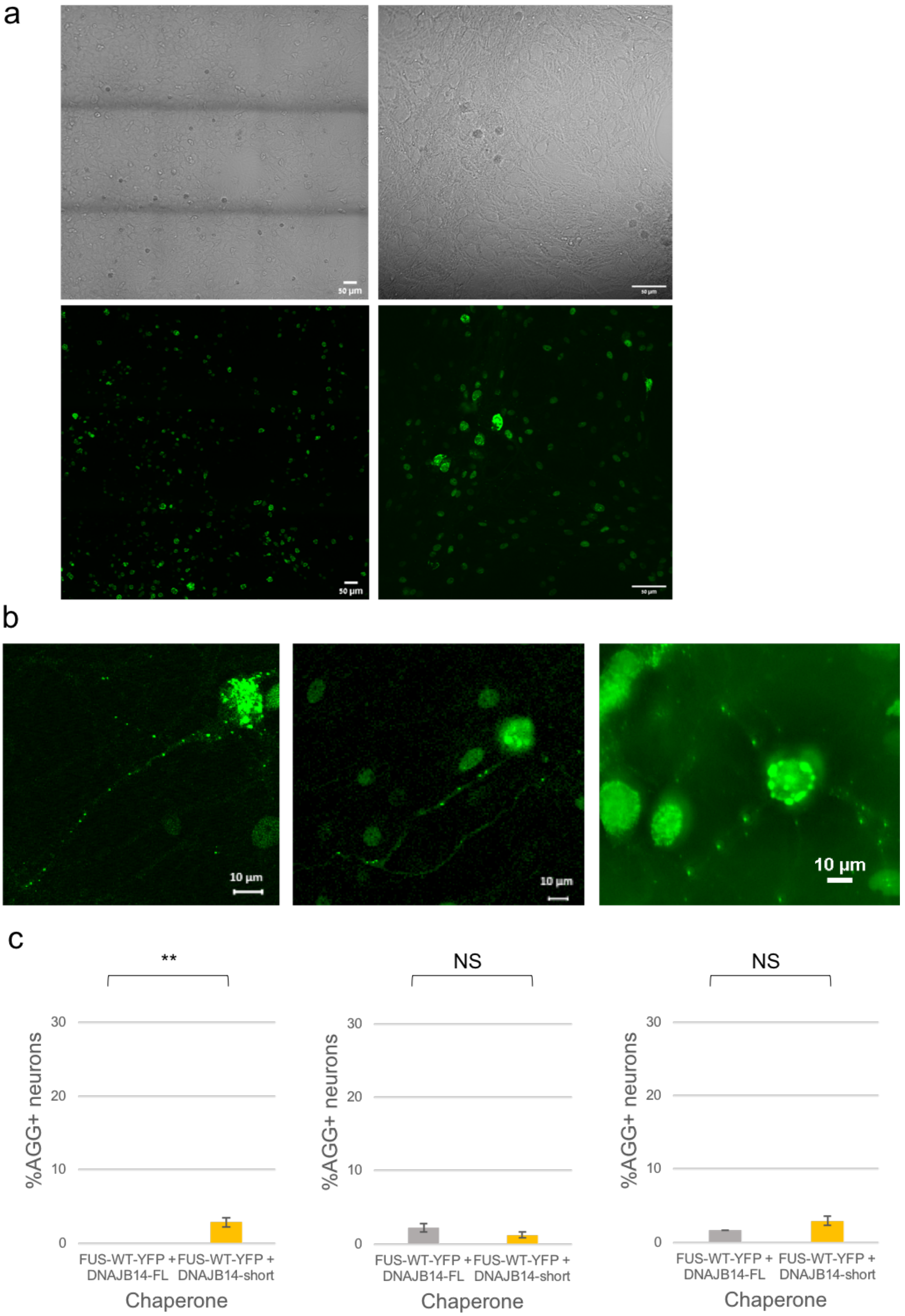

a
50 µm
50 µm
b
10 µm
10 µm
10 µm
c
**
NS
NS
%AGG+ neurons
30
20
10
0
FUS-WT-YFP + DNAJB14-FL
FUS-WT-YFP + DNAJB14-short
Chaperone

d

FUS-WT-YFP+DNAJB14-short FUS-WT-YFP+DNAJB14-FL

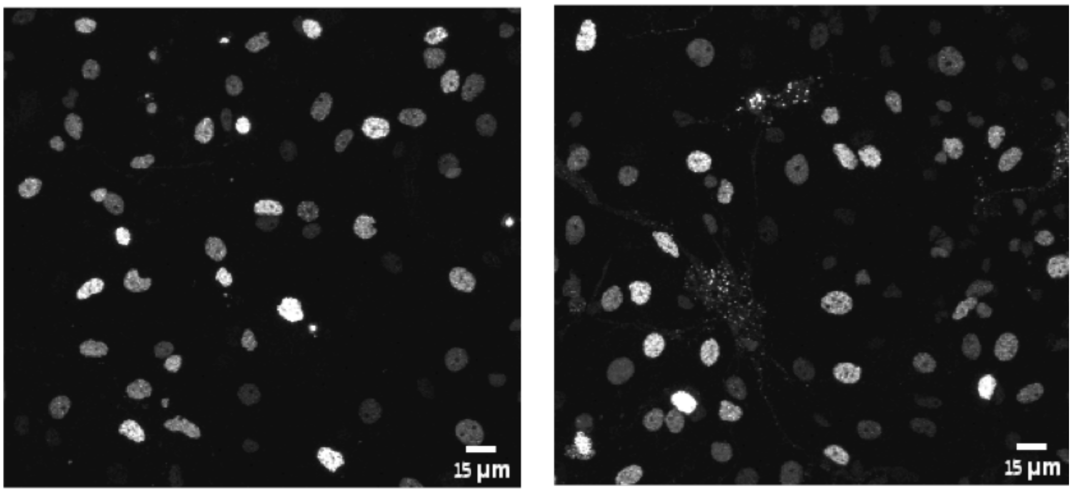

e

FUS-R521H-YFP+DNAJB14-short FUS-R521H-YFP+DNAJB14-FL

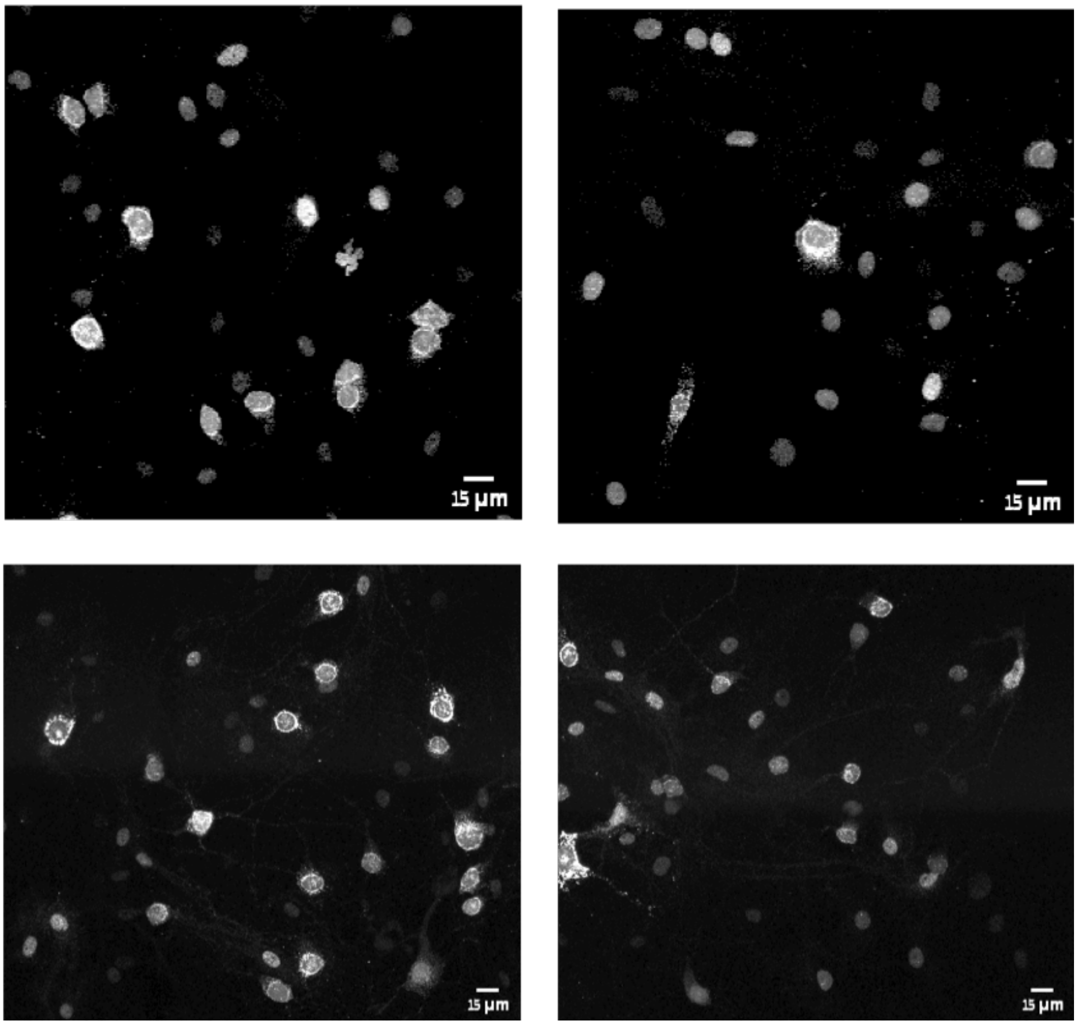

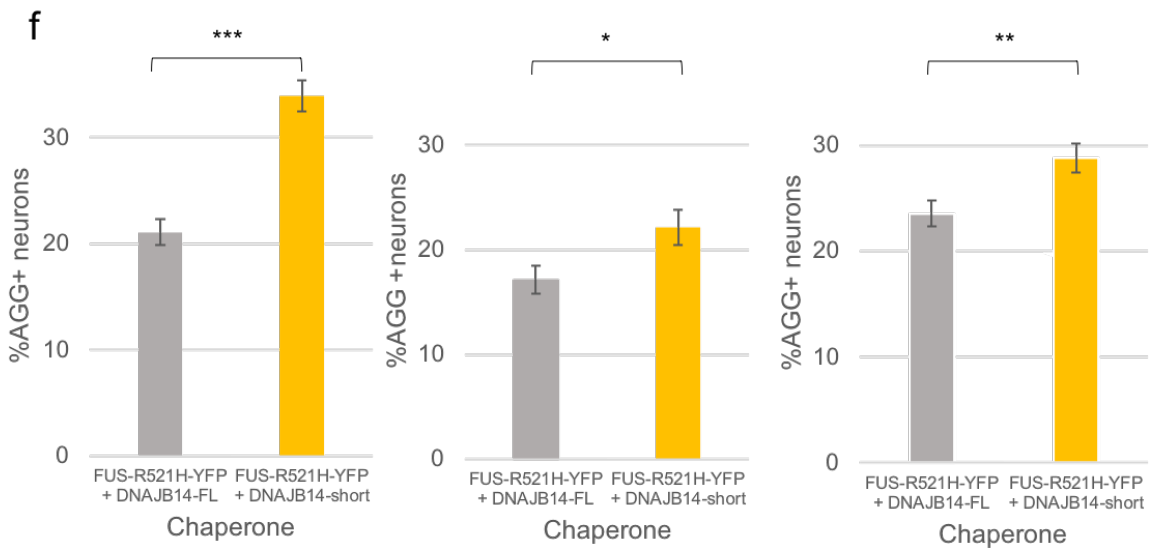

Supplementary Figure 10: DNAJB14 isoforms show differential modulation of mutant FUS aggregation in primary neurons.

(a-b) Bright field (top) and fluorescence confocal microscopy of live neuronal cultures infected with FUS-R521H-YFP using the AAV2 viral system. (a) Mutant FUS-R521H-YFP can also be observed in neuronal projections. (b) Images were taken either using confocal microscopy (left and middle) or using the HDR software module on a regular Nikon microscope to integrate different exposure times for different regions in the same image (right panel). Shown are representative fields from n=5 biologically independent experiments. (c) Image analysis of thousands of live neurons using confocal microscopy images in FUS-WT-YFP expressing neurons, co-infected with either DNAJB14-FL or DNAJB14-short. Cells were infected with both FUS-WT-YFP and DNAJB14-FL or DNAJB14-short containing viruses at day 5 of culture (titer matched), and imaged six days later. Data are presented as mean values +/- SEM of n=3 biologically independent samples, each containing 16 imaged fields for each of DNAJB14-FL and DNAJB14-short neuronal cultures (containing 741 and 883, 659 and 689, 1131 and 1208 neurons for DNAJB14-FL and -short respectively). Mean fraction of aggregate-containing cells was very low for both types (0-2.96%) and no consistent difference was observed between DNAJB14-FL and DNAJB14-short cultures (p=5e-4, 0.15 0.12 for the three experiments respectively, using t-test, two-sided, non-adjusted). NS-Non significant. Source data are provided as a Source Data file. (d) Fluorescence confocal microscopy images of live neurons infected with FUS-WT-YFP and DNAJB14-FL or DNAJB14-short viruses. Shown are maximum projection images. Shown are representative fields from n=3 biologically independent experiments. (e) Fluorescence confocal microscopy images of live neurons infected with FUS-R521H-YFP and DNAJB14-FL or DNAJB14-short viruses, additional fields, as in Fig. 7c, maximum projection images. (f) Image analysis of thousands of live neurons using confocal microscopy images in FUS-R521H-YFP expressing neurons, co-expressing either DNAJB14-FL or DNAJB14-short. Cells were infected with both FUS-R521H-YFP and DNAJB14-FL or DNAJB14-short containing viruses at day 5 of culture, and imaged six days later. Data are presented as mean values +/- SEM of n=3 biologically independent experiments, containing 12, 24 and 37 imaged fields for each of DNAJB14-FL and DNAJB14-short neuronal cultures (with 884 and 1081, 1339 and 1069, 2281 and 2231 neurons for DNAJB14-FL and -short respectively). Mean fraction of aggregate-containing cells was consistently and significantly lower in all replicates for the DNAJB14-FL infected neurons (p=7.9e-7, 0.023 and 5.7e-3 for the three experiments respectively, calculated using t-test, two-sided, non-adjusted). Source data are provided as a Source Data file.

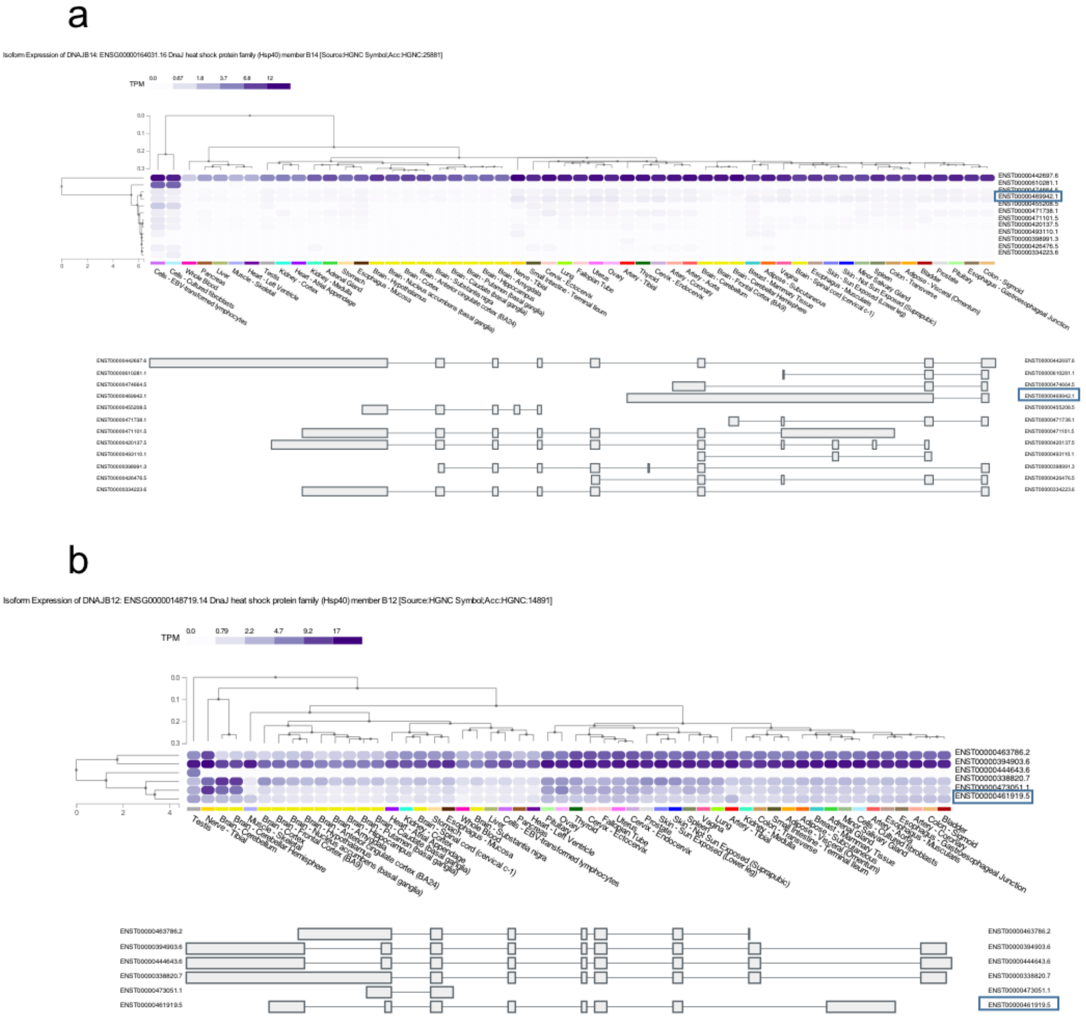

Supplementary Figure 11: DNAJB12 and DNAJB14 isoform expression in human adult tissues

Figures were generated using the GTEx portal, for DNAJB14 (a) and DNAJB12 (b) isoforms.

	Aggregation Modulation score, Log2(AGG+ chaprone/AGG+ control)	
	HTT-134Q-GFP	FUS-R521H-YFP
DNAJC5	-0.113307	-3.729123
DNAJB12-FL	0.1968	-2.364345
DNAJC5B	-0.052375	-1.496803
DNAJB14	0.35167	-0.820585
BAG3	0.40197	-0.589478
HSF1	-0.094859	-0.529681
DNAJC30	-0.106081	-0.446112
HSPA1A	0.036035	-0.275731
DNAJC18	0.291406	-0.274582
DNAJB3	-0.001949	-0.227938
DNAJB12-short	-0.1721	-0.216087
DNAJC25	0.194227	-0.198707
BAG4	0.090443	-0.173658
DNAJC7	0.134615	-0.164522
DNAJC8	0.180654	-0.15035
DNAJC11	0.11339	-0.130547
STUB1	-0.050036	-0.120089
DNAJB6-FL	0.093471	-0.10904
BAG6	0.291876	-0.097508
DNAJB11	-0.070419	-0.09708
DNAJB1	0.002933	-0.089131
DNAJA4	-0.023463	-0.086803
HSPA4L	0.39252	-0.075485
NACA2	0.022559	-0.072791
HSPB7	-0.184632	-0.070836
HSP90B1	0.2014	-0.064825
DNAJC4	0.184967	-0.048632
NACA	0.10543	-0.02292
HSPA2	0.072448	-0.020248

BAG1	0.11289	-0.011521
HSPBP1	0.274247	0.003268
HSPB2	-0.041814	0.004548
BAG2	0.203127	0.010261
BTF3	0.027953	0.010796
DNAJA1	0.306336	0.022616
BAG5	0.331059	0.025385
HSPA9	0.560907	0.032971
DNAJB6-short	-0.359926	0.037348
DNAJC27	0.125898	0.040014
HSPA6	0.252707	0.047343
DNAJC12	0.043845	0.051139
DNAJC24	0.06382	0.05479
DNAJC10	0.208447	0.061539
DNAJC15	0.071714	0.063922
DNAJA2	-0.006321	0.073894
HIP1	0.303613	0.075073
DNAJC1	0.033051	0.075224
DNAJA3	-0.050989	0.084366
DNAJC22	0.252113	0.095148
DNAJC16	0.145377	0.098937
DNAJC17	0.21594	0.101648
DNAJC19	0.1494	0.110745
DNAJC6	0.190963	0.113984
HSPA4	0.251961	0.116909
DNAJB2b	0.312427	0.137794
DNAJB4	0.047865	0.138461
DNAJC3	0.203423	0.140922
DNAJC28	0.175631	0.143392
DNAJB9	0.035527	0.143933
DNAJB8	-1.00032	0.15541
HSP90AB1	0.293016	0.169219

HSPH1	0.206341	0.178009
DNAJC14	0.17258	0.195298
HSPA14	0.26328	0.209752
HSP90AA1	0.18037	0.26606
DNAJB5	-0.002481	0.306305

Supplementary Table 1

Aggregation modulation score for 66 chaperones that participated in the aggregation modulation screens, with respect to HTT-134Q or FUS-R521H-YFP.

	Primers used in this study	Primer type
B14_Sh_F	GGGGACAAGTTTGTACAAAAAAGCAGGCTTC<br>ATGGAGGGGAACAGGGATG	cloning
B14_Sh_R	GGGGACCACTTTGTACAAGAAAGCTGGGTT<br>CCTGAGAACTCCATCTACTTGGTCTT	cloning
B14_duf_F	GGGGACAAGTTTGTACAAAAAAGCAGGCTTCATGGAGGGGAACAGGG	cloning
B14_duf_R	GGGGACCACTTTGTACAAGAAAGCTGGGTT<br>GACCATCAACTGGCTTAAT	cloning
B14_HPDmut_t408a	atggtttttgtctggttgaaacttcaaagcaagctttctataagc	mutagenesis
	gcttatagaaagcttgctttgaagtttcaaccagacaaaaaccat	mutagenesis
B14_HPDmut_t408g	atggtttttgtctggctgaaacttcaaagcaagctttctataagc	mutagenesis
	gcttatagaaagcttgctttgaagtttcagccagacaaaaaccat	mutagenesis
B14- Δshort F	GGGGACAAGTTTGTACAAAAAAGCAGGCTTC<br>ATGATAAACAAATGTAAAAATTACTATGAAGTA	cloning
B14-Δshort R	GGGGACCACTTTGTACAAGAAAGCTGGGTT<br>TCCTCCTTTATAAAGACTGGTAAG	cloning
HSPA7 F	atacgcgttgGGATCCGACCTCCCACAGCCCCGG	cloning
HSPA7 R	atttcgataaCTTGTCGGATGCTGGAGGCCACGGA	cloning
HSPA1A F	ccccaccattgaggaggt	qPCR
HSPA1A R	tcaacattgcaaacacaggaa	qPCR
HPRT F	TGACACTGGCAAAACAATGCA	qPCR
HPRT R	GGTCCTTTTCACCAGCAAGCT	qPCR
HSPA5 F RAT	TGCAGCAGGACATCAAGTTC	qPCR
HSPA5 R RAT	TTTCTTCTGGGGCAAATGTC	qPCR
PSMD 12 F RAT	GAAAGTGACAAGTGGCAGCA	qPCR
PSMD 12 R RAT	CCGGTGAACCAAATCTGACT	qPCR

BAG4 F RAT	GGCACTTACTCCACGGACAT	qPCR
BAG4 R RAT	CAGTCCTGCTGGGGATACAT	qPCR
DNAJB11 F RAT	TACCTCTCCCTCTGGGGATT	qPCR
DNAJB11 R RAT	TGACCCTCATCTGTGCTCTG	qPCR
DNAJC2 F RAT	AGGAAGAAGGAAAGGCCAAG	qPCR
DNAJC2 R RAT	AGCCAACCGAACAGCTTCTA	qPCR
DNAJC3 F RAT	GATTGGGTCAGCTGAAGAGC	qPCR
DNAJC3 R RAT	GCGGACTGTGTACTCAGCAA	qPCR
HSP90AB1 F RAT	ACCCCATTGTGGAGACTCTG	qPCR
HSP90AB1 R RAT	AGCAGTCTCAAACAGCAGCA	qPCR
HSPA9 F RAT	GACAGGACGTGAGCAACAGA	qPCR
HSPA9 R RAT	TCTTCCTCCGGTCTTCTTCA	qPCR
HPRT F RAT	TCCAACACTTCGAGAGGTCCTTTTCAC	qPCR
HPRT R RAT	GGGGGCTATAAGTTCTTTGCTGACC	qPCR
FUS p.R521C	gcctctccctgcaatcctgtctgtgctca	mutagenesis
	tgagcacagacaggattgcagggagaggc	mutagenesis
FUSp.R495X	gaaagctgggtccaagaagcctccacggtc	mutagenesis
	gaccgtggaggcttcttggacccagctttc	mutagenesis
FUS p.R495EfsX527	cccggccccctcgaagcctccacg	mutagenesis
	cgtggaggcttcgagggggccggg	mutagenesis
FUS isoforms stage2 - Frame shift	cagggagaggccgtattttggacccagcttt	mutagenesis
	aaagctgggtccaaaatacggcctctccctg	mutagenesis

Supplementary Table 2

Primers used in this study.

Supplementary References:

1. Ramdzan YM*, et al.* Tracking protein aggregation and mislocalization in cells with flow cytometry. *Nat Methods* **9**, 467-470 (2012).

2. Sabath N*, et al.* Cellular proteostasis decline in human senescence. *Proc Natl Acad Sci U S A* **117**, 31902-31913 (2020).

3. Brehme M*, et al.* A chaperome subnetwork safeguards proteostasis in aging and neurodegenerative disease. *Cell Rep* **9**, 1135-1150 (2014).
