# Supplementary material for: Differential roles for DNAJ isoforms in HTT-polyQ and FUS aggregation modulation revealed by chaperone screens
Source: Nat Commun. 2022 Jan 26;13:516. doi: 10.1038/s41467-022-27982-w (PMC8792056; doi:10.1038/s41467-022-27982-w)
Supplement: Supplementary file 2 — Description of Additional Supplementary Files [file 41467_2022_27982_MOESM2_ESM.pdf]

Description of Additional Supplementary Files

File name: Supplementary Data 1

Description: Gene expression as Transcript Per Million values (TPM, RSEM output) and the results of differential expression analysis (DEseq2 output), which include P-value (pvalue), False Discovery Rate (padj) and LFC (log2FoldChange) for the RNA-seq presented in Figure 1 (a) and Figure 6 (b). Additionally, the table specifies whether the genes were defined as expressed or as significantly differential (as specified in the Methods), and denotes whether the genes belong to the HTT-only induction cluster (Fig. 1b) or Restoration clusters (Fig. 6a).

File name: Supplementary Movie 1

Description: Fluorescence confocal microscopy of live neuronal cultures infected with FUS-R521H-YFP. The Imaris software was used to reconstruct 3D visualizations of cells.

File name: Supplementary Movie 2

Description: Fluorescence confocal microscopy of live neuronal cultures infected with FUS-R521H-YFP. The Imaris software was used to reconstruct 3D visualizations of cells.

File name: Supplementary Movie 3

Description: Fluorescence confocal microscopy of live neuronal cultures infected with FUS-WT-YFP. The Imaris software was used to reconstruct 3D visualizations of cells.
