# Supplementary material for: Differential roles for DNAJ isoforms in HTT-polyQ and FUS aggregation modulation revealed by chaperone screens
Source: Nat Commun. 2022 Jan 26;13:516. doi: 10.1038/s41467-022-27982-w (PMC8792056; doi:10.1038/s41467-022-27982-w)
Supplement: Supplementary file 7 — Reporting Summary [file 41467_2022_27982_MOESM7_ESM.pdf]

nature research

Corresponding author(s): Reut Shalgi

Last updated by author(s): Oct 19, 2020

Reporting Summary

Nature Research wishes to improve the reproducibility of the work that we publish. This form provides structure for consistency and transparency in reporting. For further information on Nature Research policies, see our Editorial Policies and the Editorial Policy Checklist.

Statistics

For all statistical analyses, confirm that the following items are present in the figure legend, table legend, main text, or Methods section.

n/a	Confirmed	
☐	☒	The exact sample size (n) for each experimental group/condition, given as a discrete number and unit of measurement
☐	☒	A statement on whether measurements were taken from distinct samples or whether the same sample was measured repeatedly
☐	☒	The statistical test(s) used AND whether they are one- or two-sided<br>*Only common tests should be described solely by name; describe more complex techniques in the Methods section.*
☒	☐	A description of all covariates tested
☒	☐	A description of any assumptions or corrections, such as tests of normality and adjustment for multiple comparisons
☐	☒	A full description of the statistical parameters including central tendency (e.g. means) or other basic estimates (e.g. regression coefficient) AND variation (e.g. standard deviation) or associated estimates of uncertainty (e.g. confidence intervals)
☐	☒	For null hypothesis testing, the test statistic (e.g. F, t, r) with confidence intervals, effect sizes, degrees of freedom and P value noted<br>*Give P values as exact values whenever suitable.*
☒	☐	For Bayesian analysis, information on the choice of priors and Markov chain Monte Carlo settings
☒	☐	For hierarchical and complex designs, identification of the appropriate level for tests and full reporting of outcomes
☐	☒	Estimates of effect sizes (e.g. Cohen's d, Pearson's r), indicating how they were calculated

Our web collection on statistics for biologists contains articles on many of the points above.

Software and code

Policy information about availability of computer code

Data collection	*Kaluza Analysis Software 2.1*
Data analysis	*Aligment software: STAR (RNA-seq aligner) software , RSEM (RNA-Seq by Expectation-Maximization) software, Annotations downloaded from UCSC genome browser website, Analysis software: R language + RStudio, R packages:DESeq2,RDAVIDWebService, tidyverse, ggplot2, ComplexHeatmap. Image analysis: FIJI-WIN64*

For manuscripts utilizing custom algorithms or software that are central to the research but not yet described in published literature, software must be made available to editors and reviewers. We strongly encourage code deposition in a community repository (e.g. GitHub). See the Nature Research guidelines for submitting code & software for further information.

Data

Policy information about availability of data

All manuscripts must include a data availability statement. This statement should provide the following information, where applicable:

- Accession codes, unique identifiers, or web links for publicly available datasets
- A list of figures that have associated raw data
- A description of any restrictions on data availability

The datasets generated during the current study are included in this published article and its supplementary information files. The raw FACS data files used in this study to generate Aggregation Modulation Scores are available from the corresponding author upon request, and numeric data are available in the Source Data file. The RNA-seq data generated in this study have been deposited in the GEO database under accession number GSE165317 (https://www.ncbi.nlm.nih.gov/geo/query/acc.cgi?acc=GSE165317). All other data are available in the Source Data file.

Field-specific reporting

Please select the one below that is the best fit for your research. If you are not sure, read the appropriate sections before making your selection.

☒ Life sciences ☐ Behavioural & social sciences ☐ Ecological, evolutionary & environmental sciences

For a reference copy of the document with all sections, see nature.com/documents/nr-reporting-summary-flat.pdf

Life sciences study design

All studies must disclose on these points even when the disclosure is negative.

Sample size	Sample sizes and replicates are indicated in the manuscript, figures, figure legends and Methods.
Data exclusions	*No data were excluded*
Replication	Sample sizes and replicates are indicated in the manuscript, figures, figure legends and Methods. All attempts at replication were successful
Randomization	*NA*
Blinding	*All data was analyzed automatically*

Reporting for specific materials, systems and methods

We require information from authors about some types of materials, experimental systems and methods used in many studies. Here, indicate whether each material, system or method listed is relevant to your study. If you are not sure if a list item applies to your research, read the appropriate section before selecting a response.

Materials & experimental systems

n/a	Involved in the study	
☐	☒	Antibodies
☐	☒	Eukaryotic cell lines
☒	☐	Palaeontology and archaeology
☐	☒	Animals and other organisms
☒	☐	Human research participants
☒	☐	Clinical data
☒	☐	Dual use research of concern

Methods

n/a	Involved in the study	
☒	☐	ChIP-seq
☐	☒	Flow cytometry
☒	☐	MRI-based neuroimaging

Antibodies

Antibodies used	anti-FLAG antibody (Sigma-Aldrich, F1804), anti-HSC70/HSP70 (Enzo Life Sciences, N27F34), anti-GFP (MBL, 598),anti-RFP (ABCAM ab34771),rabbit igG cell signaling 2729S,AlexaFluor 647 Donkey Anti-Mouse (Jackson),secondary antibody FC fragment specific (Jackson 111-035-046),secondary antibody LC fragment specific(Jackson 111-035-071)
Validation	*antibody validation was performed by the manufacturer.*

Eukaryotic cell lines

Policy information about cell lines

Cell line source(s)	HEK293T, ATCC
Authentication	*Cells were not authenticated*
Mycoplasma contamination	tested negative
Commonly misidentified lines (See ICLAC register)	*Name any commonly misidentified cell lines used in the study and provide a rationale for their use.*

Animals and other organisms

Policy information about studies involving animals; ARRIVE guidelines recommended for reporting animal research

Laboratory animals	P0 neonates, from timed-pregnant white coat female Sprague Dawley female rats (Charles River; SAS SD, strain code 400)
Wild animals	*No wild animals were used in the study.*
Field-collected samples	*NA*
Ethics oversight	Ethics certificate No IL-130-09-17

Note that full information on the approval of the study protocol must also be provided in the manuscript.

Flow Cytometry

Plots

Confirm that:

☒ The axis labels state the marker and fluorochrome used (e.g. CD4-FITC).

☒ The axis scales are clearly visible. Include numbers along axes only for bottom left plot of group (a 'group' is an analysis of identical markers).

☒ All plots are contour plots with outliers or pseudocolor plots.

☒ A numerical value for number of cells or percentage (with statistics) is provided.

Methodology

Sample preparation	Cells were trypsinized, resuspended in media with serum, placed on ice, and DAPI regent was added (5µM) in order to assay cell viability
Instrument	FACSAria™ IIIu (from BD Biocsiences)
Software	Kaluza Analysis Software 2.1 (BECKMAN COULTER)
Cell population abundance	*The purity of the instrument is above 98%. No specific analysis of purity was performed.*
Gating strategy	FSC/SSC Preliminary,<br>Dapi/ FSC for live and dead,<br>FSC-H/FSC-W & SSC-H/SSC-W for doublet discrimination<br>GFP-H/GFP-W for aggregates.

☒ Tick this box to confirm that a figure exemplifying the gating strategy is provided in the Supplementary Information.
